# Understanding why people value working from home or hybrid workplace flexibility a study of preferences during the COVID-19 pandemic

**DOI:** 10.1371/journal.pone.0348206

**Published:** 2026-05-04

**Authors:** Courtney Bir, Jinho Jung, Nicole Olynk Widmar

**Affiliations:** 1 Dept. of Agricultural Economics, Oklahoma State University, Stillwater, Oklahoma, United States of America; 2 Division of Humanities and Social Sciences, Pohang University of Science and Technology (POSTECH), Pohang, Gyungbuk, Republic of Korea; 3 Department of Agricultural Economics, Purdue University, West Lafayette, Indiana, United States of America; University of Crete, GREECE

## Abstract

Working-From-Home (WFH) practices expanded rapidly during the COVID-19 pandemic and continue to be a point of discussion today with debates increasingly focused on productivity rather than the underlying reasons for WFH or flexibility. This study investigates why individuals value WFH and hybrid work arrangements in the United States. The specific period of study was during the COVID-19 pandemic. Understanding these motivations can inform constructive negotiations and effective policies that enhance productivity while supporting employees’ work–life balance and caregiving responsibilities. Despite extensive discussion of whether employers should permit WFH, the diverse reasons employees seek flexibility remain understudied. Using data from a nationally representative online survey conducted in late 2021, we employ a best–worst scaling experiment to rank motivations for remote work. Results show the most valued reason for WFH is balancing work with caregiving, followed by reducing commuting time and costs, limiting exposure to illness, and preferring the home environment. A latent class model identifies four heterogeneous preference segments: (1) caregiving and commuting, (2) productivity and comfort, (3) multitasking and health safety, and (4) diffuse preferences without a dominant motivation. Additionally, seemingly unrelated regression analysis links WFH preferences with behavioral changes in grooming, attire, and personal care routines. These findings highlight the heterogeneity in workers’ motivations for flexibility and suggest that one-size-fits-all approaches may be inefficient. By revealing the underlying drivers of WFH preferences, this study offers nuanced insights for organizations seeking to design flexible work policies that balance productivity objectives with employee well-being.

## Introduction

Early in the COVID-19 pandemic governments suggested, and then implemented, social distancing to slow the spread of infection [1–3]. Companies adopted Work-From-Home (WFH) policies to facilitate continuing operations while complying with distancing and infection reduction practices [4–6]. As a result, the number of workers conducting their work at home substantially increased beginning in March 2020 [5,7–14]. Many ex-post empirical analyses generally confirmed that WFH mitigated the spread of the pandemic and/or at least lessened the negative effect of the pandemic on economic outcomes, production, and employment [7,15–20].

As the acute phases of the COVID-19 pandemic came to an end, so did the benefits of WFH in mitigating the negative effects of the pandemic on health and of social distancing policies on economic activities. Meanwhile, as the necessary technologies for WFH improved and were widely adopted, employers and employees became more comfortable with WFH, and the practice became more feasible and sustainable. As a result, WFH practices still continue, albeit at varying rates across different industries and companies, with several options debated between companies and employees, including: fully remote work, hybrid-which may include mandated days in the office, or fully Return-To-Office (RTO). After the U.S. government’s declaration of the end of the pandemic public health emergency on May 11, 2023 [21,22] the debate over WFH has continued to heat up with discontent on all sides.

Most discussions on WFH from previous studies are rooted in consequential aspects of it such as 1) work-environment and productivity [23–28], 2) employment relations/job insecurity [29,30], 3) dependence of productivity on characteristics of occupations/tasks under the WFH practice [31–34], 4) gender division/gaps/inequality [35–38], and 5) well-being in terms of psychological and mental health [39–43]. Overall, the literature has not focused substantially on the preferences and motivations behind WFH choices.

Companies have offered a variety of WFH versus RTO options, often adapting them over time. Announcements of RTO three days per week, or on set days (Tue, Wed, and Thur in office) to facilitate team interactions, or a specific number of days over the course of a month. The specific requirements of RTO have been varied and rapidly changing. Some tech companies are still pursuing a 100% remote option and proponents say that WFH boasts a better work-life balance and saves time wasted on commutes [44]. On the other hand, some companies and business leaders want their people to RTO. Such resurgence of office occupancy has primarily been driven by Wall Street firms for collaborative apprenticeship, immediate communication/follow-up/feedback, effective decision makings, and/or improvement in learning/creativity [45]. For example, JPMorgan Chase and Goldman Sachs made 5-day RTO compulsory [46–48]. Amazon’s CEO, Andy Jassy, announced a RTO mandate in Sep of 2024 [46,49]. A survey also noted that 68% of managers believe that their remote workers miss out on impromptu and/or informal feedback [50,51]. Additionally, ninety four percent of workers say they can be convinced to return to their office [50–52].

Findings from the discussions surrounding WFH policies are inconsistent [51–53]. RTO mandates and movements back into the office led to fully remote workers having decreased from 34% in 2022 to 7% in 2023 [51]. A more recent survey found that the overall share of paid workdays that are WFH decreased by less than half a percentage point from 21.2% to 20.8% [52]. Bloom et al. [54] also reported that the US firms do not see material trend to RTO even under RTO mandates.

Meanwhile, other studies continue to discuss employees’ preferences for working modality such as continuing WFH/hybrid or mandating RTO in the future. For example, sixty-six percent of employers prefer to mandate a fully committed in-office working environment, while 41% of workers prefer to work fully remotely, and 37% of workers prefer a hybrid model [51]. Such discussions often end up asserting which modality employers and employees prefer only in terms of productivity, such as which style is more productive under what conditions, without reasoned arguments because measuring impact of different working styles on productivity is neither easy/straightforward nor as accurate as expected/needed [8,31,33,34,55]. In other words, reasons behind why employees or workers need or prefer to work from home have not yet been the focus. Thus, debates continue without adequate information to address why individuals prefer WFH and/or what can be done to accommodate those needs/wants while addressing the concerns of employers simultaneously.

Employees allowed flexibility can adjust their working time so they can fulfill other demands in their life, including caretaking or family matters. Even before the COVID-19 pandemic and the growth in WFH practices, several studies revealed that job flexibility in general could reduce work-life balance conflicts, helping employees lower the distress of not fulfilling family responsibilities [56,57]. Other studies emphasize the importance of managing psychological well-being for maintaining productivity in the workplace [58–60] and work-life balance is a significant element that affects psychological well-being [61]. Giving employees autonomy at home, along with control of their boundaries, such as whether they conduct non-work-related activities during working hours, may be a concern for employers [62]. Almost half (46%) of workers polywork with at least a side hustle, and 36% of workers are planning to start an additional job in the future [51].

This study revisits a period of time when many people had work from home opportunities, late 2021, evaluating which reasons are most or least important to individuals with respect to WFH practices. Although WFH policies have changed, and some motivations may have shifted (i.e., reducing viral exposure may be relatively less important), the general top motivators found for work from are still relevant to decision makers today. Eliciting the motivation behind employees’ preferences on WFH is helpful to facilitate constructive and effective communication between employers and employees to find a better solution for both parties. Delving into such reasons is expected to provide more detailed understanding of why groups of people with different demographic characteristics and living conditions prefer WFH. This would help employers tailor WFH policies for their employees to enhance their productivity and quality of life. Within the attributes studied, facilitating balance of work with caregiving responsibilities rose to the top, with simplified grooming and saving money on lifestyle choices such as eating lunches out falling to the bottom. Further latent class analysis garnered more detailed results for quantifiable segments of workers. Ongoing public debates about WFH being offered or taken away oversimplifies the experiences of both employers and employees; understanding the reasons that WFH is valued or desired can facilitate productive conversations between employers and employees.

## Methods

### Data collection

The online survey tool Qualtrics was used to gather information from November 30, 2021 to December 8^th^, 2021, from 751 U.S. residents [63]. The survey was designed to collect basic demographic information as well as information regarding work from home preferences and changes in personal behavior related to work from home. Kantar, a large opt-in panel provider, was used to obtain survey respondents [64]. Respondents were required to be 18 years of age or older to participate. The sample was targeted to be representative of the U.S. population in terms of gender, income, education, and geographical region of residence [65]. Regions of residence were defined as in the Census Bureau Regions and Divisions (Table 1). All respondents were asked about personal behavioral changes pre and post March 2020, when many areas instituted COVID-19 lockdowns. Only respondents who reported that they worked, n = 435, were asked specific questions regarding work location preferences. All participants provided informed consent prior to participation in the survey. Consent was obtained in written form via the survey questionnaire, and participants indicated their agreement before proceeding to the survey questions. No minors were included in the study.

**Table 1:** Definition of regions.

Region	States included
Northeast	Connecticut, Maine, Massachusetts, New Hampshire, Rhode Island, Vermont, New Jersey, New York, and Pennsylvania
Midwest	Indiana, Illinois, Michigan, Ohio, Wisconsin, Iowa, Kansas, Minnesota, Missouri, Nebraska, North Dakota, and South Dakota
South	Delaware, District of Columbia, Florida, Georgia, Maryland, North Carolina, South Carolina, Virginia, West Virginia, Alabama, Kentucky, Mississippi, Tennessee, Arkansas, Louisiana, Oklahoma, and Texas
West	Arizona, Colorado, Idaho, New Mexico, Montana, Utah, Nevada, and Wyoming, Alaska, California, Hawaii, Oregon, and Washington

### Best worst scaling

A best worst scaling experiment was completed by respondents who worked (n = 435). They were asked to select the most important and least important reason for working remotely from home. Reasons included: saving money on lifestyle choices, such as eating lunches out; saving or reallocating commuting time and expenses; desire to reduce viral/disease exposure for self and/or household members; facilitating balance of work with caregiving responsibilities for children, family members, and/or pets; more productive at home; prefer the home environment; simplified or lessened grooming or dressing costs, in time and/or money, to work from home or remotely; prefer the ability to intermingle work and home activities. Respondents were presented with 4 of the 8 attributes included in this study and had to choose the most and least important. This task was repeated 14 times. The presentation of attributes and overall design was determined using a balanced incomplete block design using the program SAS. The chosen design had a d-efficiency of 85.7. The respondent’s choices of the most important and least important reasons for working from home were used to determine each reason’s location along a continuum. For reason *j*, the location of the reason on the scale of most important to least important is represented by $\lambda_{\text{j}}$. Therefore, the random value of the utility difference between reasons *(j, k)* ϵ *(1,…,J), j ≠ k* for the maximum difference multinomial logit model is:


$${\text{U}}_{\text{jk}}=\lambda_{\text{j}}+\epsilon_{\text{jk}}$$ (1)

where $\epsilon_{\text{jk}}$ represents a random error term which are i.i.d. type 1 extreme value. The probability of choosing a given most important-least important combination takes the multinomial logit (MNL) form [66] represented by:


$$Prob\left(j=best\;⋂k=worst\right)=\frac{e^{\lambda_{j}-\lambda_{k}}}{\sum_{l=\text{1}}^{J}\sum_{m=\text{1}}^{J}e^{\lambda_{l}-\lambda_{m}}-J\;}$$ (2)

The parameter $\lambda_{\text{j}}$, estimated using maximum-likelihood estimation (maxdiff), represents how important reason *j* is relative to the least important reason. One reason must be normalized to zero in order to prevent multicollinearity [67].

In addition to the maxdiff MNL model, a random utility representation of the maxdiff model for BWS was evaluated (maxdiff RPL) to allow for continuous heterogeneity among respondents. The shares of preferences are calculated as:


$${share}_{j}=\frac{e^{\lambda_{j}}}{\sum_{k=\text{1}}^{J}e^{\lambda_{k}}}$$ (3)

and must necessarily sum to one across the 8 reasons. Estimations were conducted using NLOGIT 6.0.

Confidence intervals for the preference shares were determined using the method proposed by Krinsky-Robb [68]. Confidence intervals were used to evaluate statistical differences for each reason to establish a numerical ranking using the overlapping confidence interval method [69].

The latent class model (LCM) classifies individuals into one of the classes (*S*), based on their preferences. Within each class, preferences are homogenous; but preferences are heterogeneous across classes [70]. Individual respondents are assigned to an unobserved latent class after parameters for each class are simultaneously estimated [71]. Given the respondent belongs to a specific latent class, denoted as *s*, the conditional probability of choices is represented as:


$$\left(Prob\left(j=best\;⋂k=worst\right)|s\right)=\frac{e^{\lambda_{js}-\lambda_{ks}}}{\sum_{l=\text{1}}^{J}\sum_{m=\text{1}}^{J}e^{\lambda_{ls}-\lambda_{ms}}-J}$$ (4)

where the $\;\lambda_{js}$ and $\lambda_{ks}\;$ parameters are class specific [72]. The probability of membership in these unobservable classes takes the multinomial logit form:


$$Prob(s)=\frac{e^{{(\theta }_{s}Z)}}{\sum_{s=\text{1}}^{S}e^{\theta_{s}Zk}}$$ (5)

where Z is a set of hypothesized drivers of class membership, the s^th^ parameter vector is normalized to zero for model identification, and θ_s_ characterizes the impact the drivers have on class membership [72]. Similar to the RPL model, the latent class preference shares were calculated using relation 3.

The LCM allows for estimation of individual participant’s probability of membership in each latent class. The difference between the highest probability class and the next highest probability class was calculated for each individual, and those that had a difference of at least 50% were assigned to their highest probability class. This method was developed by Bir et al. [73] and ensures that class characteristics are being analyzed over respondents who are solidly within a class. Demographics and work location status were statistically compared across the latent classes.

### Seemingly unrelated regression

Personal care questions were included in the survey to further analyze the potential of WFH preferences in response to saving time and money on grooming and hygiene practices. Respondents were asked if they were spending less on personal care (*LessPersonal*), spending less time on their appearance overall (*LessAppearance*), if they cared less about clothing (*LessClothes*), if they were washing their hair less (*LessWashHair*), and if they were showering less (*LessShower*) when compared to before March 2020. A seemingly unrelated regression was used to explore the relationship between the before mentioned behavioral changes, demographics, spending, and BWS class membership. The BWS class membership was included because several of the attributes were closely tied to personal hygiene and grooming such as saving money on lifestyle choices, such as eating lunches out and simplified or lessened grooming or dressing cost, in time and or/money, to work from home or remotely. A seemingly unrelated regression was used because there was likely a correlation in the error terms within the individual equations [74,75]. The model structure is defined as:


$$\begin{array}{cc} LessPersonal\;=\; & \beta_{\text{1}}Female+\beta_{\text{2}}Age+\beta_{\text{4}}Income+\beta_{\text{3}}Education+\beta_{\text{4}}HasKids+\beta_{\text{5}}WorkHome\\ & \;\;+\beta_{\text{6}}LHair+\beta_{\text{7}}LNail+\beta_{\text{8}}LMakeup+\beta_{\text{9}}LHairProduct+\beta_{\text{1}\text{0}}LSpa\\ & \;\;+\beta_{\text{1}\text{1}}ProbClass\text{1}+\beta_{\text{1}\text{2}}ProbClass\text{2}+\beta_{\text{1}\text{3}}ProbClass+\varepsilon \end{array}$$ (6)

$$\begin{array}{cc} LessAppearance\;=\; & \beta_{\text{1}\text{4}}Female+\beta_{\text{1}\text{5}}Age+\beta_{\text{1}\text{6}}Income+\beta_{\text{1}\text{7}}Education+\beta_{\text{1}\text{8}}HasKids\\ & \;\;+\beta_{\text{1}\text{9}}WorkHome+\beta_{\text{2}\text{0}}LHair+\beta_{\text{2}\text{1}}LNail+\beta_{\text{2}\text{2}}LMakeup+\beta_{\text{2}\text{3}}LHairProduct\\ & \;\;+\beta_{\text{2}\text{4}}LSpa+\beta_{\text{2}\text{5}}MCasual+\beta_{\text{2}\text{6}}LProfessional+\beta_{\text{2}\text{7}}ProbClass\text{1}\\ & \;\;+\beta_{\text{2}\text{8}}ProbClass\text{2}+\beta_{\text{2}\text{9}}ProbClass\text{3}+\varepsilon \; \end{array}$$ (7)

$$\begin{array}{cc} LessClothes\;=\; & \beta_{\text{3}\text{0}}Female+\beta_{\text{3}\text{1}}Age+\beta_{\text{3}\text{2}}Income+\beta_{\text{3}\text{3}}Education+\beta_{\text{3}\text{4}}HasKids\\ & \;\;+\beta_{\text{3}\text{5}}WorkHome+\beta_{\text{3}\text{6}}MCasual+\beta_{\text{3}\text{7}}LProfessional+\beta_{\text{3}\text{8}}ProbClass\text{1}\\ & \;\;+\beta_{\text{3}\text{9}}ProbClass\text{2}+\beta_{\text{4}\text{0}}ProbClass\text{3}+\varepsilon \; \end{array}$$ (8)

$$\begin{array}{cc} LessWashHair= & \;\beta_{\text{4}\text{1}}Female+\beta_{\text{4}\text{2}}Age+\beta_{\text{4}\text{3}}Income+\beta_{\text{4}\text{4}}Education+\beta_{\text{4}\text{5}}HasKids\\ & \;+\beta_{\text{4}\text{6}}WorkHome+\beta_{\text{4}\text{7}}LHair+\beta_{\text{4}\text{8}}LHairProduct+\beta_{\text{4}\text{9}}LAtHomeCare\\ & \;+\beta_{\text{5}\text{0}}ProbClass\text{1}+\beta_{\text{5}\text{1}}ProbClass\text{2}+\beta_{\text{5}\text{2}}ProbClass\text{3}+\varepsilon \end{array}$$ (9)

$$\begin{array}{cc} LessShower= & \;\beta_{\text{5}\text{3}}Female+\beta_{\text{5}\text{4}}Age+\beta_{\text{5}\text{5}}Income+\beta_{\text{5}\text{6}}Education+\beta_{\text{5}\text{7}}HasKids\\ & \;+\beta_{\text{5}\text{8}}WorkHome+\beta_{\text{5}\text{9}}LAtHomeCare+\beta_{\text{6}\text{0}}ProbClass\text{1}+\beta_{\text{6}\text{1}}ProbClass\text{2}\\ & \;+\beta_{\text{6}\text{2}}ProbClass\text{3}+\varepsilon \; \end{array}$$ (10)

Where *Female* indicates the respondent selected female, *Age* is the continuous variable for age, *Education* is the continuous variable for education, *HasKids* indicates the respondent has at least one child, and *WorkHome* indicates the respondent worked from home at least some of the time after COVID. *LHair* indicates the respondent spends less on hair services, *LNail*, indicates less spending on nail services, and *LMakeup* indicates less spending on makeup, *LHairProduct* indicates less spending on hair products, and *LSpa* indicates less spending on spa services. Spending less on at home personal care items is indicated by *LAtHomeCare*, *MCasual* indicates more spending on casual clothing, and *LProfessional* indicates less spending on professional clothing. *ProbClass1*, *ProbClass2*, and *ProbClass3* indicates the latent class the respondent had the highest probability of membership in for the best worst scaling model of the most and least important reasons for working remotely from home.

## Results and discussion

In total, 751 respondents completed the survey. The demographics of the survey respondents closely matched the US census with the exception of the age group 25–34 (14% in the sample, 18% in the census) (Table 2). The percentage of respondents with an income over $100,000 was slightly lower (23%) when compared to the US census (31%). For education, there was a lower percentage who did not graduate high school (4% vs 11%), and a higher percentage who attended college, associates or bachelor’s degree earned (32% vs 29%).

**Table 2:** Demographic information n = 751.

Demographic Variable	Percentage of Respondents N = 751	Employed N = 435	US Census
*Gender*			
Male	46	47	49
Female	54	53	51
*Age*			
18-24	13	14	12
25-34	14^ψ^	18	18
35-44	17	24	16
45-54	17	20	16
55-65	18	17	17
65 +	22	7	21
*Income*			
$0-$24,999	20	12	18
$25,000-$49,999	23	21	20
$50,000-$74,999	19	20	17
$75,000-$99,999	14	18	13
$100,000 and higher	23^ψ^	29	31
*Education*			
Did not graduate from high school	4^ψ^	3	11
Graduated from high school, Did not attend college	28	25	27
Attended college, No degree earned	22	20	21
Attended college, Associates or bachelor’s degree earned	32^ψ^	36	29
Attended college, Graduate or professional degree earned	14	16	13
*Region of residence*			
Northeast	18	20	17
South	37	37	38
Midwest	22	20	21
West	22	23	24
*Living Situation*			
Own a home	58		
Own an apartment	6		
Rent a house	10		
Rent an apartment	17		
Live with extended family members in the same house/apartment	11		
Live with unrelated individuals (roommates or shared living)	2		
None of the above	4		

ψIndicates the percentage of respondents is statistically different than the U.S. census at the 0.05 level.

To gauge how WFH, and COVID in general, impacted personal spending behavior, respondents were asked if they were spending more, less, or about the same for a series of products and services (Table 3). In general, respondents were spending less or about the same on items including professional and casual clothing, professional hair, nail, and spa services, eating in restaurants and travel. Less clear deviations from “about the same” were noted for to-go or on-the-go coffee or other beverages, hair care products, take-out food items, and work-related commuting expenses. Respondents spent about the same or more on at-home personal care items and personal protective equipment.

**Table 3:** Spending in Fall 2021 compared to pre-March 2020 (Pre-Covid) n = 751.

	Less	About the same	More	Not applicable/ Have never spent on this
Suits, dresses, or professional clothing	24	34	8	34
Casual Clothing	20	60	11	8
Professional hair services (i.e., color, cuts, professional styling)	24	44	8	24
Professional nail services	18	23	9	50
To-go or on-the-go coffee or other beverages	17	40	14	29
Makeup	19	36	10	35
Hair care products (i.e., shampoo/conditioner)	10	72	11	7
At-home personal care items (i.e., soaps or at-home treatments)	9	66	16	9
Professional spa or spa-like services	17	23	7	53
Personal Protective equipment (i.e., face coverings/masks)	13	40	35	12
Eating in restaurants	42	38	11	9
Take-out food items	19	47	23	12
Travel	36	31	10	22
Work-related commuting expenses	18	34	13	34

### Work from home

Four hundred and thirty-five respondents indicated they were employed at the time of the survey. Thirty eight percent of respondents said their job/employment could be done remotely, 23% said their job could be done remotely some of the time, and 38% said their job could not be done remotely. Thirty-two percent of respondents had a physical office but had the option to work remotely for the entire period studied. Between March 2020 and the survey launch date, 41% of respondents worked from home sometimes or always at some point. Thirty one percent worked from home sometimes or always during the entire time period and 40% never worked from home during the entire pre/post COVID period. Twenty percent of respondents had never worked from home before March 2020, but did work from home at least sometimes after March 2020.

Forty-one percent of respondents indicated working exclusively in an office or at a physical work site was their ideal work location. Thirty-four percent indicated working a hybrid option with both remote and on-site capabilities was ideal. Only 24% of respondents indicated working exclusively remote was their ideal work location. When asked if they would consider leaving their current position to obtain their preferred work environment, 38% chose yes, 28% chose maybe, 28% chose no, and 14% chose not applicable. Thirty seven percent of working respondents indicated they would take a pay cut to obtain flexibility in work location. The findings that relatively large portion of respondents are willing to leave their current positions (66%) or would take a pay cut (37%) for their preferred work environment might suggest that it would be helpful to delve into reasons behind their willingness to take such changes for preferred working environments.

### Best-worst scaling choice experiment results

#### RPL model.

Respondents were asked to complete a best-worst scaling choice experiment, indicating the most important and least important reasons for working remotely from home. Only respondents who were working at the time of the survey participated. First the MNL model was estimated. The MNL model had an AIC of 4.98. Due to expected heterogeneity amongst respondents the RPL model was estimated. The model was significant at the < 0.000 and had an AIC of 4.630 which indicated a better fit than the MNL model. Facilitating balance of work with caregiving responsibilities for children, family members, and/or pets (17%) was one of the largest preference shares (Table 4). Desire to reduce viral/disease exposure for self and/or household members (14%) was tied for 1^st^/2^nd^. Saving or reallocating commuting time and expense was tied for 2^nd^ (14%) with prefer the home environment (2^nd^/3^rd^, 13%). Concurrently the third top reasons were more productive at home (11%), and prefer the ability to intermingle work and home activities (12%). Tied for 4^th^ was saving money on lifestyle choices, such as eating lunches out (10%), and simplified or lessened grooming or dressing cost, in time and/or money, to work remotely (9%).

**Table 4:** RPL and Latent class results of best worst scaling experiment of the most and least important reasons for working remotely from home n = 435.

	RPL Model		Latent class model							
Attribute	Coefficient (SD)	Mean [confidence interval]	Coefficient				Preference Share			
			Class 1	Class 2	Class 3	Class 4	Class 1	Class 2	Class 3	Class 4
Saving money on lifestyle choices, such as eating lunches out	−0.190^***^ (0.385)	10%d [9-11]	0.614^***^	−0.847^***^	−1.564^***^	−0.011^***^	12%	4%	3%	12%
Saving or reallocating commuting time and expenses	0.137^***^ (0.389)	14%b [13-15]	0.844^***^	−0.211	−0.815^***^	0.155^**^	15%	7%	6%	14%
Desire to reduce viral/disease exposure for self and/or household members	0.161^***^ (0.465)	14%ab [13-15]	0.238^*^	0.122	0.027	0.147	8%	10%	13%	14%
Facilitating balance of work with caregiving responsibilities for children, family members, and/or pets	0.317^***^ (0.494)	17%a [15-18]	1.822^***^	−0.860^***^	1.263^***^	−0.060	41%	4%	46%	11%
More productive at home (i.e., less distractions)	−0.046 (0.395)	11%c [11-12]	−0.616^***^	1.289^***^	−0.312^**^	−0.080	4%	32%	9%	11%
Prefer the home environment	0.052 (0.260)	13%bc [12-13]	−0.712^***^	1.266^***^	−0.422^***^	0.088^*^	3%	31%	8%	13%
Simplified or lessened grooming or dressing cost, in time and/or money, to work from home or remotely	−0.270^***^ (0.391)	9%d [9-10]	0.416^***^	−0.663^***^	−2.114^***^	0.076	10%	4%	2%	13%
Prefer ability to intermingle work and home activities (i.e., waiting for plumber or electrician while conducting meetings or working from home simultaneously)	–	12%c [11-13]	–	–	–	–	7%	9%	13%	12%

***1% significance, **5% significance, *1% significance.

Each subscript letter denotes a preference share whose demographics do not differ significantly from the other preference shares at the.05 level.

#### Latent class model.

The latent class model allows for homogenous preferences within classes, and heterogenous preferences across classes of respondents allowing for a better understanding of the characteristics within groups of individuals with similar preferences. Four classes were deemed the most appropriate number based on BIC criteria as well as class size. Specifically, for the 2 class model the BIC was 14,724, for the 3 class model the BIC was 14,509, for the 4 class model the BIC was 14,361 and for the 5 class model the BIC was 14,373. Additionally, in the class 5 option one class only contained 2.7% of respondents, which coupled with the increase in BIC indicated class 4 was the most appropriate option. Class 1 had the highest preference shares for facilitating balance of work with caregiving responsibilities for children, family members, and/or pets (41%) as well as saving or reallocating commuting time and expenses (15%) (Table 4). Therefore, class 1 was deemed “balancing responsibilities and commuting time.” Class 2 was deemed “more productive and prefers home.” Class 2 had higher preference shares for more productive at home (32%) and prefer the home environment (31%). Class 3 was “balancing responsibilities, reduce exposure, and intermingling.” Like class 1, class 3 had a high preference share for facilitating balance of work with caregiving responsibilities for children, family members, and/or pets (46%), as well as desire to reduce viral/disease exposure for self and/or household members (13%), and prefer to intermingle work and home activities (i.e., waiting for plumber or electrician while conducting meetings or working from home simultaneously) (13%). Class 4 did not have clear preferences, and was named “everything or nothing.” It cannot be known if Class 4 believes that everything is equally important, or if they see everything as equally unimportant.

Classes 1–3 suggest that people are trying to balance caretaking of children, family members, and pets, as well as to accomplish tasks for their homes and home lives that are not easily accomplished outside of usual business hours. For these types of tasks, it may be that weekday or business day scheduling is easier and less costly than attempting home care or repair appointments in the evenings or on weekends. Or, it may also be the case that time savings from commutes facilitates scheduling, even if appointments or needs are not competing directly during business hours. Likely there are a variety of motivations including both time saved by not commuting and facilitating business hour availability for personal needs, among other potential reasons.

Following the methodology outlined by Bir et al. [73] the demographics of the individual classes were analyzed (Table 5). The LCM demographics in Table 5 could provide useful insights into what roles and tasks individual demographic groups may need to fulfil at home. The insights would allow employers to better understand potential motivations behind the WFH preference of different demographic groups helping develop tailored working modals among employees rather than requiring RTO/back-to-office modalities universally to all employees. Class 4 had a higher percentage of males (58%), when compared to all other classes. Alternatively, classes 1–3, had higher proportions of women around 70%. Considering that class 1 and 3 had higher preference shares for balancing family care responsibilities and house related activities, the LCM demographics suggest that more female respondents continued to try to manage responsibilities of caregiving for their children, family members, and pets than male respondents [76].

**Table 5:** LCM demographics. Respondents assigned to highest probability class, only respondents with a 50% difference in highest and next highest probability.

	Class 1 n = 64	Class 2 n = 43	Class 3 n = 52	Class 4 n = 244
**Demographic Variable**				
*Gender*				
Male	31a^1^	33a	27a	58b
Female	69a	67a	73a	42b
*Age*				
18-24	13ab	7ab	6a	17b
25-34	13ab	2a	15ab	24b
35-44	23a	21a	31a	24a
45-54	25ab	28a	27ab	16b
55-65	17	33	15	14
65 +	9ac	9ab	6c	5c
*Income*				
$0-$24,999	12a	14a	12a	12a
$25,000-$49,999	27ab	30a	19ab	17b
$50,000-$74,999	16a	21a	27a	19a
$75,000-$99,999	12a	19a	15a	22a
$100,000 and higher	33a	16a	27a	30a
*Education*				
Did not graduate from high school	5a	5a	4a	3a
Graduated from high school, Did not attend college	16a	23ab	23ab	28b
Attended college, No degree earned	28a	25ab	23ab	16b
Attended college, Associates or bachelor’s degree earned	34a	35a	40a	36a
Attended college, Graduate or professional degree earned	17a	12a	10a	17a
*Region of residence*				
Northeast	24a	21a	13a	19a
South	34ab	44ab	50a	33b
Midwest	23a	16a	12a	21a
West	20a	19a	25a	26a
*Work from home*				
Worked from home sometimes or always during period studied	19a	40bc	21ab	36c
Never worked from home during period studied	59a	30b	48ab	34b
Job can be remote	42a	65b	56ab	68b
Would leave their current job to be remote	27ab	21a	40b	30ab
Would take a pay cut to be remote	20a	37ab	31ab	45b

^1^The percentage of respondents in a particular demographic category were statistically compared across classes. Matching letters indicate there is no statistical difference, differing letters indicates there is a statistical difference. For example, the percentage of women is not statistically different for the first 3 classes but is for class 4.

Across age categories studied, age groups of 35–44 and 45–54 show higher percentage of membership for class 1 and 3 which have higher preference shares for balancing responsibility related to family members and/or housework. Considering these age groups are often managing children and potentially caretaking other family members, it is unsurprising that Class 2 had a higher percentage of respondents aged 45–54 and 55–65 preferring WFH to facilitate the balancing of responsibilities. Class 3 had a higher percentage of respondents aged 25–34 when compared to class 2. Finally, class 4 had a higher percentage of respondents aged 18–24 which suggests that this young age group may have relatively less caregiving responsibility or differing reasons for their preferred working location than the older respondents.

For income, the only statistical difference was class 2 having a higher percentage of respondents in the income bracket $25,000-$49,000 when compared to class 4. Considering education level, class 4 had a higher percentage of respondents who selected graduated from high school, did not attend college when compared to class 1. Class 1 also had a higher percentage of respondents who selected attended college, no degree earned when compared to class 4. A higher percentage of respondents from the south were in class 3, when compared to class 4.

Considering work from home status, a higher percentage of respondents worked from home sometimes in class 2 when compared to classes 1 and 3. This suggests that people in class 2 may prefer WFH because they are more productive or otherwise better off based on their own past experiences of WFH. Class 1 and class 4 had more respondents who have never worked from home and those respondents answered balancing responsibility as an important reason for desired flexibility. Experience level with WFH arrangement is unsurprisingly impactful, at least in part, on individual’s preferences for WFH. Moving forward, as more individuals may have lived experiences of both WFH and in-office work, it would be expected that learned experiences continue to shape even more strongly individual’s preferences for work locations. At the start of the pandemic, relatively few people had lived experience of working outside of the office compared to during or after the pandemic period. These experiences and having worked in various places and with varying levels of flexibility can be assumed to shape future opinions.

More people in classes 2 (65%) and 4 (68%) had a job that could be remote when compared to class 1 (42%). This further reflects the findings regarding work from home status as it is likely having a job that can be remote and WFH during the COVID period are highly correlated. A higher percentage of respondents in class 3 (40%) were willing to leave a job to be remote when compared to group 2 (21%). Class 3 was mostly defined by a large preference share for facilitating a balance of work with caregiving responsibilities. Being willing to leave a job for WFH when there may not be alternative caregiving options makes since given that groups preferences. A higher percentage of those in group 4 (45%) were willing to take a pay cut to WFH when compared to class 1. Class 4’s preferences between the attributes studied were not strong, potentially pointing towards multiple concerns which would facilitate a strong enough WFH preference to be willing to take a pay cut.

### Seemingly unrelated regression results

Respondents were asked about behavioral changes including spending changes since March of 2020 (Table 6). Twenty-three percent of respondents reported that they spent less time on personal care than pre-March 2020. For caring about their appearance overall when compared to pre-March, 25% of respondents indicated they cared less. Twenty-four percent cared less about what clothes they wear when compared to pre-March. When asked if they wash their hair less often, 19% selected yes, and 16% indicated they showered less.

**Table 6:** Seemingly unrelated regression of personal hygiene behavior changes since March 2020. N = 435.

	Behavior changes since March 2020 Coefficient (Standard Error)				
Independent variable	Spends less time on personal care	Spends less time on appearance overall	Cares less about clothes	Washes hair less	Showers less
Female	−0.061 (0.042)	−0.039 (0.042)	−0.005 (0.043)	−0.030 (0.039)	−0.056 (0.036)
Age	0.001 (0.015)	−0.008 (0.016)	0.010 (0.016)	−0.015 (0.014)	−0.019 (0.013)
Income	0.002 (0.016)	−0.023 (0.016)	−0.018 (0.016)	0.022 (0.015)	0.001 (0.014)
Education	−0.008 (0.020)	−0.022 (0.020)	−0.044^**^ (0.020)	−0.018 (0.018)	−0.007 (0.017)
Has children	0.143^**^ (0.046)	0.100^**^ (0.046)	0.140^**^ (0.047)	0.143^***^ (0.043)	0.118^**^ (0.040)
Worked from home at least some of the time after COVID	0.098^**^ (0.045)	0.154^***^ (0.045)	0.186^***^ (0.046)	0.014 (0.042)	0.139^***^ (0.038)
Spends less on hair services^1^	−0.010 (0.047)	−0.015 (0.046)		−0.009 (0.038)	
Spends less on nail services^1^	0.056 (0.052)	0.022 (0.051)			
Spends less on makeup^1^	0.092^*^ (0.049)	0.117^**^ (0.048)			
Spends less on hair products^1^	0.193^**^ (0.064)	0.134^**^ (0.062)		0.148^**^ (0.057)	
Spends less on spa services^1^	−0.020 (0.051)	−0.017 (0.050)			
Spends less on at-home personal care items				0.055 (0.058)	0.067 (0.049)
Spends more on casual clothing^1^		0.080^*^ (0.047)	−0.019 (0.049)		
Spends less on professional clothes^1^		0.002 (0.042)	0.071^*^ (0.039)		
First Class 1^2^	−0.019 (0.060)	0.013 (0.060)	−0.036 (0.061)	−0.090 (0.056)	−0.020 (0.052)
First Class 2^2^	−0.099 (0.071)	−0.086 (0.071)	−0.144^**^ (0.073)	−0.012 (0.067)	−0.112 (0.062)
First Class 3^2^	−0.019 (0.066)	−0.027 (0.066)	−0.017 (0.066)	0.013 (0.061)	−0.028^*^ (0.056)
Constant	0.170^*^ (0.096)	0.299^**^ (0.095)	0.271^**^ (0.097)	0.202^**^ (0.089)	0.182^**^ (0.082)

^1^ Spending compared to pre-March 2020.

^2^ Respondent’s BWS class assignment as described in Table X.

Statistically significant at the *0.01 level, **0.05 level, *** < 0.001.

A seemingly unrelated regression was used to evaluate relationships between demographics, spending changes, best worst scaling class membership and the aforementioned behavioral changes. All estimations were statistically significant (<0.000), spends less time on person care had an r-squared of 0.1254, spends less time on appearance overall had an r-squared of 0.1227, cares less about clothes had an r-squared of 0.0831, washes hair less had an r-squared of 0.0995, and showers less had an r-squared of 0.1164. Gender, age, and income were not statically significant in any of the personal hygiene change models. As education increased, respondents cared more about clothing (−0.044). Having children increased the likelihood that respondents spent less time on personal care (0.143), spent less time on appearance overall (0.100), cared less about clothes (0.140), washed hair less (0.143), and showered less (0.118). This suggests that respondents with children probably cared relatively more about balancing responsibility for their children over personal care while they work from home. WFH at least some of the time after COVID-19 increased the likelihood for all behavioral changes with the exception of washing hair less.

Spending less on hair, nail, spa services, and other at-home personal care items were not statistically significant in any of the behavioral change models. Spending less on makeup increased the likelihood that the respondent spends less time on personal care (0.092) and spends less time on appearance overall (0.117). Spending less on hair products increased the likelihood the respondent spends less time on personal care (0.193), less time on appearance overall (0.134), and washes hair less (0.148). Spending more on casual clothing increased the likelihood the respondent spends less time on appearance overall (0.080). Spending less on professional clothing increased the likelihood the respondent cared less about clothes (0.071).

For the best worst scaling latent class model, respondents were assigned to 4 classes based on their probability of membership. Being a member of class 1 (balancing responsibilities and commuting time) did not increase the likelihood of any of the behavioral changes. Being a member of class 2 (more productive and prefers home) decreased the probability the respondent cared less about clothing. It is possible that members of class 2 had always cared less about clothing or had already streamlined their wardrobes. Or, it is plausible that behavioral changes and lifestyle changes in the 2020–2021 time period are already reflected in this class membership. Being a member of class 3 (balancing responsibilities, reduce exposure, and intermingling) decreased the likelihood the respondent showered less. Potentially, given this class (class 3) was concerned about reduced exposure and responsibility, they may continue to be more regimented with personal hygiene overall.

### Implications and suggestions

The findings indicate that preferences for WFH are driven by the need to manage competing demands between work and household responsibilities. The most salient motivations such as caregiving, commuting time savings, and/or health risk reduction suggest that WFH enables the joint production of work and home activities. This perspective reframes WFH as a structural component of time allocation rather than a temporary or discretionary perk. In addition, a willingness to leave their jobs or accept lower wages in exchange for flexibility indicates that WFH operates as a valued non-wage job attribute that enters directly into worker utility.

A key contribution of the latent class results is the identification of meaningful heterogeneity in WFH preferences. Workers differ not only in the intensity of their preferences but also in the underlying motivations which range from caregiving and commuting considerations to productivity gains and home environment preferences. The heterogeneity implies that uniform RTO mandates might not be efficient, as they ignore differences in both constraints and productivity across workers. From a firm perspective, rigid policies may lead to mismatch, reduced job satisfaction, and higher turnover. Instead, firms may benefit from offering a menu of work arrangements, fully remote, hybrid, and fully on-site, allowing workers to self-select into roles that best match their productivity profiles and personal circumstances. This type of screening mechanism can improve both worker welfare and firm performance by aligning work arrangements with underlying heterogeneity.

The results also highlight important distributional implications. Caregiving-related motivations are particularly pronounced among certain demographic groups, suggesting that WFH can support labor force attachment for workers facing substantial household responsibilities. In this sense, working remotely may function as a partial substitute for some aspects of formal childcare and related infrastructure. Restricting access to WFH could therefore disproportionately affect caregivers and potentially reduce labor supply, while expanding flexibility may improve retention and participation.

Finally, the importance of commuting costs and health considerations highlights that work location is not merely logistical but affects both worker productivity and firm organization. These factors suggest that WFH should be treated as a multi-dimensional job attribute shaped by productivity, household constraints, and risk preferences.

## Conclusion

As WFH arrangements became increasingly common during and following the COVID-19 pandemic, public debates and employer announcements about policy changes have focused primarily on implications for productivity. However, the underlying motivations for employees’ preferences toward remote or hybrid work have received comparatively limited attention. This study was motivated to better understand preferences surrounding workplace flexibility or WFH arrangements in order to inform the development of more responsive and sustainable workplace policies.

Using a best-worst scaling experiment and latent class modeling, this research identified the most and least important factors influencing WFH preferences. Balancing work with caregiving responsibilities emerged as the most valued reason for WFH flexibility, followed by saving commuting time and costs, limiting exposure to illness, and preference for the home environment. The identified heterogeneity in worker preferences suggests that rigid RTO mandates may inadvertently harm productivity by disrupting the specific work-life balance mechanisms workers have established. Rather than a “one-size-fits-all” approach, organizations should implement flexible policies that allow different segments – such as those driven by caregiving or those focused on environmental comfort – to optimize their specific work conditions, thereby fostering higher engagement and output.

In addition, although some attributes studied including limiting exposure to illness may be less important in non-pandemic times, many of the other attributes are still very relevant. Additionally, the potential option and normalization of WFH is still impacting decision making during periods of illness such as “flu season.” Making concrete recommendations for workplaces is beyond the scope of this work. However, the findings can open a dialogue towards improving worker satisfaction and retention. For example, facilitating balance of work with caregiving responsibilities for children, family members, and/or pets was a top reason for a WFH preference. If WFH is not possible full time, perhaps allowing partial WFH days- for example surrounding the end of the school day for children, extended lunch breaks for letting pets out during the lunch hour or other creative arrangements can be evaluated. Although the pandemic is no longer top of mind, having flexibility to WFH when ill with say a cough or flu may decrease the number of sick days, and stop the spread of illness throughout the workplace, which was still a concern for respondents.

The latent class analysis revealed four distinct classes of respondents, reflecting heterogeneous preferences associated with demographic and experiential characteristics. For example, female respondents were represented in classes that prioritized caregiving and commuting-related concerns, whereas younger respondents (aged 18–24) were more likely to belong to the class with diffuse or undifferentiated preferences. Middle-aged respondents, particularly those aged 35–54, tended to fall into segments with strong preferences for balancing caregiving and home-related responsibilities.

The seemingly unrelated regression analysis further supported these findings, indicating that respondents with children were significantly more likely to report reduced time and effort devoted to personal care and grooming. Respondents who worked from home at least some of the time after COVID were more likely to report behavioral adaptations, such as changes in grooming and appearance routines. These results underscore the influence of personal and household characteristics on the perceived value and consequences of WFH.

Taken together, the findings highlight the importance of moving beyond generalized assessments of productivity when evaluating remote or hybrid work policies because productivity is not a static metric but might be affected by an individual’s work-life balance. A deeper understanding of the motivations underlying the identified employee preferences particularly those rooted in caregiving responsibilities and time management can contribute to more constructive and targeted policy discussions. Employers seeking to design effective and equitable workplace arrangements should consider tailored flexible work policies that align with the specific drivers of their workforce, such as prioritizing flexibility for those with heavy caregiving burdens or long commutes, aligning organizational objectives with employee well-being and operational efficiency. This research highlights the balancing act faced by some workers and employers who are trying to retain top talent may consider flexible options that include some WFH capabilities to better align with employee needs.

## Supporting information

S1 File Survey questionnaire used in the study, including all structured and dropdown-based questions administered to participants. (DOCX)

S2 File Dataset containing the compiled responses and supplementary variables used for analysis. (XLSX)

S3 File Completed checklist for human subjects research compliance, as required by the journal. (DOCX)
