# Supplementary material for: Understanding why people value working from home or hybrid workplace flexibility a study of preferences during the COVID-19 pandemic
Source: PLoS One. 2026 May 4;21(5):e0348206. doi: 10.1371/journal.pone.0348206 (PMC13138752; doi:10.1371/journal.pone.0348206)
Supplement: S1 File — (DOCX) [file pone.0348206.s001.docx]

Q1.1 Browser Meta Info

Browser (1)

Version (2)

Operating System (3)

Screen Resolution (4)

Flash Version (5)

Java Support (6)

User Agent (7)

Q1.2 Timing

First Click (1)

Last Click (2)

Page Submit (3)

Click Count (4)

Q1.3 **RESEARCH PARTICIPANT CONSENT FORM** **Fall 2021 Home & Work Lifestyle Preferences** **Dr. Nicole Widmar, Dr. Courtney Bir, and Mario Ortez** **Agricultural Economics** **Purdue University** **IRB Number: IRB-2021-1715**   **Key Information** Please take the time to review this information carefully. This is a research study. Your participation in this study is voluntary which means that you may choose not to participate at any time without penalty or loss of benefits to which you are otherwise entitled. You may ask questions to the researchers about the study whenever you would like. If you decide to take part in the study, be sure you understand what you will do and any possible risks or benefits.    **Thank you for participating in this study. The following contains information about the study and your rights as a research participant.**   This is a web-based survey research study designed to elicit your opinions about home and work lifestyle preferences in the Fall of 2021. Proceeding with the web-based survey will imply your consent to participate in this study. There are about 30 questions asking about your interests and experience in working outside the home versus from home, in addition to questions about pets and other lifestyle preferences or choices.    **What is the purpose of this study?** The purpose of this study is to understand how individuals with different experiences or situations over the past 18 months, during which the U.S. has experienced COVID-19 related disruptions, prefer to work, live, and allocate their time. We would like to enroll about 800 respondents in this study.   **What will I do if I choose to be in this study?** You will be asked questions about your demographics, individual and/or household hobbies and preferences, and about your own desires to work from home versus outside the home. Additionally, you will be presented with multiple hypothetical scenarios asking how you would prioritize or allocate your own time. If you choose not to answer a specific question(s), you may skip that question and move forward at any time.   **How long will I be in the study?** The approximate total time commitment for this study is approximately 25 minutes. There will be approximately 30 questions.   **What are the possible risks or discomforts?** The risks associated with this study are minimal. The risks are not greater than those ordinarily encountered in daily life. Moreover, you may stop the survey at any time. By participating in the study, you are at no greater cost than you would encounter in daily life. Breach of confidentiality is always a risk with data, but we will take precautions to minimize this risk as described in the confidentiality section.   **Are there any potential benefits?** This research will assist researchers in better understanding U.S. residents' comfort levels with various activities in the Fall of 2021, as well as gain insight into how time is valued for various uses.   **Are there costs to me for participation?** There are no anticipated costs to participate in this research.   **Will information about me and my participation be kept confidential?** At no point will a data file be constructed in which your name is linked with your responses. The data will be stored by the principal investigators with no intention to destroy the data. The data will only be released in summaries in which no individual’s answers can be identified. The project's research records may be reviewed by departments at Purdue University responsible for regulatory and research oversight. Breach of confidentiality is always a risk with data, but we will take precautions to minimize this risk.   **What are my rights if I take part in this study?** You do not have to participate in this research project. If you agree to participate, you may withdraw your participation at any time without penalty or skip questions if necessary. If you wish to comment on any questions, please feel free to use the text box provided at the end of the survey.   **Who can I contact if I have questions about the study?** If you have any questions, comments, or concerns regarding this survey, please contact the principal investigators using this contact information: • Dr. Nicole Widmar at Purdue University by phone: (765) 494-2567 or email: nwidmar@purdue.edu(initial point of contact)  • Dr. Courtney Bir at Oklahoma State University by email: courtney.bir@okstate.edu 
• Mario Ortez at Purdue University by email: mortez@purdue.edu
 **To report anonymously via Purdue’s Hotline see www.purdue.edu/hotline**   If you have questions about your rights while taking part in the study or have concerns about the treatment of research participants, please call the Human Research Protection Program at (765) 494-5942, email (irb@purdue.edu), or write to: Human Research Protection Program - Purdue University Ernest C. Young Hall, Room 1032 155 S. Grant St. West Lafayette, IN 47907-2114   Documentation of Informed Consent I have had the opportunity to read this consent form and have the research study explained. I have had the opportunity to ask questions about the research study, and my questions have been answered. I am prepared to participate in the research study described above. I can print a copy of this consent form for my records. By clicking the arrow below, I give my consent to participate in this study.  

|  |  |
| --- | --- |

Q1.4 I am:

- Male (1)
- Female (2)

|  |
| --- |

Q1.5 I am _____ years old.

- Under 18 (1)
- 18 - 24 (2)
- 25 - 34 (3)
- 35 - 44 (4)
- 45 - 54 (5)
- 55 - 64 (6)
- 65 + (7)

Q1.6 My household (including myself, all other adults and any children) has the following number of members. (Please enter a "0" for children if your household does not contain any members under 18 years of age.)

- Adults (18 years and older) (1)
- Children, Aged 12 - 18 years (2)
- Children, Aged 5 to 11 years (3)
- Children, Under 5 years of age (4)

|  |
| --- |

Q1.7 My annual pre-tax household income is:

- $0-$24,999 (1)
- $25,000-$49,999 (2)
- $50,000-$74,999 (3)
- $75,000-$99,999 (4)
- $100,000 and higher (5)

|  |
| --- |

Q1.8 The best description of my educational background is:

- Did not graduate from high school (1)
- Graduated from high school, Did not attend college (2)
- Attended College, No Degree earned (3)
- Attended College, Associate's or Bachelor's Degree earned (4)
- Attended College, Graduate or Professional Degree earned (5)

|  |
| --- |

Q1.9 My region of residence is: ___________. Select one option from the drop down menu.

- Northeast (CT, ME, MA, NH, NJ, NY, PA, RI, VT) (1)
- South (AL, AR, DE, DC, FL, GA, KY, LA, MD, MS, NC, OK, SC, TN, TX, VA, WV) (2)
- Midwest (IL, IN, IA, KS, MI, MN, MO, NE, ND, OH, SD, WI) (3)
- West (AK, AZ, CA, CO, HI, ID, MT, NV, NM, OR, UT, WA, WY) (4)

Display This Question:

If My region of residence is: ___________. Select one option from the drop down menu. = Northeast (CT, ME, MA, NH, NJ, NY, PA, RI, VT)

Q1.10 My state and county of residence in the Northeast is:

State (1)

County (2)

- Connecticut (1)
- Connecticut ~ Fairfield County (2)
- Connecticut ~ Hartford County (3)
- Connecticut ~ Litchfield County (4)
- Connecticut ~ Middlesex County (5)
- Connecticut ~ New Haven County (6)
- Connecticut ~ New London County (7)
- Connecticut ~ Tolland County (8)
- Connecticut ~ Windham County (9)
- Maine (10)
- Maine ~ Androscoggin County (11)
- Maine ~ Aroostook County (12)
- Maine ~ Cumberland County (13)
- Maine ~ Franklin County (14)
- Maine ~ Hancock County (15)
- Maine ~ Kennebec County (16)
- Maine ~ Knox County (17)
- Maine ~ Lincoln County (18)
- Maine ~ Oxford County (19)
- Maine ~ Penobscot County (20)
- Maine ~ Piscataquis County (21)
- Maine ~ Sagadahoc County (22)
- Maine ~ Somerset County (23)
- Maine ~ Waldo County (24)
- Maine ~ Washington County (25)
- Maine ~ York County (26)
- Massachusetts (27)
- Massachusetts ~ Barnstable County (28)
- Massachusetts ~ Berkshire County (29)
- Massachusetts ~ Bristol County (30)
- Massachusetts ~ Dukes County (31)
- Massachusetts ~ Essex County (32)
- Massachusetts ~ Franklin County (33)
- Massachusetts ~ Hampden County (34)
- Massachusetts ~ Hampshire County (35)
- Massachusetts ~ Middlesex County (36)
- Massachusetts ~ Town and County of Nantucket (37)
- Massachusetts ~ Norfolk County (38)
- Massachusetts ~ Plymouth County (39)
- Massachusetts ~ Suffolk County (40)
- Massachusetts ~ Worcester County (41)
- New Hampshire (42)
- New Hampshire ~ Belknap County (43)
- New Hampshire ~ Carroll County (44)
- New Hampshire ~ Cheshire County (45)
- New Hampshire ~ Coos County (46)
- New Hampshire ~ Grafton County (47)
- New Hampshire ~ Hillsborough County (48)
- New Hampshire ~ Merrimack County (49)
- New Hampshire ~ Rockingham County (50)
- New Hampshire ~ Strafford County (51)
- New Hampshire ~ Sullivan County (52)
- New Jersey (53)
- New Jersey ~ Atlantic County (54)
- New Jersey ~ Bergen County (55)
- New Jersey ~ Burlington County (56)
- New Jersey ~ Camden County (57)
- New Jersey ~ Cape May County (58)
- New Jersey ~ Cumberland County (59)
- New Jersey ~ Essex County (60)
- New Jersey ~ Gloucester County (61)
- New Jersey ~ Hudson County (62)
- New Jersey ~ Hunterdon County (63)
- New Jersey ~ Mercer County (64)
- New Jersey ~ Middlesex County (65)
- New Jersey ~ Monmouth County (66)
- New Jersey ~ Morris County (67)
- New Jersey ~ Ocean County (68)
- New Jersey ~ Passaic County (69)
- New Jersey ~ Salem County (70)
- New Jersey ~ Somerset County (71)
- New Jersey ~ Sussex County (72)
- New Jersey ~ Union County (73)
- New Jersey ~ Warren County (74)
- New York (75)
- New York ~ Albany County (76)
- New York ~ Allegany County (77)
- New York ~ Bronx County (78)
- New York ~ Broome County (79)
- New York ~ Cattaraugus County (80)
- New York ~ Cayuga County (81)
- New York ~ Chautauqua County (82)
- New York ~ Chemung County (83)
- New York ~ Chenango County (84)
- New York ~ Clinton County (85)
- New York ~ Columbia County (86)
- New York ~ Cortland County (87)
- New York ~ Delaware County (88)
- New York ~ Dutchess County (89)
- New York ~ Erie County (90)
- New York ~ Essex County (91)
- New York ~ Franklin County (92)
- New York ~ Fulton County (93)
- New York ~ Genesee County (94)
- New York ~ Greene County (95)
- New York ~ Hamilton County (96)
- New York ~ Herkimer County (97)
- New York ~ Jefferson County (98)
- New York ~ Kings County (99)
- New York ~ Lewis County (100)
- New York ~ Livingston County (101)
- New York ~ Madison County (102)
- New York ~ Monroe County (103)
- New York ~ Montgomery County (104)
- New York ~ Nassau County (105)
- New York ~ New York County (106)
- New York ~ Niagara County (107)
- New York ~ Oneida County (108)
- New York ~ Onondaga County (109)
- New York ~ Ontario County (110)
- New York ~ Orange County (111)
- New York ~ Orleans County (112)
- New York ~ Oswego County (113)
- New York ~ Otsego County (114)
- New York ~ Putnam County (115)
- New York ~ Queens County (116)
- New York ~ Rensselaer County (117)
- New York ~ Richmond County (118)
- New York ~ Rockland County (119)
- New York ~ St. Lawrence County (120)
- New York ~ Saratoga County (121)
- New York ~ Schenectady County (122)
- New York ~ Schoharie County (123)
- New York ~ Schuyler County (124)
- New York ~ Seneca County (125)
- New York ~ Steuben County (126)
- New York ~ Suffolk County (127)
- New York ~ Sullivan County (128)
- New York ~ Tioga County (129)
- New York ~ Tompkins County (130)
- New York ~ Ulster County (131)
- New York ~ Warren County (132)
- New York ~ Washington County (133)
- New York ~ Wayne County (134)
- New York ~ Westchester County (135)
- New York ~ Wyoming County (136)
- New York ~ Yates County (137)
- Pennsylvania (138)
- Pennsylvania ~ Adams County (139)
- Pennsylvania ~ Allegheny County (140)
- Pennsylvania ~ Armstrong County (141)
- Pennsylvania ~ Beaver County (142)
- Pennsylvania ~ Bedford County (143)
- Pennsylvania ~ Berks County (144)
- Pennsylvania ~ Blair County (145)
- Pennsylvania ~ Bradford County (146)
- Pennsylvania ~ Bucks County (147)
- Pennsylvania ~ Butler County (148)
- Pennsylvania ~ Cambria County (149)
- Pennsylvania ~ Cameron County (150)
- Pennsylvania ~ Carbon County (151)
- Pennsylvania ~ Centre County (152)
- Pennsylvania ~ Chester County (153)
- Pennsylvania ~ Clarion County (154)
- Pennsylvania ~ Clearfield County (155)
- Pennsylvania ~ Clinton County (156)
- Pennsylvania ~ Columbia County (157)
- Pennsylvania ~ Crawford County (158)
- Pennsylvania ~ Cumberland County (159)
- Pennsylvania ~ Dauphin County (160)
- Pennsylvania ~ Delaware County (161)
- Pennsylvania ~ Elk County (162)
- Pennsylvania ~ Erie County (163)
- Pennsylvania ~ Fayette County (164)
- Pennsylvania ~ Forest County (165)
- Pennsylvania ~ Franklin County (166)
- Pennsylvania ~ Fulton County (167)
- Pennsylvania ~ Greene County (168)
- Pennsylvania ~ Huntingdon County (169)
- Pennsylvania ~ Indiana County (170)
- Pennsylvania ~ Jefferson County (171)
- Pennsylvania ~ Juniata County (172)
- Pennsylvania ~ Lackawanna County (173)
- Pennsylvania ~ Lancaster County (174)
- Pennsylvania ~ Lawrence County (175)
- Pennsylvania ~ Lebanon County (176)
- Pennsylvania ~ Lehigh County (177)
- Pennsylvania ~ Luzerne County (178)
- Pennsylvania ~ Lycoming County (179)
- Pennsylvania ~ McKean County (180)
- Pennsylvania ~ Mercer County (181)
- Pennsylvania ~ Mifflin County (182)
- Pennsylvania ~ Monroe County (183)
- Pennsylvania ~ Montgomery County (184)
- Pennsylvania ~ Montour County (185)
- Pennsylvania ~ Northampton County (186)
- Pennsylvania ~ Northumberland County (187)
- Pennsylvania ~ Perry County (188)
- Pennsylvania ~ Philadelphia County (189)
- Pennsylvania ~ Pike County (190)
- Pennsylvania ~ Potter County (191)
- Pennsylvania ~ Schuylkill County (192)
- Pennsylvania ~ Snyder County (193)
- Pennsylvania ~ Somerset County (194)
- Pennsylvania ~ Sullivan County (195)
- Pennsylvania ~ Susquehanna County (196)
- Pennsylvania ~ Tioga County (197)
- Pennsylvania ~ Union County (198)
- Pennsylvania ~ Venango County (199)
- Pennsylvania ~ Warren County (200)
- Pennsylvania ~ Washington County (201)
- Pennsylvania ~ Wayne County (202)
- Pennsylvania ~ Westmoreland County (203)
- Pennsylvania ~ Wyoming County (204)
- Pennsylvania ~ York County (205)
- Rhode Island (206)
- Rhode Island ~ Bristol County (207)
- Rhode Island ~ Kent County (208)
- Rhode Island ~ Newport County (209)
- Rhode Island ~ Providence County (210)
- Rhode Island ~ Washington County (211)
- Vermont (212)
- Vermont ~ Addison County (213)
- Vermont ~ Bennington County (214)
- Vermont ~ Caledonia County (215)
- Vermont ~ Chittenden County (216)
- Vermont ~ Essex County (217)
- Vermont ~ Franklin County (218)
- Vermont ~ Grand Isle County (219)
- Vermont ~ Lamoille County (220)
- Vermont ~ Orange County (221)
- Vermont ~ Orleans County (222)
- Vermont ~ Rutland County (223)
- Vermont ~ Washington County (224)
- Vermont ~ Windham County (225)
- Vermont ~ Windsor County (226)

Display This Question:

If My region of residence is: ___________. Select one option from the drop down menu. = South (AL, AR, DE, DC, FL, GA, KY, LA, MD, MS, NC, OK, SC, TN, TX, VA, WV)

Q1.11 My state and county of residence in the South is:

State (1)

County (2)

- Alabama (1)
- Alabama ~ Autauga County (2)
- Alabama ~ Baldwin County (3)
- Alabama ~ Barbour County (4)
- Alabama ~ Bibb County (5)
- Alabama ~ Blount County (6)
- Alabama ~ Bullock County (7)
- Alabama ~ Butler County (8)
- Alabama ~ Calhoun County (9)
- Alabama ~ Chambers County (10)
- Alabama ~ Cherokee County (11)
- Alabama ~ Chilton County (12)
- Alabama ~ Choctaw County (13)
- Alabama ~ Clarke County (14)
- Alabama ~ Clay County (15)
- Alabama ~ Cleburne County (16)
- Alabama ~ Coffee County (17)
- Alabama ~ Colbert County (18)
- Alabama ~ Conecuh County (19)
- Alabama ~ Coosa County (20)
- Alabama ~ Covington County (21)
- Alabama ~ Crenshaw County (22)
- Alabama ~ Cullman County (23)
- Alabama ~ Dale County (24)
- Alabama ~ Dallas County (25)
- Alabama ~ DeKalb County (26)
- Alabama ~ Elmore County (27)
- Alabama ~ Escambia County (28)
- Alabama ~ Etowah County (29)
- Alabama ~ Fayette County (30)
- Alabama ~ Franklin County (31)
- Alabama ~ Geneva County (32)
- Alabama ~ Greene County (33)
- Alabama ~ Hale County (34)
- Alabama ~ Henry County (35)
- Alabama ~ Houston County (36)
- Alabama ~ Jackson County (37)
- Alabama ~ Jefferson County (38)
- Alabama ~ Lamar County (39)
- Alabama ~ Lauderdale County (40)
- Alabama ~ Lawrence County (41)
- Alabama ~ Lee County (42)
- Alabama ~ Limestone County (43)
- Alabama ~ Lowndes County (44)
- Alabama ~ Macon County (45)
- Alabama ~ Madison County (46)
- Alabama ~ Marengo County (47)
- Alabama ~ Marion County (48)
- Alabama ~ Marshall County (49)
- Alabama ~ Mobile County (50)
- Alabama ~ Monroe County (51)
- Alabama ~ Montgomery County (52)
- Alabama ~ Morgan County (53)
- Alabama ~ Perry County (54)
- Alabama ~ Pickens County (55)
- Alabama ~ Pike County (56)
- Alabama ~ Randolph County (57)
- Alabama ~ Russell County (58)
- Alabama ~ St. Clair County (59)
- Alabama ~ Shelby County (60)
- Alabama ~ Sumter County (61)
- Alabama ~ Talladega County (62)
- Alabama ~ Tallapoosa County (63)
- Alabama ~ Tuscaloosa County (64)
- Alabama ~ Walker County (65)
- Alabama ~ Washington County (66)
- Alabama ~ Wilcox County (67)
- Alabama ~ Winston County (68)
- Arkansas (69)
- Arkansas ~ Arkansas County (70)
- Arkansas ~ Ashley County (71)
- Arkansas ~ Baxter County (72)
- Arkansas ~ Benton County (73)
- Arkansas ~ Boone County (74)
- Arkansas ~ Bradley County (75)
- Arkansas ~ Calhoun County (76)
- Arkansas ~ Carroll County (77)
- Arkansas ~ Chicot County (78)
- Arkansas ~ Clark County (79)
- Arkansas ~ Clay County (80)
- Arkansas ~ Cleburne County (81)
- Arkansas ~ Cleveland County (82)
- Arkansas ~ Columbia County (83)
- Arkansas ~ Conway County (84)
- Arkansas ~ Craighead County (85)
- Arkansas ~ Crawford County (86)
- Arkansas ~ Crittenden County (87)
- Arkansas ~ Cross County (88)
- Arkansas ~ Dallas County (89)
- Arkansas ~ Desha County (90)
- Arkansas ~ Drew County (91)
- Arkansas ~ Faulkner County (92)
- Arkansas ~ Franklin County (93)
- Arkansas ~ Fulton County (94)
- Arkansas ~ Garland County (95)
- Arkansas ~ Grant County (96)
- Arkansas ~ Greene County (97)
- Arkansas ~ Hempstead County (98)
- Arkansas ~ Hot Spring County (99)
- Arkansas ~ Howard County (100)
- Arkansas ~ Independence County (101)
- Arkansas ~ Izard County (102)
- Arkansas ~ Jackson County (103)
- Arkansas ~ Jefferson County (104)
- Arkansas ~ Johnson County (105)
- Arkansas ~ Lafayette County (106)
- Arkansas ~ Lawrence County (107)
- Arkansas ~ Lee County (108)
- Arkansas ~ Lincoln County (109)
- Arkansas ~ Little River County (110)
- Arkansas ~ Logan County (111)
- Arkansas ~ Lonoke County (112)
- Arkansas ~ Madison County (113)
- Arkansas ~ Marion County (114)
- Arkansas ~ Miller County (115)
- Arkansas ~ Mississippi County (116)
- Arkansas ~ Monroe County (117)
- Arkansas ~ Montgomery County (118)
- Arkansas ~ Nevada County (119)
- Arkansas ~ Newton County (120)
- Arkansas ~ Ouachita County (121)
- Arkansas ~ Perry County (122)
- Arkansas ~ Phillips County (123)
- Arkansas ~ Pike County (124)
- Arkansas ~ Poinsett County (125)
- Arkansas ~ Polk County (126)
- Arkansas ~ Pope County (127)
- Arkansas ~ Prairie County (128)
- Arkansas ~ Pulaski County (129)
- Arkansas ~ Randolph County (130)
- Arkansas ~ St. Francis County (131)
- Arkansas ~ Saline County (132)
- Arkansas ~ Scott County (133)
- Arkansas ~ Searcy County (134)
- Arkansas ~ Sebastian County (135)
- Arkansas ~ Sevier County (136)
- Arkansas ~ Sharp County (137)
- Arkansas ~ Stone County (138)
- Arkansas ~ Union County (139)
- Arkansas ~ Van Buren County (140)
- Arkansas ~ Washington County (141)
- Arkansas ~ White County (142)
- Arkansas ~ Woodruff County (143)
- Arkansas ~ Yell County (144)
- Delaware (145)
- Delaware ~ Kent County (146)
- Delaware ~ New Castle County (147)
- Delaware ~ Sussex County (148)
- District of Columbia (149)
- District of Columbia ~ District of Columbia (150)
- Florida (151)
- Florida ~ Alachua County (152)
- Florida ~ Baker County (153)
- Florida ~ Bay County (154)
- Florida ~ Bradford County (155)
- Florida ~ Brevard County (156)
- Florida ~ Broward County (157)
- Florida ~ Calhoun County (158)
- Florida ~ Charlotte County (159)
- Florida ~ Citrus County (160)
- Florida ~ Clay County (161)
- Florida ~ Collier County (162)
- Florida ~ Columbia County (163)
- Florida ~ DeSoto County (164)
- Florida ~ Dixie County (165)
- Florida ~ Duval County (166)
- Florida ~ Escambia County (167)
- Florida ~ Flagler County (168)
- Florida ~ Franklin County (169)
- Florida ~ Gadsden County (170)
- Florida ~ Gilchrist County (171)
- Florida ~ Glades County (172)
- Florida ~ Gulf County (173)
- Florida ~ Hamilton County (174)
- Florida ~ Hardee County (175)
- Florida ~ Hendry County (176)
- Florida ~ Hernando County (177)
- Florida ~ Highlands County (178)
- Florida ~ Hillsborough County (179)
- Florida ~ Holmes County (180)
- Florida ~ Indian River County (181)
- Florida ~ Jackson County (182)
- Florida ~ Jefferson County (183)
- Florida ~ Lafayette County (184)
- Florida ~ Lake County (185)
- Florida ~ Lee County (186)
- Florida ~ Leon County (187)
- Florida ~ Levy County (188)
- Florida ~ Liberty County (189)
- Florida ~ Madison County (190)
- Florida ~ Manatee County (191)
- Florida ~ Marion County (192)
- Florida ~ Martin County (193)
- Florida ~ Miami-Dade County (194)
- Florida ~ Monroe County (195)
- Florida ~ Nassau County (196)
- Florida ~ Okaloosa County (197)
- Florida ~ Okeechobee County (198)
- Florida ~ Orange County (199)
- Florida ~ Osceola County (200)
- Florida ~ Palm Beach County (201)
- Florida ~ Pasco County (202)
- Florida ~ Pinellas County (203)
- Florida ~ Polk County (204)
- Florida ~ Putnam County (205)
- Florida ~ St. Johns County (206)
- Florida ~ St. Lucie County (207)
- Florida ~ Santa Rosa County (208)
- Florida ~ Sarasota County (209)
- Florida ~ Seminole County (210)
- Florida ~ Sumter County (211)
- Florida ~ Suwannee County (212)
- Florida ~ Taylor County (213)
- Florida ~ Union County (214)
- Florida ~ Volusia County (215)
- Florida ~ Wakulla County (216)
- Florida ~ Walton County (217)
- Florida ~ Washington County (218)
- Georgia (219)
- Georgia ~ Appling County (220)
- Georgia ~ Atkinson County (221)
- Georgia ~ Bacon County (222)
- Georgia ~ Baker County (223)
- Georgia ~ Baldwin County (224)
- Georgia ~ Banks County (225)
- Georgia ~ Barrow County (226)
- Georgia ~ Bartow County (227)
- Georgia ~ Ben Hill County (228)
- Georgia ~ Berrien County (229)
- Georgia ~ Bibb County (230)
- Georgia ~ Bleckley County (231)
- Georgia ~ Brantley County (232)
- Georgia ~ Brooks County (233)
- Georgia ~ Bryan County (234)
- Georgia ~ Bulloch County (235)
- Georgia ~ Burke County (236)
- Georgia ~ Butts County (237)
- Georgia ~ Calhoun County (238)
- Georgia ~ Camden County (239)
- Georgia ~ Candler County (240)
- Georgia ~ Carroll County (241)
- Georgia ~ Catoosa County (242)
- Georgia ~ Charlton County (243)
- Georgia ~ Chatham County (244)
- Georgia ~ Chattahoochee County (245)
- Georgia ~ Chattooga County (246)
- Georgia ~ Cherokee County (247)
- Georgia ~ Clarke County (248)
- Georgia ~ Clay County (249)
- Georgia ~ Clayton County (250)
- Georgia ~ Clinch County (251)
- Georgia ~ Cobb County (252)
- Georgia ~ Coffee County (253)
- Georgia ~ Colquitt County (254)
- Georgia ~ Columbia County (255)
- Georgia ~ Cook County (256)
- Georgia ~ Coweta County (257)
- Georgia ~ Crawford County (258)
- Georgia ~ Crisp County (259)
- Georgia ~ Dade County (260)
- Georgia ~ Dawson County (261)
- Georgia ~ Decatur County (262)
- Georgia ~ DeKalb County (263)
- Georgia ~ Dodge County (264)
- Georgia ~ Dooly County (265)
- Georgia ~ Dougherty County (266)
- Georgia ~ Douglas County (267)
- Georgia ~ Early County (268)
- Georgia ~ Echols County (269)
- Georgia ~ Effingham County (270)
- Georgia ~ Elbert County (271)
- Georgia ~ Emanuel County (272)
- Georgia ~ Evans County (273)
- Georgia ~ Fannin County (274)
- Georgia ~ Fayette County (275)
- Georgia ~ Floyd County (276)
- Georgia ~ Forsyth County (277)
- Georgia ~ Franklin County (278)
- Georgia ~ Fulton County (279)
- Georgia ~ Gilmer County (280)
- Georgia ~ Glascock County (281)
- Georgia ~ Glynn County (282)
- Georgia ~ Gordon County (283)
- Georgia ~ Grady County (284)
- Georgia ~ Greene County (285)
- Georgia ~ Gwinnett County (286)
- Georgia ~ Habersham County (287)
- Georgia ~ Hall County (288)
- Georgia ~ Hancock County (289)
- Georgia ~ Haralson County (290)
- Georgia ~ Harris County (291)
- Georgia ~ Hart County (292)
- Georgia ~ Heard County (293)
- Georgia ~ Henry County (294)
- Georgia ~ Houston County (295)
- Georgia ~ Irwin County (296)
- Georgia ~ Jackson County (297)
- Georgia ~ Jasper County (298)
- Georgia ~ Jeff Davis County (299)
- Georgia ~ Jefferson County (300)
- Georgia ~ Jenkins County (301)
- Georgia ~ Johnson County (302)
- Georgia ~ Jones County (303)
- Georgia ~ Lamar County (304)
- Georgia ~ Lanier County (305)
- Georgia ~ Laurens County (306)
- Georgia ~ Lee County (307)
- Georgia ~ Liberty County (308)
- Georgia ~ Lincoln County (309)
- Georgia ~ Long County (310)
- Georgia ~ Lowndes County (311)
- Georgia ~ Lumpkin County (312)
- Georgia ~ McDuffie County (313)
- Georgia ~ McIntosh County (314)
- Georgia ~ Macon County (315)
- Georgia ~ Madison County (316)
- Georgia ~ Marion County (317)
- Georgia ~ Meriwether County (318)
- Georgia ~ Miller County (319)
- Georgia ~ Mitchell County (320)
- Georgia ~ Monroe County (321)
- Georgia ~ Montgomery County (322)
- Georgia ~ Morgan County (323)
- Georgia ~ Murray County (324)
- Georgia ~ Muscogee County (325)
- Georgia ~ Newton County (326)
- Georgia ~ Oconee County (327)
- Georgia ~ Oglethorpe County (328)
- Georgia ~ Paulding County (329)
- Georgia ~ Peach County (330)
- Georgia ~ Pickens County (331)
- Georgia ~ Pierce County (332)
- Georgia ~ Pike County (333)
- Georgia ~ Polk County (334)
- Georgia ~ Pulaski County (335)
- Georgia ~ Putnam County (336)
- Georgia ~ Quitman County (337)
- Georgia ~ Rabun County (338)
- Georgia ~ Randolph County (339)
- Georgia ~ Richmond County (340)
- Georgia ~ Rockdale County (341)
- Georgia ~ Schley County (342)
- Georgia ~ Screven County (343)
- Georgia ~ Seminole County (344)
- Georgia ~ Spalding County (345)
- Georgia ~ Stephens County (346)
- Georgia ~ Stewart County (347)
- Georgia ~ Sumter County (348)
- Georgia ~ Talbot County (349)
- Georgia ~ Taliaferro County (350)
- Georgia ~ Tattnall County (351)
- Georgia ~ Taylor County (352)
- Georgia ~ Telfair County (353)
- Georgia ~ Terrell County (354)
- Georgia ~ Thomas County (355)
- Georgia ~ Tift County (356)
- Georgia ~ Toombs County (357)
- Georgia ~ Towns County (358)
- Georgia ~ Treutlen County (359)
- Georgia ~ Troup County (360)
- Georgia ~ Turner County (361)
- Georgia ~ Twiggs County (362)
- Georgia ~ Union County (363)
- Georgia ~ Upson County (364)
- Georgia ~ Walker County (365)
- Georgia ~ Walton County (366)
- Georgia ~ Ware County (367)
- Georgia ~ Warren County (368)
- Georgia ~ Washington County (369)
- Georgia ~ Wayne County (370)
- Georgia ~ Webster County (371)
- Georgia ~ Wheeler County (372)
- Georgia ~ White County (373)
- Georgia ~ Whitfield County (374)
- Georgia ~ Wilcox County (375)
- Georgia ~ Wilkes County (376)
- Georgia ~ Wilkinson County (377)
- Georgia ~ Worth County (378)
- Kentucky (379)
- Kentucky ~ Adair County (380)
- Kentucky ~ Allen County (381)
- Kentucky ~ Anderson County (382)
- Kentucky ~ Ballard County (383)
- Kentucky ~ Barren County (384)
- Kentucky ~ Bath County (385)
- Kentucky ~ Bell County (386)
- Kentucky ~ Boone County (387)
- Kentucky ~ Bourbon County (388)
- Kentucky ~ Boyd County (389)
- Kentucky ~ Boyle County (390)
- Kentucky ~ Bracken County (391)
- Kentucky ~ Breathitt County (392)
- Kentucky ~ Breckinridge County (393)
- Kentucky ~ Bullitt County (394)
- Kentucky ~ Butler County (395)
- Kentucky ~ Caldwell County (396)
- Kentucky ~ Calloway County (397)
- Kentucky ~ Campbell County (398)
- Kentucky ~ Carlisle County (399)
- Kentucky ~ Carroll County (400)
- Kentucky ~ Carter County (401)
- Kentucky ~ Casey County (402)
- Kentucky ~ Christian County (403)
- Kentucky ~ Clark County (404)
- Kentucky ~ Clay County (405)
- Kentucky ~ Clinton County (406)
- Kentucky ~ Crittenden County (407)
- Kentucky ~ Cumberland County (408)
- Kentucky ~ Daviess County (409)
- Kentucky ~ Edmonson County (410)
- Kentucky ~ Elliott County (411)
- Kentucky ~ Estill County (412)
- Kentucky ~ Fayette County (413)
- Kentucky ~ Fleming County (414)
- Kentucky ~ Floyd County (415)
- Kentucky ~ Franklin County (416)
- Kentucky ~ Fulton County (417)
- Kentucky ~ Gallatin County (418)
- Kentucky ~ Garrard County (419)
- Kentucky ~ Grant County (420)
- Kentucky ~ Graves County (421)
- Kentucky ~ Grayson County (422)
- Kentucky ~ Green County (423)
- Kentucky ~ Greenup County (424)
- Kentucky ~ Hancock County (425)
- Kentucky ~ Hardin County (426)
- Kentucky ~ Harlan County (427)
- Kentucky ~ Harrison County (428)
- Kentucky ~ Hart County (429)
- Kentucky ~ Henderson County (430)
- Kentucky ~ Henry County (431)
- Kentucky ~ Hickman County (432)
- Kentucky ~ Hopkins County (433)
- Kentucky ~ Jackson County (434)
- Kentucky ~ Jefferson County (435)
- Kentucky ~ Jessamine County (436)
- Kentucky ~ Johnson County (437)
- Kentucky ~ Kenton County (438)
- Kentucky ~ Knott County (439)
- Kentucky ~ Knox County (440)
- Kentucky ~ LaRue County (441)
- Kentucky ~ Laurel County (442)
- Kentucky ~ Lawrence County (443)
- Kentucky ~ Lee County (444)
- Kentucky ~ Leslie County (445)
- Kentucky ~ Letcher County (446)
- Kentucky ~ Lewis County (447)
- Kentucky ~ Lincoln County (448)
- Kentucky ~ Livingston County (449)
- Kentucky ~ Logan County (450)
- Kentucky ~ Lyon County (451)
- Kentucky ~ McCracken County (452)
- Kentucky ~ McCreary County (453)
- Kentucky ~ McLean County (454)
- Kentucky ~ Madison County (455)
- Kentucky ~ Magoffin County (456)
- Kentucky ~ Marion County (457)
- Kentucky ~ Marshall County (458)
- Kentucky ~ Martin County (459)
- Kentucky ~ Mason County (460)
- Kentucky ~ Meade County (461)
- Kentucky ~ Menifee County (462)
- Kentucky ~ Mercer County (463)
- Kentucky ~ Metcalfe County (464)
- Kentucky ~ Monroe County (465)
- Kentucky ~ Montgomery County (466)
- Kentucky ~ Morgan County (467)
- Kentucky ~ Muhlenberg County (468)
- Kentucky ~ Nelson County (469)
- Kentucky ~ Nicholas County (470)
- Kentucky ~ Ohio County (471)
- Kentucky ~ Oldham County (472)
- Kentucky ~ Owen County (473)
- Kentucky ~ Owsley County (474)
- Kentucky ~ Pendleton County (475)
- Kentucky ~ Perry County (476)
- Kentucky ~ Pike County (477)
- Kentucky ~ Powell County (478)
- Kentucky ~ Pulaski County (479)
- Kentucky ~ Robertson County (480)
- Kentucky ~ Rockcastle County (481)
- Kentucky ~ Rowan County (482)
- Kentucky ~ Russell County (483)
- Kentucky ~ Scott County (484)
- Kentucky ~ Shelby County (485)
- Kentucky ~ Simpson County (486)
- Kentucky ~ Spencer County (487)
- Kentucky ~ Taylor County (488)
- Kentucky ~ Todd County (489)
- Kentucky ~ Trigg County (490)
- Kentucky ~ Trimble County (491)
- Kentucky ~ Union County (492)
- Kentucky ~ Warren County (493)
- Kentucky ~ Washington County (494)
- Kentucky ~ Wayne County (495)
- Kentucky ~ Webster County (496)
- Kentucky ~ Whitley County (497)
- Kentucky ~ Wolfe County (498)
- Kentucky ~ Woodford County (499)
- Louisiana (500)
- Louisiana ~ Acadia Parish (501)
- Louisiana ~ Allen Parish (502)
- Louisiana ~ Ascension Parish (503)
- Louisiana ~ Assumption Parish (504)
- Louisiana ~ Avoyelles Parish (505)
- Louisiana ~ Beauregard Parish (506)
- Louisiana ~ Bienville Parish (507)
- Louisiana ~ Bossier Parish (508)
- Louisiana ~ Caddo Parish (509)
- Louisiana ~ Calcasieu Parish (510)
- Louisiana ~ Caldwell Parish (511)
- Louisiana ~ Cameron Parish (512)
- Louisiana ~ Catahoula Parish (513)
- Louisiana ~ Claiborne Parish (514)
- Louisiana ~ Concordia Parish (515)
- Louisiana ~ De Soto Parish (516)
- Louisiana ~ East Baton Rouge Parish (517)
- Louisiana ~ East Carroll Parish (518)
- Louisiana ~ East Feliciana Parish (519)
- Louisiana ~ Evangeline Parish (520)
- Louisiana ~ Franklin Parish (521)
- Louisiana ~ Grant Parish (522)
- Louisiana ~ Iberia Parish (523)
- Louisiana ~ Iberville Parish (524)
- Louisiana ~ Jackson Parish (525)
- Louisiana ~ Jefferson Parish (526)
- Louisiana ~ Jefferson Davis Parish (527)
- Louisiana ~ Lafayette Parish (528)
- Louisiana ~ Lafourche Parish (529)
- Louisiana ~ La Salle Parish (530)
- Louisiana ~ Lincoln Parish (531)
- Louisiana ~ Livingston Parish (532)
- Louisiana ~ Madison Parish (533)
- Louisiana ~ Morehouse Parish (534)
- Louisiana ~ Natchitoches Parish (535)
- Louisiana ~ Orleans Parish (536)
- Louisiana ~ Ouachita Parish (537)
- Louisiana ~ Plaquemines Parish (538)
- Louisiana ~ Pointe Coupee Parish (539)
- Louisiana ~ Rapides Parish (540)
- Louisiana ~ Red River Parish (541)
- Louisiana ~ Richland Parish (542)
- Louisiana ~ Sabine Parish (543)
- Louisiana ~ St. Bernard Parish (544)
- Louisiana ~ St. Charles Parish (545)
- Louisiana ~ St. Helena Parish (546)
- Louisiana ~ St. James Parish (547)
- Louisiana ~ St. John the Baptist Parish (548)
- Louisiana ~ St. Landry Parish (549)
- Louisiana ~ St. Martin Parish (550)
- Louisiana ~ St. Mary Parish (551)
- Louisiana ~ St. Tammany Parish (552)
- Louisiana ~ Tangipahoa Parish (553)
- Louisiana ~ Tensas Parish (554)
- Louisiana ~ Terrebonne Parish (555)
- Louisiana ~ Union Parish (556)
- Louisiana ~ Vermilion Parish (557)
- Louisiana ~ Vernon Parish (558)
- Louisiana ~ Washington Parish (559)
- Louisiana ~ Webster Parish (560)
- Louisiana ~ West Baton Rouge Parish (561)
- Louisiana ~ West Carroll Parish (562)
- Louisiana ~ West Feliciana Parish (563)
- Louisiana ~ Winn Parish (564)
- Maryland (565)
- Maryland ~ Allegany County (566)
- Maryland ~ Anne Arundel County (567)
- Maryland ~ Baltimore County (568)
- Maryland ~ Calvert County (569)
- Maryland ~ Caroline County (570)
- Maryland ~ Carroll County (571)
- Maryland ~ Cecil County (572)
- Maryland ~ Charles County (573)
- Maryland ~ Dorchester County (574)
- Maryland ~ Frederick County (575)
- Maryland ~ Garrett County (576)
- Maryland ~ Harford County (577)
- Maryland ~ Howard County (578)
- Maryland ~ Kent County (579)
- Maryland ~ Montgomery County (580)
- Maryland ~ Prince George's County (581)
- Maryland ~ Queen Anne's County (582)
- Maryland ~ St. Mary's County (583)
- Maryland ~ Somerset County (584)
- Maryland ~ Talbot County (585)
- Maryland ~ Washington County (586)
- Maryland ~ Wicomico County (587)
- Maryland ~ Worcester County (588)
- Maryland ~ City of Baltimore (589)
- Mississippi (590)
- Mississippi ~ Adams County (591)
- Mississippi ~ Alcorn County (592)
- Mississippi ~ Amite County (593)
- Mississippi ~ Attala County (594)
- Mississippi ~ Benton County (595)
- Mississippi ~ Bolivar County (596)
- Mississippi ~ Calhoun County (597)
- Mississippi ~ Carroll County (598)
- Mississippi ~ Chickasaw County (599)
- Mississippi ~ Choctaw County (600)
- Mississippi ~ Claiborne County (601)
- Mississippi ~ Clarke County (602)
- Mississippi ~ Clay County (603)
- Mississippi ~ Coahoma County (604)
- Mississippi ~ Copiah County (605)
- Mississippi ~ Covington County (606)
- Mississippi ~ DeSoto County (607)
- Mississippi ~ Forrest County (608)
- Mississippi ~ Franklin County (609)
- Mississippi ~ George County (610)
- Mississippi ~ Greene County (611)
- Mississippi ~ Grenada County (612)
- Mississippi ~ Hancock County (613)
- Mississippi ~ Harrison County (614)
- Mississippi ~ Hinds County (615)
- Mississippi ~ Holmes County (616)
- Mississippi ~ Humphreys County (617)
- Mississippi ~ Issaquena County (618)
- Mississippi ~ Itawamba County (619)
- Mississippi ~ Jackson County (620)
- Mississippi ~ Jasper County (621)
- Mississippi ~ Jefferson County (622)
- Mississippi ~ Jefferson Davis County (623)
- Mississippi ~ Jones County (624)
- Mississippi ~ Kemper County (625)
- Mississippi ~ Lafayette County (626)
- Mississippi ~ Lamar County (627)
- Mississippi ~ Lauderdale County (628)
- Mississippi ~ Lawrence County (629)
- Mississippi ~ Leake County (630)
- Mississippi ~ Lee County (631)
- Mississippi ~ Leflore County (632)
- Mississippi ~ Lincoln County (633)
- Mississippi ~ Lowndes County (634)
- Mississippi ~ Madison County (635)
- Mississippi ~ Marion County (636)
- Mississippi ~ Marshall County (637)
- Mississippi ~ Monroe County (638)
- Mississippi ~ Montgomery County (639)
- Mississippi ~ Neshoba County (640)
- Mississippi ~ Newton County (641)
- Mississippi ~ Noxubee County (642)
- Mississippi ~ Oktibbeha County (643)
- Mississippi ~ Panola County (644)
- Mississippi ~ Pearl River County (645)
- Mississippi ~ Perry County (646)
- Mississippi ~ Pike County (647)
- Mississippi ~ Pontotoc County (648)
- Mississippi ~ Prentiss County (649)
- Mississippi ~ Quitman County (650)
- Mississippi ~ Rankin County (651)
- Mississippi ~ Scott County (652)
- Mississippi ~ Sharkey County (653)
- Mississippi ~ Simpson County (654)
- Mississippi ~ Smith County (655)
- Mississippi ~ Stone County (656)
- Mississippi ~ Sunflower County (657)
- Mississippi ~ Tallahatchie County (658)
- Mississippi ~ Tate County (659)
- Mississippi ~ Tippah County (660)
- Mississippi ~ Tishomingo County (661)
- Mississippi ~ Tunica County (662)
- Mississippi ~ Union County (663)
- Mississippi ~ Walthall County (664)
- Mississippi ~ Warren County (665)
- Mississippi ~ Washington County (666)
- Mississippi ~ Wayne County (667)
- Mississippi ~ Webster County (668)
- Mississippi ~ Wilkinson County (669)
- Mississippi ~ Winston County (670)
- Mississippi ~ Yalobusha County (671)
- Mississippi ~ Yazoo County (672)
- North Carolina (673)
- North Carolina ~ Alamance County (674)
- North Carolina ~ Alexander County (675)
- North Carolina ~ Alleghany County (676)
- North Carolina ~ Anson County (677)
- North Carolina ~ Ashe County (678)
- North Carolina ~ Avery County (679)
- North Carolina ~ Beaufort County (680)
- North Carolina ~ Bertie County (681)
- North Carolina ~ Bladen County (682)
- North Carolina ~ Brunswick County (683)
- North Carolina ~ Buncombe County (684)
- North Carolina ~ Burke County (685)
- North Carolina ~ Cabarrus County (686)
- North Carolina ~ Caldwell County (687)
- North Carolina ~ Camden County (688)
- North Carolina ~ Carteret County (689)
- North Carolina ~ Caswell County (690)
- North Carolina ~ Catawba County (691)
- North Carolina ~ Chatham County (692)
- North Carolina ~ Cherokee County (693)
- North Carolina ~ Chowan County (694)
- North Carolina ~ Clay County (695)
- North Carolina ~ Cleveland County (696)
- North Carolina ~ Columbus County (697)
- North Carolina ~ Craven County (698)
- North Carolina ~ Cumberland County (699)
- North Carolina ~ Currituck County (700)
- North Carolina ~ Dare County (701)
- North Carolina ~ Davidson County (702)
- North Carolina ~ Davie County (703)
- North Carolina ~ Duplin County (704)
- North Carolina ~ Durham County (705)
- North Carolina ~ Edgecombe County (706)
- North Carolina ~ Forsyth County (707)
- North Carolina ~ Franklin County (708)
- North Carolina ~ Gaston County (709)
- North Carolina ~ Gates County (710)
- North Carolina ~ Graham County (711)
- North Carolina ~ Granville County (712)
- North Carolina ~ Greene County (713)
- North Carolina ~ Guilford County (714)
- North Carolina ~ Halifax County (715)
- North Carolina ~ Harnett County (716)
- North Carolina ~ Haywood County (717)
- North Carolina ~ Henderson County (718)
- North Carolina ~ Hertford County (719)
- North Carolina ~ Hoke County (720)
- North Carolina ~ Hyde County (721)
- North Carolina ~ Iredell County (722)
- North Carolina ~ Jackson County (723)
- North Carolina ~ Johnston County (724)
- North Carolina ~ Jones County (725)
- North Carolina ~ Lee County (726)
- North Carolina ~ Lenoir County (727)
- North Carolina ~ Lincoln County (728)
- North Carolina ~ McDowell County (729)
- North Carolina ~ Macon County (730)
- North Carolina ~ Madison County (731)
- North Carolina ~ Martin County (732)
- North Carolina ~ Mecklenburg County (733)
- North Carolina ~ Mitchell County (734)
- North Carolina ~ Montgomery County (735)
- North Carolina ~ Moore County (736)
- North Carolina ~ Nash County (737)
- North Carolina ~ New Hanover County (738)
- North Carolina ~ Northampton County (739)
- North Carolina ~ Onslow County (740)
- North Carolina ~ Orange County (741)
- North Carolina ~ Pamlico County (742)
- North Carolina ~ Pasquotank County (743)
- North Carolina ~ Pender County (744)
- North Carolina ~ Perquimans County (745)
- North Carolina ~ Person County (746)
- North Carolina ~ Pitt County (747)
- North Carolina ~ Polk County (748)
- North Carolina ~ Randolph County (749)
- North Carolina ~ Richmond County (750)
- North Carolina ~ Robeson County (751)
- North Carolina ~ Rockingham County (752)
- North Carolina ~ Rowan County (753)
- North Carolina ~ Rutherford County (754)
- North Carolina ~ Sampson County (755)
- North Carolina ~ Scotland County (756)
- North Carolina ~ Stanly County (757)
- North Carolina ~ Stokes County (758)
- North Carolina ~ Surry County (759)
- North Carolina ~ Swain County (760)
- North Carolina ~ Transylvania County (761)
- North Carolina ~ Tyrrell County (762)
- North Carolina ~ Union County (763)
- North Carolina ~ Vance County (764)
- North Carolina ~ Wake County (765)
- North Carolina ~ Warren County (766)
- North Carolina ~ Washington County (767)
- North Carolina ~ Watauga County (768)
- North Carolina ~ Wayne County (769)
- North Carolina ~ Wilkes County (770)
- North Carolina ~ Wilson County (771)
- North Carolina ~ Yadkin County (772)
- North Carolina ~ Yancey County (773)
- Oklahoma (774)
- Oklahoma ~ Adair County (775)
- Oklahoma ~ Alfalfa County (776)
- Oklahoma ~ Atoka County (777)
- Oklahoma ~ Beaver County (778)
- Oklahoma ~ Beckham County (779)
- Oklahoma ~ Blaine County (780)
- Oklahoma ~ Bryan County (781)
- Oklahoma ~ Caddo County (782)
- Oklahoma ~ Canadian County (783)
- Oklahoma ~ Carter County (784)
- Oklahoma ~ Cherokee County (785)
- Oklahoma ~ Choctaw County (786)
- Oklahoma ~ Cimarron County (787)
- Oklahoma ~ Cleveland County (788)
- Oklahoma ~ Coal County (789)
- Oklahoma ~ Comanche County (790)
- Oklahoma ~ Cotton County (791)
- Oklahoma ~ Craig County (792)
- Oklahoma ~ Creek County (793)
- Oklahoma ~ Custer County (794)
- Oklahoma ~ Delaware County (795)
- Oklahoma ~ Dewey County (796)
- Oklahoma ~ Ellis County (797)
- Oklahoma ~ Garfield County (798)
- Oklahoma ~ Garvin County (799)
- Oklahoma ~ Grady County (800)
- Oklahoma ~ Grant County (801)
- Oklahoma ~ Greer County (802)
- Oklahoma ~ Harmon County (803)
- Oklahoma ~ Harper County (804)
- Oklahoma ~ Haskell County (805)
- Oklahoma ~ Hughes County (806)
- Oklahoma ~ Jackson County (807)
- Oklahoma ~ Jefferson County (808)
- Oklahoma ~ Johnston County (809)
- Oklahoma ~ Kay County (810)
- Oklahoma ~ Kingfisher County (811)
- Oklahoma ~ Kiowa County (812)
- Oklahoma ~ Latimer County (813)
- Oklahoma ~ Le Flore County (814)
- Oklahoma ~ Lincoln County (815)
- Oklahoma ~ Logan County (816)
- Oklahoma ~ Love County (817)
- Oklahoma ~ McClain County (818)
- Oklahoma ~ McCurtain County (819)
- Oklahoma ~ McIntosh County (820)
- Oklahoma ~ Major County (821)
- Oklahoma ~ Marshall County (822)
- Oklahoma ~ Mayes County (823)
- Oklahoma ~ Murray County (824)
- Oklahoma ~ Muskogee County (825)
- Oklahoma ~ Noble County (826)
- Oklahoma ~ Nowata County (827)
- Oklahoma ~ Okfuskee County (828)
- Oklahoma ~ Oklahoma County (829)
- Oklahoma ~ Okmulgee County (830)
- Oklahoma ~ Osage County (831)
- Oklahoma ~ Ottawa County (832)
- Oklahoma ~ Pawnee County (833)
- Oklahoma ~ Payne County (834)
- Oklahoma ~ Pittsburg County (835)
- Oklahoma ~ Pontotoc County (836)
- Oklahoma ~ Pottawatomie County (837)
- Oklahoma ~ Pushmataha County (838)
- Oklahoma ~ Roger Mills County (839)
- Oklahoma ~ Rogers County (840)
- Oklahoma ~ Seminole County (841)
- Oklahoma ~ Sequoyah County (842)
- Oklahoma ~ Stephens County (843)
- Oklahoma ~ Texas County (844)
- Oklahoma ~ Tillman County (845)
- Oklahoma ~ Tulsa County (846)
- Oklahoma ~ Wagoner County (847)
- Oklahoma ~ Washington County (848)
- Oklahoma ~ Washita County (849)
- Oklahoma ~ Woods County (850)
- Oklahoma ~ Woodward County (851)
- South Carolina (852)
- South Carolina ~ Abbeville County (853)
- South Carolina ~ Aiken County (854)
- South Carolina ~ Allendale County (855)
- South Carolina ~ Anderson County (856)
- South Carolina ~ Bamberg County (857)
- South Carolina ~ Barnwell County (858)
- South Carolina ~ Beaufort County (859)
- South Carolina ~ Berkeley County (860)
- South Carolina ~ Calhoun County (861)
- South Carolina ~ Charleston County (862)
- South Carolina ~ Cherokee County (863)
- South Carolina ~ Chester County (864)
- South Carolina ~ Chesterfield County (865)
- South Carolina ~ Clarendon County (866)
- South Carolina ~ Colleton County (867)
- South Carolina ~ Darlington County (868)
- South Carolina ~ Dillon County (869)
- South Carolina ~ Dorchester County (870)
- South Carolina ~ Edgefield County (871)
- South Carolina ~ Fairfield County (872)
- South Carolina ~ Florence County (873)
- South Carolina ~ Georgetown County (874)
- South Carolina ~ Greenville County (875)
- South Carolina ~ Greenwood County (876)
- South Carolina ~ Hampton County (877)
- South Carolina ~ Horry County (878)
- South Carolina ~ Jasper County (879)
- South Carolina ~ Kershaw County (880)
- South Carolina ~ Lancaster County (881)
- South Carolina ~ Laurens County (882)
- South Carolina ~ Lee County (883)
- South Carolina ~ Lexington County (884)
- South Carolina ~ McCormick County (885)
- South Carolina ~ Marion County (886)
- South Carolina ~ Marlboro County (887)
- South Carolina ~ Newberry County (888)
- South Carolina ~ Oconee County (889)
- South Carolina ~ Orangeburg County (890)
- South Carolina ~ Pickens County (891)
- South Carolina ~ Richland County (892)
- South Carolina ~ Saluda County (893)
- South Carolina ~ Spartanburg County (894)
- South Carolina ~ Sumter County (895)
- South Carolina ~ Union County (896)
- South Carolina ~ Williamsburg County (897)
- South Carolina ~ York County (898)
- Tennessee (899)
- Tennessee ~ Anderson County (900)
- Tennessee ~ Bedford County (901)
- Tennessee ~ Benton County (902)
- Tennessee ~ Bledsoe County (903)
- Tennessee ~ Blount County (904)
- Tennessee ~ Bradley County (905)
- Tennessee ~ Campbell County (906)
- Tennessee ~ Cannon County (907)
- Tennessee ~ Carroll County (908)
- Tennessee ~ Carter County (909)
- Tennessee ~ Cheatham County (910)
- Tennessee ~ Chester County (911)
- Tennessee ~ Claiborne County (912)
- Tennessee ~ Clay County (913)
- Tennessee ~ Cocke County (914)
- Tennessee ~ Coffee County (915)
- Tennessee ~ Crockett County (916)
- Tennessee ~ Cumberland County (917)
- Tennessee ~ Davidson County (918)
- Tennessee ~ Decatur County (919)
- Tennessee ~ DeKalb County (920)
- Tennessee ~ Dickson County (921)
- Tennessee ~ Dyer County (922)
- Tennessee ~ Fayette County (923)
- Tennessee ~ Fentress County (924)
- Tennessee ~ Franklin County (925)
- Tennessee ~ Gibson County (926)
- Tennessee ~ Giles County (927)
- Tennessee ~ Grainger County (928)
- Tennessee ~ Greene County (929)
- Tennessee ~ Grundy County (930)
- Tennessee ~ Hamblen County (931)
- Tennessee ~ Hamilton County (932)
- Tennessee ~ Hancock County (933)
- Tennessee ~ Hardeman County (934)
- Tennessee ~ Hardin County (935)
- Tennessee ~ Hawkins County (936)
- Tennessee ~ Haywood County (937)
- Tennessee ~ Henderson County (938)
- Tennessee ~ Henry County (939)
- Tennessee ~ Hickman County (940)
- Tennessee ~ Houston County (941)
- Tennessee ~ Humphreys County (942)
- Tennessee ~ Jackson County (943)
- Tennessee ~ Jefferson County (944)
- Tennessee ~ Johnson County (945)
- Tennessee ~ Knox County (946)
- Tennessee ~ Lake County (947)
- Tennessee ~ Lauderdale County (948)
- Tennessee ~ Lawrence County (949)
- Tennessee ~ Lewis County (950)
- Tennessee ~ Lincoln County (951)
- Tennessee ~ Loudon County (952)
- Tennessee ~ McMinn County (953)
- Tennessee ~ McNairy County (954)
- Tennessee ~ Macon County (955)
- Tennessee ~ Madison County (956)
- Tennessee ~ Marion County (957)
- Tennessee ~ Marshall County (958)
- Tennessee ~ Maury County (959)
- Tennessee ~ Meigs County (960)
- Tennessee ~ Monroe County (961)
- Tennessee ~ Montgomery County (962)
- Tennessee ~ Moore County (963)
- Tennessee ~ Morgan County (964)
- Tennessee ~ Obion County (965)
- Tennessee ~ Overton County (966)
- Tennessee ~ Perry County (967)
- Tennessee ~ Pickett County (968)
- Tennessee ~ Polk County (969)
- Tennessee ~ Putnam County (970)
- Tennessee ~ Rhea County (971)
- Tennessee ~ Roane County (972)
- Tennessee ~ Robertson County (973)
- Tennessee ~ Rutherford County (974)
- Tennessee ~ Scott County (975)
- Tennessee ~ Sequatchie County (976)
- Tennessee ~ Sevier County (977)
- Tennessee ~ Shelby County (978)
- Tennessee ~ Smith County (979)
- Tennessee ~ Stewart County (980)
- Tennessee ~ Sullivan County (981)
- Tennessee ~ Sumner County (982)
- Tennessee ~ Tipton County (983)
- Tennessee ~ Trousdale County (984)
- Tennessee ~ Unicoi County (985)
- Tennessee ~ Union County (986)
- Tennessee ~ Van Buren County (987)
- Tennessee ~ Warren County (988)
- Tennessee ~ Washington County (989)
- Tennessee ~ Wayne County (990)
- Tennessee ~ Weakley County (991)
- Tennessee ~ White County (992)
- Tennessee ~ Williamson County (993)
- Tennessee ~ Wilson County (994)
- Texas (995)
- Texas ~ Anderson County (996)
- Texas ~ Andrews County (997)
- Texas ~ Angelina County (998)
- Texas ~ Aransas County (999)
- Texas ~ Archer County (1000)
- Texas ~ Armstrong County (1001)
- Texas ~ Atascosa County (1002)
- Texas ~ Austin County (1003)
- Texas ~ Bailey County (1004)
- Texas ~ Bandera County (1005)
- Texas ~ Bastrop County (1006)
- Texas ~ Baylor County (1007)
- Texas ~ Bee County (1008)
- Texas ~ Bell County (1009)
- Texas ~ Bexar County (1010)
- Texas ~ Blanco County (1011)
- Texas ~ Borden County (1012)
- Texas ~ Bosque County (1013)
- Texas ~ Bowie County (1014)
- Texas ~ Brazoria County (1015)
- Texas ~ Brazos County (1016)
- Texas ~ Brewster County (1017)
- Texas ~ Briscoe County (1018)
- Texas ~ Brooks County (1019)
- Texas ~ Brown County (1020)
- Texas ~ Burleson County (1021)
- Texas ~ Burnet County (1022)
- Texas ~ Caldwell County (1023)
- Texas ~ Calhoun County (1024)
- Texas ~ Callahan County (1025)
- Texas ~ Cameron County (1026)
- Texas ~ Camp County (1027)
- Texas ~ Carson County (1028)
- Texas ~ Cass County (1029)
- Texas ~ Castro County (1030)
- Texas ~ Chambers County (1031)
- Texas ~ Cherokee County (1032)
- Texas ~ Childress County (1033)
- Texas ~ Clay County (1034)
- Texas ~ Cochran County (1035)
- Texas ~ Coke County (1036)
- Texas ~ Coleman County (1037)
- Texas ~ Collin County (1038)
- Texas ~ Collingsworth County (1039)
- Texas ~ Colorado County (1040)
- Texas ~ Comal County (1041)
- Texas ~ Comanche County (1042)
- Texas ~ Concho County (1043)
- Texas ~ Cooke County (1044)
- Texas ~ Coryell County (1045)
- Texas ~ Cottle County (1046)
- Texas ~ Crane County (1047)
- Texas ~ Crockett County (1048)
- Texas ~ Crosby County (1049)
- Texas ~ Culberson County (1050)
- Texas ~ Dallam County (1051)
- Texas ~ Dallas County (1052)
- Texas ~ Dawson County (1053)
- Texas ~ Deaf Smith County (1054)
- Texas ~ Delta County (1055)
- Texas ~ Denton County (1056)
- Texas ~ DeWitt County (1057)
- Texas ~ Dickens County (1058)
- Texas ~ Dimmit County (1059)
- Texas ~ Donley County (1060)
- Texas ~ Duval County (1061)
- Texas ~ Eastland County (1062)
- Texas ~ Ector County (1063)
- Texas ~ Edwards County (1064)
- Texas ~ Ellis County (1065)
- Texas ~ El Paso County (1066)
- Texas ~ Erath County (1067)
- Texas ~ Falls County (1068)
- Texas ~ Fannin County (1069)
- Texas ~ Fayette County (1070)
- Texas ~ Fisher County (1071)
- Texas ~ Floyd County (1072)
- Texas ~ Foard County (1073)
- Texas ~ Fort Bend County (1074)
- Texas ~ Franklin County (1075)
- Texas ~ Freestone County (1076)
- Texas ~ Frio County (1077)
- Texas ~ Gaines County (1078)
- Texas ~ Galveston County (1079)
- Texas ~ Garza County (1080)
- Texas ~ Gillespie County (1081)
- Texas ~ Glasscock County (1082)
- Texas ~ Goliad County (1083)
- Texas ~ Gonzales County (1084)
- Texas ~ Gray County (1085)
- Texas ~ Grayson County (1086)
- Texas ~ Gregg County (1087)
- Texas ~ Grimes County (1088)
- Texas ~ Guadalupe County (1089)
- Texas ~ Hale County (1090)
- Texas ~ Hall County (1091)
- Texas ~ Hamilton County (1092)
- Texas ~ Hansford County (1093)
- Texas ~ Hardeman County (1094)
- Texas ~ Hardin County (1095)
- Texas ~ Harris County (1096)
- Texas ~ Harrison County (1097)
- Texas ~ Hartley County (1098)
- Texas ~ Haskell County (1099)
- Texas ~ Hays County (1100)
- Texas ~ Hemphill County (1101)
- Texas ~ Henderson County (1102)
- Texas ~ Hidalgo County (1103)
- Texas ~ Hill County (1104)
- Texas ~ Hockley County (1105)
- Texas ~ Hood County (1106)
- Texas ~ Hopkins County (1107)
- Texas ~ Houston County (1108)
- Texas ~ Howard County (1109)
- Texas ~ Hudspeth County (1110)
- Texas ~ Hunt County (1111)
- Texas ~ Hutchinson County (1112)
- Texas ~ Irion County (1113)
- Texas ~ Jack County (1114)
- Texas ~ Jackson County (1115)
- Texas ~ Jasper County (1116)
- Texas ~ Jeff Davis County (1117)
- Texas ~ Jefferson County (1118)
- Texas ~ Jim Hogg County (1119)
- Texas ~ Jim Wells County (1120)
- Texas ~ Johnson County (1121)
- Texas ~ Jones County (1122)
- Texas ~ Karnes County (1123)
- Texas ~ Kaufman County (1124)
- Texas ~ Kendall County (1125)
- Texas ~ Kenedy County (1126)
- Texas ~ Kent County (1127)
- Texas ~ Kerr County (1128)
- Texas ~ Kimble County (1129)
- Texas ~ King County (1130)
- Texas ~ Kinney County (1131)
- Texas ~ Kleberg County (1132)
- Texas ~ Knox County (1133)
- Texas ~ Lamar County (1134)
- Texas ~ Lamb County (1135)
- Texas ~ Lampasas County (1136)
- Texas ~ La Salle County (1137)
- Texas ~ Lavaca County (1138)
- Texas ~ Lee County (1139)
- Texas ~ Leon County (1140)
- Texas ~ Liberty County (1141)
- Texas ~ Limestone County (1142)
- Texas ~ Lipscomb County (1143)
- Texas ~ Live Oak County (1144)
- Texas ~ Llano County (1145)
- Texas ~ Loving County (1146)
- Texas ~ Lubbock County (1147)
- Texas ~ Lynn County (1148)
- Texas ~ McCulloch County (1149)
- Texas ~ McLennan County (1150)
- Texas ~ McMullen County (1151)
- Texas ~ Madison County (1152)
- Texas ~ Marion County (1153)
- Texas ~ Martin County (1154)
- Texas ~ Mason County (1155)
- Texas ~ Matagorda County (1156)
- Texas ~ Maverick County (1157)
- Texas ~ Medina County (1158)
- Texas ~ Menard County (1159)
- Texas ~ Midland County (1160)
- Texas ~ Milam County (1161)
- Texas ~ Mills County (1162)
- Texas ~ Mitchell County (1163)
- Texas ~ Montague County (1164)
- Texas ~ Montgomery County (1165)
- Texas ~ Moore County (1166)
- Texas ~ Morris County (1167)
- Texas ~ Motley County (1168)
- Texas ~ Nacogdoches County (1169)
- Texas ~ Navarro County (1170)
- Texas ~ Newton County (1171)
- Texas ~ Nolan County (1172)
- Texas ~ Nueces County (1173)
- Texas ~ Ochiltree County (1174)
- Texas ~ Oldham County (1175)
- Texas ~ Orange County (1176)
- Texas ~ Palo Pinto County (1177)
- Texas ~ Panola County (1178)
- Texas ~ Parker County (1179)
- Texas ~ Parmer County (1180)
- Texas ~ Pecos County (1181)
- Texas ~ Polk County (1182)
- Texas ~ Potter County (1183)
- Texas ~ Presidio County (1184)
- Texas ~ Rains County (1185)
- Texas ~ Randall County (1186)
- Texas ~ Reagan County (1187)
- Texas ~ Real County (1188)
- Texas ~ Red River County (1189)
- Texas ~ Reeves County (1190)
- Texas ~ Refugio County (1191)
- Texas ~ Roberts County (1192)
- Texas ~ Robertson County (1193)
- Texas ~ Rockwall County (1194)
- Texas ~ Runnels County (1195)
- Texas ~ Rusk County (1196)
- Texas ~ Sabine County (1197)
- Texas ~ San Augustine County (1198)
- Texas ~ San Jacinto County (1199)
- Texas ~ San Patricio County (1200)
- Texas ~ San Saba County (1201)
- Texas ~ Schleicher County (1202)
- Texas ~ Scurry County (1203)
- Texas ~ Shackelford County (1204)
- Texas ~ Shelby County (1205)
- Texas ~ Sherman County (1206)
- Texas ~ Smith County (1207)
- Texas ~ Somervell County (1208)
- Texas ~ Starr County (1209)
- Texas ~ Stephens County (1210)
- Texas ~ Sterling County (1211)
- Texas ~ Stonewall County (1212)
- Texas ~ Sutton County (1213)
- Texas ~ Swisher County (1214)
- Texas ~ Tarrant County (1215)
- Texas ~ Taylor County (1216)
- Texas ~ Terrell County (1217)
- Texas ~ Terry County (1218)
- Texas ~ Throckmorton County (1219)
- Texas ~ Titus County (1220)
- Texas ~ Tom Green County (1221)
- Texas ~ Travis County (1222)
- Texas ~ Trinity County (1223)
- Texas ~ Tyler County (1224)
- Texas ~ Upshur County (1225)
- Texas ~ Upton County (1226)
- Texas ~ Uvalde County (1227)
- Texas ~ Val Verde County (1228)
- Texas ~ Van Zandt County (1229)
- Texas ~ Victoria County (1230)
- Texas ~ Walker County (1231)
- Texas ~ Waller County (1232)
- Texas ~ Ward County (1233)
- Texas ~ Washington County (1234)
- Texas ~ Webb County (1235)
- Texas ~ Wharton County (1236)
- Texas ~ Wheeler County (1237)
- Texas ~ Wichita County (1238)
- Texas ~ Wilbarger County (1239)
- Texas ~ Willacy County (1240)
- Texas ~ Williamson County (1241)
- Texas ~ Wilson County (1242)
- Texas ~ Winkler County (1243)
- Texas ~ Wise County (1244)
- Texas ~ Wood County (1245)
- Texas ~ Yoakum County (1246)
- Texas ~ Young County (1247)
- Texas ~ Zapata County (1248)
- Texas ~ Zavala County (1249)
- Virginia (1250)
- Virginia ~ Accomack County (1251)
- Virginia ~ Albemarle County (1252)
- Virginia ~ Alleghany County (1253)
- Virginia ~ Amelia County (1254)
- Virginia ~ Amherst County (1255)
- Virginia ~ Appomattox County (1256)
- Virginia ~ Arlington County (1257)
- Virginia ~ Augusta County (1258)
- Virginia ~ Bath County (1259)
- Virginia ~ Bedford County (1260)
- Virginia ~ Bland County (1261)
- Virginia ~ Botetourt County (1262)
- Virginia ~ Brunswick County (1263)
- Virginia ~ Buchanan County (1264)
- Virginia ~ Buckingham County (1265)
- Virginia ~ Campbell County (1266)
- Virginia ~ Caroline County (1267)
- Virginia ~ Carroll County (1268)
- Virginia ~ Charles City County (1269)
- Virginia ~ Charlotte County (1270)
- Virginia ~ Chesterfield County (1271)
- Virginia ~ Clarke County (1272)
- Virginia ~ Craig County (1273)
- Virginia ~ Culpeper County (1274)
- Virginia ~ Cumberland County (1275)
- Virginia ~ Dickenson County (1276)
- Virginia ~ Dinwiddie County (1277)
- Virginia ~ Essex County (1278)
- Virginia ~ Fairfax County (1279)
- Virginia ~ Fauquier County (1280)
- Virginia ~ Floyd County (1281)
- Virginia ~ Fluvanna County (1282)
- Virginia ~ Franklin County (1283)
- Virginia ~ Frederick County (1284)
- Virginia ~ Giles County (1285)
- Virginia ~ Gloucester County (1286)
- Virginia ~ Goochland County (1287)
- Virginia ~ Grayson County (1288)
- Virginia ~ Greene County (1289)
- Virginia ~ Greensville County (1290)
- Virginia ~ Halifax County (1291)
- Virginia ~ Hanover County (1292)
- Virginia ~ Henrico County (1293)
- Virginia ~ Henry County (1294)
- Virginia ~ Highland County (1295)
- Virginia ~ Isle of Wight County (1296)
- Virginia ~ James City County (1297)
- Virginia ~ King and Queen County (1298)
- Virginia ~ King George County (1299)
- Virginia ~ King William County (1300)
- Virginia ~ Lancaster County (1301)
- Virginia ~ Lee County (1302)
- Virginia ~ Loudoun County (1303)
- Virginia ~ Louisa County (1304)
- Virginia ~ Lunenburg County (1305)
- Virginia ~ Madison County (1306)
- Virginia ~ Mathews County (1307)
- Virginia ~ Mecklenburg County (1308)
- Virginia ~ Middlesex County (1309)
- Virginia ~ Montgomery County (1310)
- Virginia ~ Nelson County (1311)
- Virginia ~ New Kent County (1312)
- Virginia ~ Northampton County (1313)
- Virginia ~ Northumberland County (1314)
- Virginia ~ Nottoway County (1315)
- Virginia ~ Orange County (1316)
- Virginia ~ Page County (1317)
- Virginia ~ Patrick County (1318)
- Virginia ~ Pittsylvania County (1319)
- Virginia ~ Powhatan County (1320)
- Virginia ~ Prince Edward County (1321)
- Virginia ~ Prince George County (1322)
- Virginia ~ Prince William County (1323)
- Virginia ~ Pulaski County (1324)
- Virginia ~ Rappahannock County (1325)
- Virginia ~ Richmond County (1326)
- Virginia ~ Roanoke County (1327)
- Virginia ~ Rockbridge County (1328)
- Virginia ~ Rockingham County (1329)
- Virginia ~ Russell County (1330)
- Virginia ~ Scott County (1331)
- Virginia ~ Shenandoah County (1332)
- Virginia ~ Smyth County (1333)
- Virginia ~ Southampton County (1334)
- Virginia ~ Spotsylvania County (1335)
- Virginia ~ Stafford County (1336)
- Virginia ~ Surry County (1337)
- Virginia ~ Sussex County (1338)
- Virginia ~ Tazewell County (1339)
- Virginia ~ Warren County (1340)
- Virginia ~ Washington County (1341)
- Virginia ~ Westmoreland County (1342)
- Virginia ~ Wise County (1343)
- Virginia ~ Wythe County (1344)
- Virginia ~ York County (1345)
- Virginia ~ City of Alexandria (1346)
- Virginia ~ City of Bristol (1347)
- Virginia ~ City of Buena Vista (1348)
- Virginia ~ City of Charlottesville (1349)
- Virginia ~ City of Chesapeake (1350)
- Virginia ~ City of Colonial Heights (1351)
- Virginia ~ City of Covington (1352)
- Virginia ~ City of Danville (1353)
- Virginia ~ City of Emporia (1354)
- Virginia ~ City of Fairfax (1355)
- Virginia ~ City of Falls Church (1356)
- Virginia ~ City of Franklin (1357)
- Virginia ~ City of Fredericksburg (1358)
- Virginia ~ City of Galax (1359)
- Virginia ~ City of Hampton (1360)
- Virginia ~ City of Harrisonburg (1361)
- Virginia ~ City of Hopewell (1362)
- Virginia ~ City of Lexington (1363)
- Virginia ~ City of Lynchburg (1364)
- Virginia ~ City of Manassas (1365)
- Virginia ~ City of Manassas Park (1366)
- Virginia ~ City of Martinsville (1367)
- Virginia ~ City of Newport News (1368)
- Virginia ~ City of Norfolk (1369)
- Virginia ~ City of Norton (1370)
- Virginia ~ City of Petersburg (1371)
- Virginia ~ City of Poquoson (1372)
- Virginia ~ City of Portsmouth (1373)
- Virginia ~ City of Radford (1374)
- Virginia ~ City of Richmond (1375)
- Virginia ~ City of Roanoke (1376)
- Virginia ~ City of Salem (1377)
- Virginia ~ City of Staunton (1378)
- Virginia ~ City of Suffolk (1379)
- Virginia ~ City of Virginia Beach (1380)
- Virginia ~ City of Waynesboro (1381)
- Virginia ~ City of Williamsburg (1382)
- Virginia ~ City of Winchester (1383)
- West Virginia (1384)
- West Virginia ~ Barbour County (1385)
- West Virginia ~ Berkeley County (1386)
- West Virginia ~ Boone County (1387)
- West Virginia ~ Braxton County (1388)
- West Virginia ~ Brooke County (1389)
- West Virginia ~ Cabell County (1390)
- West Virginia ~ Calhoun County (1391)
- West Virginia ~ Clay County (1392)
- West Virginia ~ Doddridge County (1393)
- West Virginia ~ Fayette County (1394)
- West Virginia ~ Gilmer County (1395)
- West Virginia ~ Grant County (1396)
- West Virginia ~ Greenbrier County (1397)
- West Virginia ~ Hampshire County (1398)
- West Virginia ~ Hancock County (1399)
- West Virginia ~ Hardy County (1400)
- West Virginia ~ Harrison County (1401)
- West Virginia ~ Jackson County (1402)
- West Virginia ~ Jefferson County (1403)
- West Virginia ~ Kanawha County (1404)
- West Virginia ~ Lewis County (1405)
- West Virginia ~ Lincoln County (1406)
- West Virginia ~ Logan County (1407)
- West Virginia ~ McDowell County (1408)
- West Virginia ~ Marion County (1409)
- West Virginia ~ Marshall County (1410)
- West Virginia ~ Mason County (1411)
- West Virginia ~ Mercer County (1412)
- West Virginia ~ Mineral County (1413)
- West Virginia ~ Mingo County (1414)
- West Virginia ~ Monongalia County (1415)
- West Virginia ~ Monroe County (1416)
- West Virginia ~ Morgan County (1417)
- West Virginia ~ Nicholas County (1418)
- West Virginia ~ Ohio County (1419)
- West Virginia ~ Pendleton County (1420)
- West Virginia ~ Pleasants County (1421)
- West Virginia ~ Pocahontas County (1422)
- West Virginia ~ Preston County (1423)
- West Virginia ~ Putnam County (1424)
- West Virginia ~ Raleigh County (1425)
- West Virginia ~ Randolph County (1426)
- West Virginia ~ Ritchie County (1427)
- West Virginia ~ Roane County (1428)
- West Virginia ~ Summers County (1429)
- West Virginia ~ Taylor County (1430)
- West Virginia ~ Tucker County (1431)
- West Virginia ~ Tyler County (1432)
- West Virginia ~ Upshur County (1433)
- West Virginia ~ Wayne County (1434)
- West Virginia ~ Webster County (1435)
- West Virginia ~ Wetzel County (1436)
- West Virginia ~ Wirt County (1437)
- West Virginia ~ Wood County (1438)
- West Virginia ~ Wyoming County (1439)

Display This Question:

If My region of residence is: ___________. Select one option from the drop down menu. = Midwest (IL, IN, IA, KS, MI, MN, MO, NE, ND, OH, SD, WI)

Q1.12 My state and county of residence in the Midwest is:

State (1)

County (2)

- Illinois (1)
- Illinois ~ Adams County (2)
- Illinois ~ Alexander County (3)
- Illinois ~ Bond County (4)
- Illinois ~ Boone County (5)
- Illinois ~ Brown County (6)
- Illinois ~ Bureau County (7)
- Illinois ~ Calhoun County (8)
- Illinois ~ Carroll County (9)
- Illinois ~ Cass County (10)
- Illinois ~ Champaign County (11)
- Illinois ~ Christian County (12)
- Illinois ~ Clark County (13)
- Illinois ~ Clay County (14)
- Illinois ~ Clinton County (15)
- Illinois ~ Coles County (16)
- Illinois ~ Cook County (17)
- Illinois ~ Crawford County (18)
- Illinois ~ Cumberland County (19)
- Illinois ~ DeKalb County (20)
- Illinois ~ De Witt County (21)
- Illinois ~ Douglas County (22)
- Illinois ~ DuPage County (23)
- Illinois ~ Edgar County (24)
- Illinois ~ Edwards County (25)
- Illinois ~ Effingham County (26)
- Illinois ~ Fayette County (27)
- Illinois ~ Ford County (28)
- Illinois ~ Franklin County (29)
- Illinois ~ Fulton County (30)
- Illinois ~ Gallatin County (31)
- Illinois ~ Greene County (32)
- Illinois ~ Grundy County (33)
- Illinois ~ Hamilton County (34)
- Illinois ~ Hancock County (35)
- Illinois ~ Hardin County (36)
- Illinois ~ Henderson County (37)
- Illinois ~ Henry County (38)
- Illinois ~ Iroquois County (39)
- Illinois ~ Jackson County (40)
- Illinois ~ Jasper County (41)
- Illinois ~ Jefferson County (42)
- Illinois ~ Jersey County (43)
- Illinois ~ Jo Daviess County (44)
- Illinois ~ Johnson County (45)
- Illinois ~ Kane County (46)
- Illinois ~ Kankakee County (47)
- Illinois ~ Kendall County (48)
- Illinois ~ Knox County (49)
- Illinois ~ Lake County (50)
- Illinois ~ LaSalle County (51)
- Illinois ~ Lawrence County (52)
- Illinois ~ Lee County (53)
- Illinois ~ Livingston County (54)
- Illinois ~ Logan County (55)
- Illinois ~ McDonough County (56)
- Illinois ~ McHenry County (57)
- Illinois ~ McLean County (58)
- Illinois ~ Macon County (59)
- Illinois ~ Macoupin County (60)
- Illinois ~ Madison County (61)
- Illinois ~ Marion County (62)
- Illinois ~ Marshall County (63)
- Illinois ~ Mason County (64)
- Illinois ~ Massac County (65)
- Illinois ~ Menard County (66)
- Illinois ~ Mercer County (67)
- Illinois ~ Monroe County (68)
- Illinois ~ Montgomery County (69)
- Illinois ~ Morgan County (70)
- Illinois ~ Moultrie County (71)
- Illinois ~ Ogle County (72)
- Illinois ~ Peoria County (73)
- Illinois ~ Perry County (74)
- Illinois ~ Piatt County (75)
- Illinois ~ Pike County (76)
- Illinois ~ Pope County (77)
- Illinois ~ Pulaski County (78)
- Illinois ~ Putnam County (79)
- Illinois ~ Randolph County (80)
- Illinois ~ Richland County (81)
- Illinois ~ Rock Island County (82)
- Illinois ~ St. Clair County (83)
- Illinois ~ Saline County (84)
- Illinois ~ Sangamon County (85)
- Illinois ~ Schuyler County (86)
- Illinois ~ Scott County (87)
- Illinois ~ Shelby County (88)
- Illinois ~ Stark County (89)
- Illinois ~ Stephenson County (90)
- Illinois ~ Tazewell County (91)
- Illinois ~ Union County (92)
- Illinois ~ Vermilion County (93)
- Illinois ~ Wabash County (94)
- Illinois ~ Warren County (95)
- Illinois ~ Washington County (96)
- Illinois ~ Wayne County (97)
- Illinois ~ White County (98)
- Illinois ~ Whiteside County (99)
- Illinois ~ Will County (100)
- Illinois ~ Williamson County (101)
- Illinois ~ Winnebago County (102)
- Illinois ~ Woodford County (103)
- Indiana (104)
- Indiana ~ Adams County (105)
- Indiana ~ Allen County (106)
- Indiana ~ Bartholomew County (107)
- Indiana ~ Benton County (108)
- Indiana ~ Blackford County (109)
- Indiana ~ Boone County (110)
- Indiana ~ Brown County (111)
- Indiana ~ Carroll County (112)
- Indiana ~ Cass County (113)
- Indiana ~ Clark County (114)
- Indiana ~ Clay County (115)
- Indiana ~ Clinton County (116)
- Indiana ~ Crawford County (117)
- Indiana ~ Daviess County (118)
- Indiana ~ Dearborn County (119)
- Indiana ~ Decatur County (120)
- Indiana ~ DeKalb County (121)
- Indiana ~ Delaware County (122)
- Indiana ~ Dubois County (123)
- Indiana ~ Elkhart County (124)
- Indiana ~ Fayette County (125)
- Indiana ~ Floyd County (126)
- Indiana ~ Fountain County (127)
- Indiana ~ Franklin County (128)
- Indiana ~ Fulton County (129)
- Indiana ~ Gibson County (130)
- Indiana ~ Grant County (131)
- Indiana ~ Greene County (132)
- Indiana ~ Hamilton County (133)
- Indiana ~ Hancock County (134)
- Indiana ~ Harrison County (135)
- Indiana ~ Hendricks County (136)
- Indiana ~ Henry County (137)
- Indiana ~ Howard County (138)
- Indiana ~ Huntington County (139)
- Indiana ~ Jackson County (140)
- Indiana ~ Jasper County (141)
- Indiana ~ Jay County (142)
- Indiana ~ Jefferson County (143)
- Indiana ~ Jennings County (144)
- Indiana ~ Johnson County (145)
- Indiana ~ Knox County (146)
- Indiana ~ Kosciusko County (147)
- Indiana ~ LaGrange County (148)
- Indiana ~ Lake County (149)
- Indiana ~ LaPorte County (150)
- Indiana ~ Lawrence County (151)
- Indiana ~ Madison County (152)
- Indiana ~ Marion County (153)
- Indiana ~ Marshall County (154)
- Indiana ~ Martin County (155)
- Indiana ~ Miami County (156)
- Indiana ~ Monroe County (157)
- Indiana ~ Montgomery County (158)
- Indiana ~ Morgan County (159)
- Indiana ~ Newton County (160)
- Indiana ~ Noble County (161)
- Indiana ~ Ohio County (162)
- Indiana ~ Orange County (163)
- Indiana ~ Owen County (164)
- Indiana ~ Parke County (165)
- Indiana ~ Perry County (166)
- Indiana ~ Pike County (167)
- Indiana ~ Porter County (168)
- Indiana ~ Posey County (169)
- Indiana ~ Pulaski County (170)
- Indiana ~ Putnam County (171)
- Indiana ~ Randolph County (172)
- Indiana ~ Ripley County (173)
- Indiana ~ Rush County (174)
- Indiana ~ St. Joseph County (175)
- Indiana ~ Scott County (176)
- Indiana ~ Shelby County (177)
- Indiana ~ Spencer County (178)
- Indiana ~ Starke County (179)
- Indiana ~ Steuben County (180)
- Indiana ~ Sullivan County (181)
- Indiana ~ Switzerland County (182)
- Indiana ~ Tippecanoe County (183)
- Indiana ~ Tipton County (184)
- Indiana ~ Union County (185)
- Indiana ~ Vanderburgh County (186)
- Indiana ~ Vermillion County (187)
- Indiana ~ Vigo County (188)
- Indiana ~ Wabash County (189)
- Indiana ~ Warren County (190)
- Indiana ~ Warrick County (191)
- Indiana ~ Washington County (192)
- Indiana ~ Wayne County (193)
- Indiana ~ Wells County (194)
- Indiana ~ White County (195)
- Indiana ~ Whitley County (196)
- Iowa (197)
- Iowa ~ Adair County (198)
- Iowa ~ Adams County (199)
- Iowa ~ Allamakee County (200)
- Iowa ~ Appanoose County (201)
- Iowa ~ Audubon County (202)
- Iowa ~ Benton County (203)
- Iowa ~ Black Hawk County (204)
- Iowa ~ Boone County (205)
- Iowa ~ Bremer County (206)
- Iowa ~ Buchanan County (207)
- Iowa ~ Buena Vista County (208)
- Iowa ~ Butler County (209)
- Iowa ~ Calhoun County (210)
- Iowa ~ Carroll County (211)
- Iowa ~ Cass County (212)
- Iowa ~ Cedar County (213)
- Iowa ~ Cerro Gordo County (214)
- Iowa ~ Cherokee County (215)
- Iowa ~ Chickasaw County (216)
- Iowa ~ Clarke County (217)
- Iowa ~ Clay County (218)
- Iowa ~ Clayton County (219)
- Iowa ~ Clinton County (220)
- Iowa ~ Crawford County (221)
- Iowa ~ Dallas County (222)
- Iowa ~ Davis County (223)
- Iowa ~ Decatur County (224)
- Iowa ~ Delaware County (225)
- Iowa ~ Des Moines County (226)
- Iowa ~ Dickinson County (227)
- Iowa ~ Dubuque County (228)
- Iowa ~ Emmet County (229)
- Iowa ~ Fayette County (230)
- Iowa ~ Floyd County (231)
- Iowa ~ Franklin County (232)
- Iowa ~ Fremont County (233)
- Iowa ~ Greene County (234)
- Iowa ~ Grundy County (235)
- Iowa ~ Guthrie County (236)
- Iowa ~ Hamilton County (237)
- Iowa ~ Hancock County (238)
- Iowa ~ Hardin County (239)
- Iowa ~ Harrison County (240)
- Iowa ~ Henry County (241)
- Iowa ~ Howard County (242)
- Iowa ~ Humboldt County (243)
- Iowa ~ Ida County (244)
- Iowa ~ Iowa County (245)
- Iowa ~ Jackson County (246)
- Iowa ~ Jasper County (247)
- Iowa ~ Jefferson County (248)
- Iowa ~ Johnson County (249)
- Iowa ~ Jones County (250)
- Iowa ~ Keokuk County (251)
- Iowa ~ Kossuth County (252)
- Iowa ~ Lee County (253)
- Iowa ~ Linn County (254)
- Iowa ~ Louisa County (255)
- Iowa ~ Lucas County (256)
- Iowa ~ Lyon County (257)
- Iowa ~ Madison County (258)
- Iowa ~ Mahaska County (259)
- Iowa ~ Marion County (260)
- Iowa ~ Marshall County (261)
- Iowa ~ Mills County (262)
- Iowa ~ Mitchell County (263)
- Iowa ~ Monona County (264)
- Iowa ~ Monroe County (265)
- Iowa ~ Montgomery County (266)
- Iowa ~ Muscatine County (267)
- Iowa ~ O'Brien County (268)
- Iowa ~ Osceola County (269)
- Iowa ~ Page County (270)
- Iowa ~ Palo Alto County (271)
- Iowa ~ Plymouth County (272)
- Iowa ~ Pocahontas County (273)
- Iowa ~ Polk County (274)
- Iowa ~ Pottawattamie County (275)
- Iowa ~ Poweshiek County (276)
- Iowa ~ Ringgold County (277)
- Iowa ~ Sac County (278)
- Iowa ~ Scott County (279)
- Iowa ~ Shelby County (280)
- Iowa ~ Sioux County (281)
- Iowa ~ Story County (282)
- Iowa ~ Tama County (283)
- Iowa ~ Taylor County (284)
- Iowa ~ Union County (285)
- Iowa ~ Van Buren County (286)
- Iowa ~ Wapello County (287)
- Iowa ~ Warren County (288)
- Iowa ~ Washington County (289)
- Iowa ~ Wayne County (290)
- Iowa ~ Webster County (291)
- Iowa ~ Winnebago County (292)
- Iowa ~ Winneshiek County (293)
- Iowa ~ Woodbury County (294)
- Iowa ~ Worth County (295)
- Iowa ~ Wright County (296)
- Kansas (297)
- Kansas ~ Allen County (298)
- Kansas ~ Anderson County (299)
- Kansas ~ Atchison County (300)
- Kansas ~ Barber County (301)
- Kansas ~ Barton County (302)
- Kansas ~ Bourbon County (303)
- Kansas ~ Brown County (304)
- Kansas ~ Butler County (305)
- Kansas ~ Chase County (306)
- Kansas ~ Chautauqua County (307)
- Kansas ~ Cherokee County (308)
- Kansas ~ Cheyenne County (309)
- Kansas ~ Clark County (310)
- Kansas ~ Clay County (311)
- Kansas ~ Cloud County (312)
- Kansas ~ Coffey County (313)
- Kansas ~ Comanche County (314)
- Kansas ~ Cowley County (315)
- Kansas ~ Crawford County (316)
- Kansas ~ Decatur County (317)
- Kansas ~ Dickinson County (318)
- Kansas ~ Doniphan County (319)
- Kansas ~ Douglas County (320)
- Kansas ~ Edwards County (321)
- Kansas ~ Elk County (322)
- Kansas ~ Ellis County (323)
- Kansas ~ Ellsworth County (324)
- Kansas ~ Finney County (325)
- Kansas ~ Ford County (326)
- Kansas ~ Franklin County (327)
- Kansas ~ Geary County (328)
- Kansas ~ Gove County (329)
- Kansas ~ Graham County (330)
- Kansas ~ Grant County (331)
- Kansas ~ Gray County (332)
- Kansas ~ Greeley County (333)
- Kansas ~ Greenwood County (334)
- Kansas ~ Hamilton County (335)
- Kansas ~ Harper County (336)
- Kansas ~ Harvey County (337)
- Kansas ~ Haskell County (338)
- Kansas ~ Hodgeman County (339)
- Kansas ~ Jackson County (340)
- Kansas ~ Jefferson County (341)
- Kansas ~ Jewell County (342)
- Kansas ~ Johnson County (343)
- Kansas ~ Kearny County (344)
- Kansas ~ Kingman County (345)
- Kansas ~ Kiowa County (346)
- Kansas ~ Labette County (347)
- Kansas ~ Lane County (348)
- Kansas ~ Leavenworth County (349)
- Kansas ~ Lincoln County (350)
- Kansas ~ Linn County (351)
- Kansas ~ Logan County (352)
- Kansas ~ Lyon County (353)
- Kansas ~ McPherson County (354)
- Kansas ~ Marion County (355)
- Kansas ~ Marshall County (356)
- Kansas ~ Meade County (357)
- Kansas ~ Miami County (358)
- Kansas ~ Mitchell County (359)
- Kansas ~ Montgomery County (360)
- Kansas ~ Morris County (361)
- Kansas ~ Morton County (362)
- Kansas ~ Nemaha County (363)
- Kansas ~ Neosho County (364)
- Kansas ~ Ness County (365)
- Kansas ~ Norton County (366)
- Kansas ~ Osage County (367)
- Kansas ~ Osborne County (368)
- Kansas ~ Ottawa County (369)
- Kansas ~ Pawnee County (370)
- Kansas ~ Phillips County (371)
- Kansas ~ Pottawatomie County (372)
- Kansas ~ Pratt County (373)
- Kansas ~ Rawlins County (374)
- Kansas ~ Reno County (375)
- Kansas ~ Republic County (376)
- Kansas ~ Rice County (377)
- Kansas ~ Riley County (378)
- Kansas ~ Rooks County (379)
- Kansas ~ Rush County (380)
- Kansas ~ Russell County (381)
- Kansas ~ Saline County (382)
- Kansas ~ Scott County (383)
- Kansas ~ Sedgwick County (384)
- Kansas ~ Seward County (385)
- Kansas ~ Shawnee County (386)
- Kansas ~ Sheridan County (387)
- Kansas ~ Sherman County (388)
- Kansas ~ Smith County (389)
- Kansas ~ Stafford County (390)
- Kansas ~ Stanton County (391)
- Kansas ~ Stevens County (392)
- Kansas ~ Sumner County (393)
- Kansas ~ Thomas County (394)
- Kansas ~ Trego County (395)
- Kansas ~ Wabaunsee County (396)
- Kansas ~ Wallace County (397)
- Kansas ~ Washington County (398)
- Kansas ~ Wichita County (399)
- Kansas ~ Wilson County (400)
- Kansas ~ Woodson County (401)
- Kansas ~ Wyandotte County (402)
- Michigan (403)
- Michigan ~ Alcona County (404)
- Michigan ~ Alger County (405)
- Michigan ~ Allegan County (406)
- Michigan ~ Alpena County (407)
- Michigan ~ Antrim County (408)
- Michigan ~ Arenac County (409)
- Michigan ~ Baraga County (410)
- Michigan ~ Barry County (411)
- Michigan ~ Bay County (412)
- Michigan ~ Benzie County (413)
- Michigan ~ Berrien County (414)
- Michigan ~ Branch County (415)
- Michigan ~ Calhoun County (416)
- Michigan ~ Cass County (417)
- Michigan ~ Charlevoix County (418)
- Michigan ~ Cheboygan County (419)
- Michigan ~ Chippewa County (420)
- Michigan ~ Clare County (421)
- Michigan ~ Clinton County (422)
- Michigan ~ Crawford County (423)
- Michigan ~ Delta County (424)
- Michigan ~ Dickinson County (425)
- Michigan ~ Eaton County (426)
- Michigan ~ Emmet County (427)
- Michigan ~ Genesee County (428)
- Michigan ~ Gladwin County (429)
- Michigan ~ Gogebic County (430)
- Michigan ~ Grand Traverse County (431)
- Michigan ~ Gratiot County (432)
- Michigan ~ Hillsdale County (433)
- Michigan ~ Houghton County (434)
- Michigan ~ Huron County (435)
- Michigan ~ Ingham County (436)
- Michigan ~ Ionia County (437)
- Michigan ~ Iosco County (438)
- Michigan ~ Iron County (439)
- Michigan ~ Isabella County (440)
- Michigan ~ Jackson County (441)
- Michigan ~ Kalamazoo County (442)
- Michigan ~ Kalkaska County (443)
- Michigan ~ Kent County (444)
- Michigan ~ Keweenaw County (445)
- Michigan ~ Lake County (446)
- Michigan ~ Lapeer County (447)
- Michigan ~ Leelanau County (448)
- Michigan ~ Lenawee County (449)
- Michigan ~ Livingston County (450)
- Michigan ~ Luce County (451)
- Michigan ~ Mackinac County (452)
- Michigan ~ Macomb County (453)
- Michigan ~ Manistee County (454)
- Michigan ~ Marquette County (455)
- Michigan ~ Mason County (456)
- Michigan ~ Mecosta County (457)
- Michigan ~ Menominee County (458)
- Michigan ~ Midland County (459)
- Michigan ~ Missaukee County (460)
- Michigan ~ Monroe County (461)
- Michigan ~ Montcalm County (462)
- Michigan ~ Montmorency County (463)
- Michigan ~ Muskegon County (464)
- Michigan ~ Newaygo County (465)
- Michigan ~ Oakland County (466)
- Michigan ~ Oceana County (467)
- Michigan ~ Ogemaw County (468)
- Michigan ~ Ontonagon County (469)
- Michigan ~ Osceola County (470)
- Michigan ~ Oscoda County (471)
- Michigan ~ Otsego County (472)
- Michigan ~ Ottawa County (473)
- Michigan ~ Presque Isle County (474)
- Michigan ~ Roscommon County (475)
- Michigan ~ Saginaw County (476)
- Michigan ~ St. Clair County (477)
- Michigan ~ St. Joseph County (478)
- Michigan ~ Sanilac County (479)
- Michigan ~ Schoolcraft County (480)
- Michigan ~ Shiawassee County (481)
- Michigan ~ Tuscola County (482)
- Michigan ~ Van Buren County (483)
- Michigan ~ Washtenaw County (484)
- Michigan ~ Wayne County (485)
- Michigan ~ Wexford County (486)
- Minnesota (487)
- Minnesota ~ Aitkin County (488)
- Minnesota ~ Anoka County (489)
- Minnesota ~ Becker County (490)
- Minnesota ~ Beltrami County (491)
- Minnesota ~ Benton County (492)
- Minnesota ~ Big Stone County (493)
- Minnesota ~ Blue Earth County (494)
- Minnesota ~ Brown County (495)
- Minnesota ~ Carlton County (496)
- Minnesota ~ Carver County (497)
- Minnesota ~ Cass County (498)
- Minnesota ~ Chippewa County (499)
- Minnesota ~ Chisago County (500)
- Minnesota ~ Clay County (501)
- Minnesota ~ Clearwater County (502)
- Minnesota ~ Cook County (503)
- Minnesota ~ Cottonwood County (504)
- Minnesota ~ Crow Wing County (505)
- Minnesota ~ Dakota County (506)
- Minnesota ~ Dodge County (507)
- Minnesota ~ Douglas County (508)
- Minnesota ~ Faribault County (509)
- Minnesota ~ Fillmore County (510)
- Minnesota ~ Freeborn County (511)
- Minnesota ~ Goodhue County (512)
- Minnesota ~ Grant County (513)
- Minnesota ~ Hennepin County (514)
- Minnesota ~ Houston County (515)
- Minnesota ~ Hubbard County (516)
- Minnesota ~ Isanti County (517)
- Minnesota ~ Itasca County (518)
- Minnesota ~ Jackson County (519)
- Minnesota ~ Kanabec County (520)
- Minnesota ~ Kandiyohi County (521)
- Minnesota ~ Kittson County (522)
- Minnesota ~ Koochiching County (523)
- Minnesota ~ Lac qui Parle County (524)
- Minnesota ~ Lake County (525)
- Minnesota ~ Lake of the Woods County (526)
- Minnesota ~ Le Sueur County (527)
- Minnesota ~ Lincoln County (528)
- Minnesota ~ Lyon County (529)
- Minnesota ~ McLeod County (530)
- Minnesota ~ Mahnomen County (531)
- Minnesota ~ Marshall County (532)
- Minnesota ~ Martin County (533)
- Minnesota ~ Meeker County (534)
- Minnesota ~ Mille Lacs County (535)
- Minnesota ~ Morrison County (536)
- Minnesota ~ Mower County (537)
- Minnesota ~ Murray County (538)
- Minnesota ~ Nicollet County (539)
- Minnesota ~ Nobles County (540)
- Minnesota ~ Norman County (541)
- Minnesota ~ Olmsted County (542)
- Minnesota ~ Otter Tail County (543)
- Minnesota ~ Pennington County (544)
- Minnesota ~ Pine County (545)
- Minnesota ~ Pipestone County (546)
- Minnesota ~ Polk County (547)
- Minnesota ~ Pope County (548)
- Minnesota ~ Ramsey County (549)
- Minnesota ~ Red Lake County (550)
- Minnesota ~ Redwood County (551)
- Minnesota ~ Renville County (552)
- Minnesota ~ Rice County (553)
- Minnesota ~ Rock County (554)
- Minnesota ~ Roseau County (555)
- Minnesota ~ St. Louis County (556)
- Minnesota ~ Scott County (557)
- Minnesota ~ Sherburne County (558)
- Minnesota ~ Sibley County (559)
- Minnesota ~ Stearns County (560)
- Minnesota ~ Steele County (561)
- Minnesota ~ Stevens County (562)
- Minnesota ~ Swift County (563)
- Minnesota ~ Todd County (564)
- Minnesota ~ Traverse County (565)
- Minnesota ~ Wabasha County (566)
- Minnesota ~ Wadena County (567)
- Minnesota ~ Waseca County (568)
- Minnesota ~ Washington County (569)
- Minnesota ~ Watonwan County (570)
- Minnesota ~ Wilkin County (571)
- Minnesota ~ Winona County (572)
- Minnesota ~ Wright County (573)
- Minnesota ~ Yellow Medicine County (574)
- Missouri (575)
- Missouri ~ Adair County (576)
- Missouri ~ Andrew County (577)
- Missouri ~ Atchison County (578)
- Missouri ~ Audrain County (579)
- Missouri ~ Barry County (580)
- Missouri ~ Barton County (581)
- Missouri ~ Bates County (582)
- Missouri ~ Benton County (583)
- Missouri ~ Bollinger County (584)
- Missouri ~ Boone County (585)
- Missouri ~ Buchanan County (586)
- Missouri ~ Butler County (587)
- Missouri ~ Caldwell County (588)
- Missouri ~ Callaway County (589)
- Missouri ~ Camden County (590)
- Missouri ~ Cape Girardeau County (591)
- Missouri ~ Carroll County (592)
- Missouri ~ Carter County (593)
- Missouri ~ Cass County (594)
- Missouri ~ Cedar County (595)
- Missouri ~ Chariton County (596)
- Missouri ~ Christian County (597)
- Missouri ~ Clark County (598)
- Missouri ~ Clay County (599)
- Missouri ~ Clinton County (600)
- Missouri ~ Cole County (601)
- Missouri ~ Cooper County (602)
- Missouri ~ Crawford County (603)
- Missouri ~ Dade County (604)
- Missouri ~ Dallas County (605)
- Missouri ~ Daviess County (606)
- Missouri ~ DeKalb County (607)
- Missouri ~ Dent County (608)
- Missouri ~ Douglas County (609)
- Missouri ~ Dunklin County (610)
- Missouri ~ Franklin County (611)
- Missouri ~ Gasconade County (612)
- Missouri ~ Gentry County (613)
- Missouri ~ Greene County (614)
- Missouri ~ Grundy County (615)
- Missouri ~ Harrison County (616)
- Missouri ~ Henry County (617)
- Missouri ~ Hickory County (618)
- Missouri ~ Holt County (619)
- Missouri ~ Howard County (620)
- Missouri ~ Howell County (621)
- Missouri ~ Iron County (622)
- Missouri ~ Jackson County (623)
- Missouri ~ Jasper County (624)
- Missouri ~ Jefferson County (625)
- Missouri ~ Johnson County (626)
- Missouri ~ Knox County (627)
- Missouri ~ Laclede County (628)
- Missouri ~ Lafayette County (629)
- Missouri ~ Lawrence County (630)
- Missouri ~ Lewis County (631)
- Missouri ~ Lincoln County (632)
- Missouri ~ Linn County (633)
- Missouri ~ Livingston County (634)
- Missouri ~ McDonald County (635)
- Missouri ~ Macon County (636)
- Missouri ~ Madison County (637)
- Missouri ~ Maries County (638)
- Missouri ~ Marion County (639)
- Missouri ~ Mercer County (640)
- Missouri ~ Miller County (641)
- Missouri ~ Mississippi County (642)
- Missouri ~ Moniteau County (643)
- Missouri ~ Monroe County (644)
- Missouri ~ Montgomery County (645)
- Missouri ~ Morgan County (646)
- Missouri ~ New Madrid County (647)
- Missouri ~ Newton County (648)
- Missouri ~ Nodaway County (649)
- Missouri ~ Oregon County (650)
- Missouri ~ Osage County (651)
- Missouri ~ Ozark County (652)
- Missouri ~ Pemiscot County (653)
- Missouri ~ Perry County (654)
- Missouri ~ Pettis County (655)
- Missouri ~ Phelps County (656)
- Missouri ~ Pike County (657)
- Missouri ~ Platte County (658)
- Missouri ~ Polk County (659)
- Missouri ~ Pulaski County (660)
- Missouri ~ Putnam County (661)
- Missouri ~ Ralls County (662)
- Missouri ~ Randolph County (663)
- Missouri ~ Ray County (664)
- Missouri ~ Reynolds County (665)
- Missouri ~ Ripley County (666)
- Missouri ~ St. Charles County (667)
- Missouri ~ St. Clair County (668)
- Missouri ~ Ste. Genevieve County (669)
- Missouri ~ St. Francois County (670)
- Missouri ~ St. Louis County (671)
- Missouri ~ Saline County (672)
- Missouri ~ Schuyler County (673)
- Missouri ~ Scotland County (674)
- Missouri ~ Scott County (675)
- Missouri ~ Shannon County (676)
- Missouri ~ Shelby County (677)
- Missouri ~ Stoddard County (678)
- Missouri ~ Stone County (679)
- Missouri ~ Sullivan County (680)
- Missouri ~ Taney County (681)
- Missouri ~ Texas County (682)
- Missouri ~ Vernon County (683)
- Missouri ~ Warren County (684)
- Missouri ~ Washington County (685)
- Missouri ~ Wayne County (686)
- Missouri ~ Webster County (687)
- Missouri ~ Worth County (688)
- Missouri ~ Wright County (689)
- Missouri ~ City of St. Louis (690)
- Nebraska (691)
- Nebraska ~ Adams County (692)
- Nebraska ~ Antelope County (693)
- Nebraska ~ Arthur County (694)
- Nebraska ~ Banner County (695)
- Nebraska ~ Blaine County (696)
- Nebraska ~ Boone County (697)
- Nebraska ~ Box Butte County (698)
- Nebraska ~ Boyd County (699)
- Nebraska ~ Brown County (700)
- Nebraska ~ Buffalo County (701)
- Nebraska ~ Burt County (702)
- Nebraska ~ Butler County (703)
- Nebraska ~ Cass County (704)
- Nebraska ~ Cedar County (705)
- Nebraska ~ Chase County (706)
- Nebraska ~ Cherry County (707)
- Nebraska ~ Cheyenne County (708)
- Nebraska ~ Clay County (709)
- Nebraska ~ Colfax County (710)
- Nebraska ~ Cuming County (711)
- Nebraska ~ Custer County (712)
- Nebraska ~ Dakota County (713)
- Nebraska ~ Dawes County (714)
- Nebraska ~ Dawson County (715)
- Nebraska ~ Deuel County (716)
- Nebraska ~ Dixon County (717)
- Nebraska ~ Dodge County (718)
- Nebraska ~ Douglas County (719)
- Nebraska ~ Dundy County (720)
- Nebraska ~ Fillmore County (721)
- Nebraska ~ Franklin County (722)
- Nebraska ~ Frontier County (723)
- Nebraska ~ Furnas County (724)
- Nebraska ~ Gage County (725)
- Nebraska ~ Garden County (726)
- Nebraska ~ Garfield County (727)
- Nebraska ~ Gosper County (728)
- Nebraska ~ Grant County (729)
- Nebraska ~ Greeley County (730)
- Nebraska ~ Hall County (731)
- Nebraska ~ Hamilton County (732)
- Nebraska ~ Harlan County (733)
- Nebraska ~ Hayes County (734)
- Nebraska ~ Hitchcock County (735)
- Nebraska ~ Holt County (736)
- Nebraska ~ Hooker County (737)
- Nebraska ~ Howard County (738)
- Nebraska ~ Jefferson County (739)
- Nebraska ~ Johnson County (740)
- Nebraska ~ Kearney County (741)
- Nebraska ~ Keith County (742)
- Nebraska ~ Keya Paha County (743)
- Nebraska ~ Kimball County (744)
- Nebraska ~ Knox County (745)
- Nebraska ~ Lancaster County (746)
- Nebraska ~ Lincoln County (747)
- Nebraska ~ Logan County (748)
- Nebraska ~ Loup County (749)
- Nebraska ~ McPherson County (750)
- Nebraska ~ Madison County (751)
- Nebraska ~ Merrick County (752)
- Nebraska ~ Morrill County (753)
- Nebraska ~ Nance County (754)
- Nebraska ~ Nemaha County (755)
- Nebraska ~ Nuckolls County (756)
- Nebraska ~ Otoe County (757)
- Nebraska ~ Pawnee County (758)
- Nebraska ~ Perkins County (759)
- Nebraska ~ Phelps County (760)
- Nebraska ~ Pierce County (761)
- Nebraska ~ Platte County (762)
- Nebraska ~ Polk County (763)
- Nebraska ~ Red Willow County (764)
- Nebraska ~ Richardson County (765)
- Nebraska ~ Rock County (766)
- Nebraska ~ Saline County (767)
- Nebraska ~ Sarpy County (768)
- Nebraska ~ Saunders County (769)
- Nebraska ~ Scotts Bluff County (770)
- Nebraska ~ Seward County (771)
- Nebraska ~ Sheridan County (772)
- Nebraska ~ Sherman County (773)
- Nebraska ~ Sioux County (774)
- Nebraska ~ Stanton County (775)
- Nebraska ~ Thayer County (776)
- Nebraska ~ Thomas County (777)
- Nebraska ~ Thurston County (778)
- Nebraska ~ Valley County (779)
- Nebraska ~ Washington County (780)
- Nebraska ~ Wayne County (781)
- Nebraska ~ Webster County (782)
- Nebraska ~ Wheeler County (783)
- Nebraska ~ York County (784)
- North Dakota (785)
- North Dakota ~ Adams County (786)
- North Dakota ~ Barnes County (787)
- North Dakota ~ Benson County (788)
- North Dakota ~ Billings County (789)
- North Dakota ~ Bottineau County (790)
- North Dakota ~ Bowman County (791)
- North Dakota ~ Burke County (792)
- North Dakota ~ Burleigh County (793)
- North Dakota ~ Cass County (794)
- North Dakota ~ Cavalier County (795)
- North Dakota ~ Dickey County (796)
- North Dakota ~ Divide County (797)
- North Dakota ~ Dunn County (798)
- North Dakota ~ Eddy County (799)
- North Dakota ~ Emmons County (800)
- North Dakota ~ Foster County (801)
- North Dakota ~ Golden Valley County (802)
- North Dakota ~ Grand Forks County (803)
- North Dakota ~ Grant County (804)
- North Dakota ~ Griggs County (805)
- North Dakota ~ Hettinger County (806)
- North Dakota ~ Kidder County (807)
- North Dakota ~ LaMoure County (808)
- North Dakota ~ Logan County (809)
- North Dakota ~ McHenry County (810)
- North Dakota ~ McIntosh County (811)
- North Dakota ~ McKenzie County (812)
- North Dakota ~ McLean County (813)
- North Dakota ~ Mercer County (814)
- North Dakota ~ Morton County (815)
- North Dakota ~ Mountrail County (816)
- North Dakota ~ Nelson County (817)
- North Dakota ~ Oliver County (818)
- North Dakota ~ Pembina County (819)
- North Dakota ~ Pierce County (820)
- North Dakota ~ Ramsey County (821)
- North Dakota ~ Ransom County (822)
- North Dakota ~ Renville County (823)
- North Dakota ~ Richland County (824)
- North Dakota ~ Rolette County (825)
- North Dakota ~ Sargent County (826)
- North Dakota ~ Sheridan County (827)
- North Dakota ~ Sioux County (828)
- North Dakota ~ Slope County (829)
- North Dakota ~ Stark County (830)
- North Dakota ~ Steele County (831)
- North Dakota ~ Stutsman County (832)
- North Dakota ~ Towner County (833)
- North Dakota ~ Traill County (834)
- North Dakota ~ Walsh County (835)
- North Dakota ~ Ward County (836)
- North Dakota ~ Wells County (837)
- North Dakota ~ Williams County (838)
- Ohio (839)
- Ohio ~ Adams County (840)
- Ohio ~ Allen County (841)
- Ohio ~ Ashland County (842)
- Ohio ~ Ashtabula County (843)
- Ohio ~ Athens County (844)
- Ohio ~ Auglaize County (845)
- Ohio ~ Belmont County (846)
- Ohio ~ Brown County (847)
- Ohio ~ Butler County (848)
- Ohio ~ Carroll County (849)
- Ohio ~ Champaign County (850)
- Ohio ~ Clark County (851)
- Ohio ~ Clermont County (852)
- Ohio ~ Clinton County (853)
- Ohio ~ Columbiana County (854)
- Ohio ~ Coshocton County (855)
- Ohio ~ Crawford County (856)
- Ohio ~ Cuyahoga County (857)
- Ohio ~ Darke County (858)
- Ohio ~ Defiance County (859)
- Ohio ~ Delaware County (860)
- Ohio ~ Erie County (861)
- Ohio ~ Fairfield County (862)
- Ohio ~ Fayette County (863)
- Ohio ~ Franklin County (864)
- Ohio ~ Fulton County (865)
- Ohio ~ Gallia County (866)
- Ohio ~ Geauga County (867)
- Ohio ~ Greene County (868)
- Ohio ~ Guernsey County (869)
- Ohio ~ Hamilton County (870)
- Ohio ~ Hancock County (871)
- Ohio ~ Hardin County (872)
- Ohio ~ Harrison County (873)
- Ohio ~ Henry County (874)
- Ohio ~ Highland County (875)
- Ohio ~ Hocking County (876)
- Ohio ~ Holmes County (877)
- Ohio ~ Huron County (878)
- Ohio ~ Jackson County (879)
- Ohio ~ Jefferson County (880)
- Ohio ~ Knox County (881)
- Ohio ~ Lake County (882)
- Ohio ~ Lawrence County (883)
- Ohio ~ Licking County (884)
- Ohio ~ Logan County (885)
- Ohio ~ Lorain County (886)
- Ohio ~ Lucas County (887)
- Ohio ~ Madison County (888)
- Ohio ~ Mahoning County (889)
- Ohio ~ Marion County (890)
- Ohio ~ Medina County (891)
- Ohio ~ Meigs County (892)
- Ohio ~ Mercer County (893)
- Ohio ~ Miami County (894)
- Ohio ~ Monroe County (895)
- Ohio ~ Montgomery County (896)
- Ohio ~ Morgan County (897)
- Ohio ~ Morrow County (898)
- Ohio ~ Muskingum County (899)
- Ohio ~ Noble County (900)
- Ohio ~ Ottawa County (901)
- Ohio ~ Paulding County (902)
- Ohio ~ Perry County (903)
- Ohio ~ Pickaway County (904)
- Ohio ~ Pike County (905)
- Ohio ~ Portage County (906)
- Ohio ~ Preble County (907)
- Ohio ~ Putnam County (908)
- Ohio ~ Richland County (909)
- Ohio ~ Ross County (910)
- Ohio ~ Sandusky County (911)
- Ohio ~ Scioto County (912)
- Ohio ~ Seneca County (913)
- Ohio ~ Shelby County (914)
- Ohio ~ Stark County (915)
- Ohio ~ Summit County (916)
- Ohio ~ Trumbull County (917)
- Ohio ~ Tuscarawas County (918)
- Ohio ~ Union County (919)
- Ohio ~ Van Wert County (920)
- Ohio ~ Vinton County (921)
- Ohio ~ Warren County (922)
- Ohio ~ Washington County (923)
- Ohio ~ Wayne County (924)
- Ohio ~ Williams County (925)
- Ohio ~ Wood County (926)
- Ohio ~ Wyandot County (927)
- South Dakota (928)
- South Dakota ~ Aurora County (929)
- South Dakota ~ Beadle County (930)
- South Dakota ~ Bennett County (931)
- South Dakota ~ Bon Homme County (932)
- South Dakota ~ Brookings County (933)
- South Dakota ~ Brown County (934)
- South Dakota ~ Brule County (935)
- South Dakota ~ Buffalo County (936)
- South Dakota ~ Butte County (937)
- South Dakota ~ Campbell County (938)
- South Dakota ~ Charles Mix County (939)
- South Dakota ~ Clark County (940)
- South Dakota ~ Clay County (941)
- South Dakota ~ Codington County (942)
- South Dakota ~ Corson County (943)
- South Dakota ~ Custer County (944)
- South Dakota ~ Davison County (945)
- South Dakota ~ Day County (946)
- South Dakota ~ Deuel County (947)
- South Dakota ~ Dewey County (948)
- South Dakota ~ Douglas County (949)
- South Dakota ~ Edmunds County (950)
- South Dakota ~ Fall River County (951)
- South Dakota ~ Faulk County (952)
- South Dakota ~ Grant County (953)
- South Dakota ~ Gregory County (954)
- South Dakota ~ Haakon County (955)
- South Dakota ~ Hamlin County (956)
- South Dakota ~ Hand County (957)
- South Dakota ~ Hanson County (958)
- South Dakota ~ Harding County (959)
- South Dakota ~ Hughes County (960)
- South Dakota ~ Hutchinson County (961)
- South Dakota ~ Hyde County (962)
- South Dakota ~ Jackson County (963)
- South Dakota ~ Jerauld County (964)
- South Dakota ~ Jones County (965)
- South Dakota ~ Kingsbury County (966)
- South Dakota ~ Lake County (967)
- South Dakota ~ Lawrence County (968)
- South Dakota ~ Lincoln County (969)
- South Dakota ~ Lyman County (970)
- South Dakota ~ McCook County (971)
- South Dakota ~ McPherson County (972)
- South Dakota ~ Marshall County (973)
- South Dakota ~ Meade County (974)
- South Dakota ~ Mellette County (975)
- South Dakota ~ Miner County (976)
- South Dakota ~ Minnehaha County (977)
- South Dakota ~ Moody County (978)
- South Dakota ~ Oglala Lakota County (979)
- South Dakota ~ Pennington County (980)
- South Dakota ~ Perkins County (981)
- South Dakota ~ Potter County (982)
- South Dakota ~ Roberts County (983)
- South Dakota ~ Sanborn County (984)
- South Dakota ~ Spink County (985)
- South Dakota ~ Stanley County (986)
- South Dakota ~ Sully County (987)
- South Dakota ~ Todd County (988)
- South Dakota ~ Tripp County (989)
- South Dakota ~ Turner County (990)
- South Dakota ~ Union County (991)
- South Dakota ~ Walworth County (992)
- South Dakota ~ Yankton County (993)
- South Dakota ~ Ziebach County (994)
- Wisconsin (995)
- Wisconsin ~ Adams County (996)
- Wisconsin ~ Ashland County (997)
- Wisconsin ~ Barron County (998)
- Wisconsin ~ Bayfield County (999)
- Wisconsin ~ Brown County (1000)
- Wisconsin ~ Buffalo County (1001)
- Wisconsin ~ Burnett County (1002)
- Wisconsin ~ Calumet County (1003)
- Wisconsin ~ Chippewa County (1004)
- Wisconsin ~ Clark County (1005)
- Wisconsin ~ Columbia County (1006)
- Wisconsin ~ Crawford County (1007)
- Wisconsin ~ Dane County (1008)
- Wisconsin ~ Dodge County (1009)
- Wisconsin ~ Door County (1010)
- Wisconsin ~ Douglas County (1011)
- Wisconsin ~ Dunn County (1012)
- Wisconsin ~ Eau Claire County (1013)
- Wisconsin ~ Florence County (1014)
- Wisconsin ~ Fond du Lac County (1015)
- Wisconsin ~ Forest County (1016)
- Wisconsin ~ Grant County (1017)
- Wisconsin ~ Green County (1018)
- Wisconsin ~ Green Lake County (1019)
- Wisconsin ~ Iowa County (1020)
- Wisconsin ~ Iron County (1021)
- Wisconsin ~ Jackson County (1022)
- Wisconsin ~ Jefferson County (1023)
- Wisconsin ~ Juneau County (1024)
- Wisconsin ~ Kenosha County (1025)
- Wisconsin ~ Kewaunee County (1026)
- Wisconsin ~ La Crosse County (1027)
- Wisconsin ~ Lafayette County (1028)
- Wisconsin ~ Langlade County (1029)
- Wisconsin ~ Lincoln County (1030)
- Wisconsin ~ Manitowoc County (1031)
- Wisconsin ~ Marathon County (1032)
- Wisconsin ~ Marinette County (1033)
- Wisconsin ~ Marquette County (1034)
- Wisconsin ~ Menominee County (1035)
- Wisconsin ~ Milwaukee County (1036)
- Wisconsin ~ Monroe County (1037)
- Wisconsin ~ Oconto County (1038)
- Wisconsin ~ Oneida County (1039)
- Wisconsin ~ Outagamie County (1040)
- Wisconsin ~ Ozaukee County (1041)
- Wisconsin ~ Pepin County (1042)
- Wisconsin ~ Pierce County (1043)
- Wisconsin ~ Polk County (1044)
- Wisconsin ~ Portage County (1045)
- Wisconsin ~ Price County (1046)
- Wisconsin ~ Racine County (1047)
- Wisconsin ~ Richland County (1048)
- Wisconsin ~ Rock County (1049)
- Wisconsin ~ Rusk County (1050)
- Wisconsin ~ St. Croix County (1051)
- Wisconsin ~ Sauk County (1052)
- Wisconsin ~ Sawyer County (1053)
- Wisconsin ~ Shawano County (1054)
- Wisconsin ~ Sheboygan County (1055)
- Wisconsin ~ Taylor County (1056)
- Wisconsin ~ Trempealeau County (1057)
- Wisconsin ~ Vernon County (1058)
- Wisconsin ~ Vilas County (1059)
- Wisconsin ~ Walworth County (1060)
- Wisconsin ~ Washburn County (1061)
- Wisconsin ~ Washington County (1062)
- Wisconsin ~ Waukesha County (1063)
- Wisconsin ~ Waupaca County (1064)
- Wisconsin ~ Waushara County (1065)
- Wisconsin ~ Winnebago County (1066)
- Wisconsin ~ Wood County (1067)

Display This Question:

If My region of residence is: ___________. Select one option from the drop down menu. = West (AK, AZ, CA, CO, HI, ID, MT, NV, NM, OR, UT, WA, WY)

Q1.13 My state and county of residence in the West is:

State (1)

County (2)

- Alaska (1)
- Alaska ~ Aleutians East Borough (2)
- Alaska ~ Municipality of Anchorage (3)
- Alaska ~ Bristol Bay Borough (4)
- Alaska ~ Denali Borough (5)
- Alaska ~ Fairbanks North Star Borough (6)
- Alaska ~ City and Borough of Haines (7)
- Alaska ~ City and Borough of Juneau (8)
- Alaska ~ Kenai Peninsula Borough (9)
- Alaska ~ Ketchikan Gateway Borough (10)
- Alaska ~ Kodiak Island Borough (11)
- Alaska ~ Lake and Peninsula Borough (12)
- Alaska ~ Matanuska-Susitna Borough (13)
- Alaska ~ North Slope Borough (14)
- Alaska ~ Northwest Arctic Borough (15)
- Alaska ~ Petersburg Borough (16)
- Alaska ~ City and Borough of Sitka (17)
- Alaska ~ Municipality of Skagway Borough (18)
- Alaska ~ City and Borough of Wrangell (19)
- Alaska ~ City and Borough of Yakutat (20)
- Alaska ~ Unorganized Borough (21)
- Alaska ~ Aleutians West Census Area (22)
- Alaska ~ Bethel Census Area (23)
- Alaska ~ Dillingham Census Area (24)
- Alaska ~ Hoonah-Angoon Census Area (25)
- Alaska ~ Kusilvak Census Area (26)
- Alaska ~ Nome Census Area (27)
- Alaska ~ Prince of Wales-Hyder Census Area (28)
- Alaska ~ Southeast Fairbanks Census Area (29)
- Alaska ~ Valdez-Cordova Census Area (30)
- Alaska ~ Yukon-Koyukuk Census Area (31)
- Arizona (32)
- Arizona ~ Apache County (33)
- Arizona ~ Cochise County (34)
- Arizona ~ Coconino County (35)
- Arizona ~ Gila County (36)
- Arizona ~ Graham County (37)
- Arizona ~ Greenlee County (38)
- Arizona ~ La Paz County (39)
- Arizona ~ Maricopa County (40)
- Arizona ~ Mohave County (41)
- Arizona ~ Navajo County (42)
- Arizona ~ Pima County (43)
- Arizona ~ Pinal County (44)
- Arizona ~ Santa Cruz County (45)
- Arizona ~ Yavapai County (46)
- Arizona ~ Yuma County (47)
- California (48)
- California ~ Alameda County (49)
- California ~ Alpine County (50)
- California ~ Amador County (51)
- California ~ Butte County (52)
- California ~ Calaveras County (53)
- California ~ Colusa County (54)
- California ~ Contra Costa County (55)
- California ~ Del Norte County (56)
- California ~ El Dorado County (57)
- California ~ Fresno County (58)
- California ~ Glenn County (59)
- California ~ Humboldt County (60)
- California ~ Imperial County (61)
- California ~ Inyo County (62)
- California ~ Kern County (63)
- California ~ Kings County (64)
- California ~ Lake County (65)
- California ~ Lassen County (66)
- California ~ Los Angeles County (67)
- California ~ Madera County (68)
- California ~ Marin County (69)
- California ~ Mariposa County (70)
- California ~ Mendocino County (71)
- California ~ Merced County (72)
- California ~ Modoc County (73)
- California ~ Mono County (74)
- California ~ Monterey County (75)
- California ~ Napa County (76)
- California ~ Nevada County (77)
- California ~ Orange County (78)
- California ~ Placer County (79)
- California ~ Plumas County (80)
- California ~ Riverside County (81)
- California ~ Sacramento County (82)
- California ~ San Benito County (83)
- California ~ San Bernardino County (84)
- California ~ San Diego County (85)
- California ~ City and County of San Francisco (86)
- California ~ San Joaquin County (87)
- California ~ San Luis Obispo County (88)
- California ~ San Mateo County (89)
- California ~ Santa Barbara County (90)
- California ~ Santa Clara County (91)
- California ~ Santa Cruz County (92)
- California ~ Shasta County (93)
- California ~ Sierra County (94)
- California ~ Siskiyou County (95)
- California ~ Solano County (96)
- California ~ Sonoma County (97)
- California ~ Stanislaus County (98)
- California ~ Sutter County (99)
- California ~ Tehama County (100)
- California ~ Trinity County (101)
- California ~ Tulare County (102)
- California ~ Tuolumne County (103)
- California ~ Ventura County (104)
- California ~ Yolo County (105)
- California ~ Yuba County (106)
- Colorado (107)
- Colorado ~ Adams County (108)
- Colorado ~ Alamosa County (109)
- Colorado ~ Arapahoe County (110)
- Colorado ~ Archuleta County (111)
- Colorado ~ Baca County (112)
- Colorado ~ Bent County (113)
- Colorado ~ Boulder County (114)
- Colorado ~ City and County of Broomfield (115)
- Colorado ~ Chaffee County (116)
- Colorado ~ Cheyenne County (117)
- Colorado ~ Clear Creek County (118)
- Colorado ~ Conejos County (119)
- Colorado ~ Costilla County (120)
- Colorado ~ Crowley County (121)
- Colorado ~ Custer County (122)
- Colorado ~ Delta County (123)
- Colorado ~ City and County of Denver (124)
- Colorado ~ Dolores County (125)
- Colorado ~ Douglas County (126)
- Colorado ~ Eagle County (127)
- Colorado ~ Elbert County (128)
- Colorado ~ El Paso County (129)
- Colorado ~ Fremont County (130)
- Colorado ~ Garfield County (131)
- Colorado ~ Gilpin County (132)
- Colorado ~ Grand County (133)
- Colorado ~ Gunnison County (134)
- Colorado ~ Hinsdale County (135)
- Colorado ~ Huerfano County (136)
- Colorado ~ Jackson County (137)
- Colorado ~ Jefferson County (138)
- Colorado ~ Kiowa County (139)
- Colorado ~ Kit Carson County (140)
- Colorado ~ Lake County (141)
- Colorado ~ La Plata County (142)
- Colorado ~ Larimer County (143)
- Colorado ~ Las Animas County (144)
- Colorado ~ Lincoln County (145)
- Colorado ~ Logan County (146)
- Colorado ~ Mesa County (147)
- Colorado ~ Mineral County (148)
- Colorado ~ Moffat County (149)
- Colorado ~ Montezuma County (150)
- Colorado ~ Montrose County (151)
- Colorado ~ Morgan County (152)
- Colorado ~ Otero County (153)
- Colorado ~ Ouray County (154)
- Colorado ~ Park County (155)
- Colorado ~ Phillips County (156)
- Colorado ~ Pitkin County (157)
- Colorado ~ Prowers County (158)
- Colorado ~ Pueblo County (159)
- Colorado ~ Rio Blanco County (160)
- Colorado ~ Rio Grande County (161)
- Colorado ~ Routt County (162)
- Colorado ~ Saguache County (163)
- Colorado ~ San Juan County (164)
- Colorado ~ San Miguel County (165)
- Colorado ~ Sedgwick County (166)
- Colorado ~ Summit County (167)
- Colorado ~ Teller County (168)
- Colorado ~ Washington County (169)
- Colorado ~ Weld County (170)
- Colorado ~ Yuma County (171)
- Hawaii (172)
- Hawaii ~ Hawaii County (173)
- Hawaii ~ City and County of Honolulu (174)
- Hawaii ~ Kalawao County (175)
- Hawaii ~ Kauai County (176)
- Hawaii ~ Maui County (177)
- Idaho (178)
- Idaho ~ Ada County (179)
- Idaho ~ Adams County (180)
- Idaho ~ Bannock County (181)
- Idaho ~ Bear Lake County (182)
- Idaho ~ Benewah County (183)
- Idaho ~ Bingham County (184)
- Idaho ~ Blaine County (185)
- Idaho ~ Boise County (186)
- Idaho ~ Bonner County (187)
- Idaho ~ Bonneville County (188)
- Idaho ~ Boundary County (189)
- Idaho ~ Butte County (190)
- Idaho ~ Camas County (191)
- Idaho ~ Canyon County (192)
- Idaho ~ Caribou County (193)
- Idaho ~ Cassia County (194)
- Idaho ~ Clark County (195)
- Idaho ~ Clearwater County (196)
- Idaho ~ Custer County (197)
- Idaho ~ Elmore County (198)
- Idaho ~ Franklin County (199)
- Idaho ~ Fremont County (200)
- Idaho ~ Gem County (201)
- Idaho ~ Gooding County (202)
- Idaho ~ Idaho County (203)
- Idaho ~ Jefferson County (204)
- Idaho ~ Jerome County (205)
- Idaho ~ Kootenai County (206)
- Idaho ~ Latah County (207)
- Idaho ~ Lemhi County (208)
- Idaho ~ Lewis County (209)
- Idaho ~ Lincoln County (210)
- Idaho ~ Madison County (211)
- Idaho ~ Minidoka County (212)
- Idaho ~ Nez Perce County (213)
- Idaho ~ Oneida County (214)
- Idaho ~ Owyhee County (215)
- Idaho ~ Payette County (216)
- Idaho ~ Power County (217)
- Idaho ~ Shoshone County (218)
- Idaho ~ Teton County (219)
- Idaho ~ Twin Falls County (220)
- Idaho ~ Valley County (221)
- Idaho ~ Washington County (222)
- Montana (223)
- Montana ~ Beaverhead County (224)
- Montana ~ Big Horn County (225)
- Montana ~ Blaine County (226)
- Montana ~ Broadwater County (227)
- Montana ~ Carbon County (228)
- Montana ~ Carter County (229)
- Montana ~ Cascade County (230)
- Montana ~ Chouteau County (231)
- Montana ~ Custer County (232)
- Montana ~ Daniels County (233)
- Montana ~ Dawson County (234)
- Montana ~ Deer Lodge County (235)
- Montana ~ Fallon County (236)
- Montana ~ Fergus County (237)
- Montana ~ Flathead County (238)
- Montana ~ Gallatin County (239)
- Montana ~ Garfield County (240)
- Montana ~ Glacier County (241)
- Montana ~ Golden Valley County (242)
- Montana ~ Granite County (243)
- Montana ~ Hill County (244)
- Montana ~ Jefferson County (245)
- Montana ~ Judith Basin County (246)
- Montana ~ Lake County (247)
- Montana ~ Lewis and Clark County (248)
- Montana ~ Liberty County (249)
- Montana ~ Lincoln County (250)
- Montana ~ McCone County (251)
- Montana ~ Madison County (252)
- Montana ~ Meagher County (253)
- Montana ~ Mineral County (254)
- Montana ~ Missoula County (255)
- Montana ~ Musselshell County (256)
- Montana ~ Park County (257)
- Montana ~ Petroleum County (258)
- Montana ~ Phillips County (259)
- Montana ~ Pondera County (260)
- Montana ~ Powder River County (261)
- Montana ~ Powell County (262)
- Montana ~ Prairie County (263)
- Montana ~ Ravalli County (264)
- Montana ~ Richland County (265)
- Montana ~ Roosevelt County (266)
- Montana ~ Rosebud County (267)
- Montana ~ Sanders County (268)
- Montana ~ Sheridan County (269)
- Montana ~ Silver Bow County (270)
- Montana ~ Stillwater County (271)
- Montana ~ Sweet Grass County (272)
- Montana ~ Teton County (273)
- Montana ~ Toole County (274)
- Montana ~ Treasure County (275)
- Montana ~ Valley County (276)
- Montana ~ Wheatland County (277)
- Montana ~ Wibaux County (278)
- Montana ~ Yellowstone County (279)
- Nevada (280)
- Nevada ~ Churchill County (281)
- Nevada ~ Clark County (282)
- Nevada ~ Douglas County (283)
- Nevada ~ Elko County (284)
- Nevada ~ Esmeralda County (285)
- Nevada ~ Eureka County (286)
- Nevada ~ Humboldt County (287)
- Nevada ~ Lander County (288)
- Nevada ~ Lincoln County (289)
- Nevada ~ Lyon County (290)
- Nevada ~ Mineral County (291)
- Nevada ~ Nye County (292)
- Nevada ~ Pershing County (293)
- Nevada ~ Storey County (294)
- Nevada ~ Washoe County (295)
- Nevada ~ White Pine County (296)
- Nevada ~ Consolidated Municipality of Carson City (297)
- New Mexico (298)
- New Mexico ~ Bernalillo County (299)
- New Mexico ~ Catron County (300)
- New Mexico ~ Chaves County (301)
- New Mexico ~ Cibola County (302)
- New Mexico ~ Colfax County (303)
- New Mexico ~ Curry County (304)
- New Mexico ~ De Baca County (305)
- New Mexico ~ Doña Ana County (306)
- New Mexico ~ Eddy County (307)
- New Mexico ~ Grant County (308)
- New Mexico ~ Guadalupe County (309)
- New Mexico ~ Harding County (310)
- New Mexico ~ Hidalgo County (311)
- New Mexico ~ Lea County (312)
- New Mexico ~ Lincoln County (313)
- New Mexico ~ Municipality and County of Los Alamos (314)
- New Mexico ~ Luna County (315)
- New Mexico ~ McKinley County (316)
- New Mexico ~ Mora County (317)
- New Mexico ~ Otero County (318)
- New Mexico ~ Quay County (319)
- New Mexico ~ Rio Arriba County (320)
- New Mexico ~ Roosevelt County (321)
- New Mexico ~ Sandoval County (322)
- New Mexico ~ San Juan County (323)
- New Mexico ~ San Miguel County (324)
- New Mexico ~ Santa Fe County (325)
- New Mexico ~ Sierra County (326)
- New Mexico ~ Socorro County (327)
- New Mexico ~ Taos County (328)
- New Mexico ~ Torrance County (329)
- New Mexico ~ Union County (330)
- New Mexico ~ Valencia County (331)
- Oregon (332)
- Oregon ~ Baker County (333)
- Oregon ~ Benton County (334)
- Oregon ~ Clackamas County (335)
- Oregon ~ Clatsop County (336)
- Oregon ~ Columbia County (337)
- Oregon ~ Coos County (338)
- Oregon ~ Crook County (339)
- Oregon ~ Curry County (340)
- Oregon ~ Deschutes County (341)
- Oregon ~ Douglas County (342)
- Oregon ~ Gilliam County (343)
- Oregon ~ Grant County (344)
- Oregon ~ Harney County (345)
- Oregon ~ Hood River County (346)
- Oregon ~ Jackson County (347)
- Oregon ~ Jefferson County (348)
- Oregon ~ Josephine County (349)
- Oregon ~ Klamath County (350)
- Oregon ~ Lake County (351)
- Oregon ~ Lane County (352)
- Oregon ~ Lincoln County (353)
- Oregon ~ Linn County (354)
- Oregon ~ Malheur County (355)
- Oregon ~ Marion County (356)
- Oregon ~ Morrow County (357)
- Oregon ~ Multnomah County (358)
- Oregon ~ Polk County (359)
- Oregon ~ Sherman County (360)
- Oregon ~ Tillamook County (361)
- Oregon ~ Umatilla County (362)
- Oregon ~ Union County (363)
- Oregon ~ Wallowa County (364)
- Oregon ~ Wasco County (365)
- Oregon ~ Washington County (366)
- Oregon ~ Wheeler County (367)
- Oregon ~ Yamhill County (368)
- Utah (369)
- Utah ~ Beaver County (370)
- Utah ~ Box Elder County (371)
- Utah ~ Cache County (372)
- Utah ~ Carbon County (373)
- Utah ~ Daggett County (374)
- Utah ~ Davis County (375)
- Utah ~ Duchesne County (376)
- Utah ~ Emery County (377)
- Utah ~ Garfield County (378)
- Utah ~ Grand County (379)
- Utah ~ Iron County (380)
- Utah ~ Juab County (381)
- Utah ~ Kane County (382)
- Utah ~ Millard County (383)
- Utah ~ Morgan County (384)
- Utah ~ Piute County (385)
- Utah ~ Rich County (386)
- Utah ~ Salt Lake County (387)
- Utah ~ San Juan County (388)
- Utah ~ Sanpete County (389)
- Utah ~ Sevier County (390)
- Utah ~ Summit County (391)
- Utah ~ Tooele County (392)
- Utah ~ Uintah County (393)
- Utah ~ Utah County (394)
- Utah ~ Wasatch County (395)
- Utah ~ Washington County (396)
- Utah ~ Wayne County (397)
- Utah ~ Weber County (398)
- Washington (399)
- Washington ~ Adams County (400)
- Washington ~ Asotin County (401)
- Washington ~ Benton County (402)
- Washington ~ Chelan County (403)
- Washington ~ Clallam County (404)
- Washington ~ Clark County (405)
- Washington ~ Columbia County (406)
- Washington ~ Cowlitz County (407)
- Washington ~ Douglas County (408)
- Washington ~ Ferry County (409)
- Washington ~ Franklin County (410)
- Washington ~ Garfield County (411)
- Washington ~ Grant County (412)
- Washington ~ Grays Harbor County (413)
- Washington ~ Island County (414)
- Washington ~ Jefferson County (415)
- Washington ~ King County (416)
- Washington ~ Kitsap County (417)
- Washington ~ Kittitas County (418)
- Washington ~ Klickitat County (419)
- Washington ~ Lewis County (420)
- Washington ~ Lincoln County (421)
- Washington ~ Mason County (422)
- Washington ~ Okanogan County (423)
- Washington ~ Pacific County (424)
- Washington ~ Pend Oreille County (425)
- Washington ~ Pierce County (426)
- Washington ~ San Juan County (427)
- Washington ~ Skagit County (428)
- Washington ~ Skamania County (429)
- Washington ~ Snohomish County (430)
- Washington ~ Spokane County (431)
- Washington ~ Stevens County (432)
- Washington ~ Thurston County (433)
- Washington ~ Wahkiakum County (434)
- Washington ~ Walla Walla County (435)
- Washington ~ Whatcom County (436)
- Washington ~ Whitman County (437)
- Washington ~ Yakima County (438)
- Wyoming (439)
- Wyoming ~ Albany County (440)
- Wyoming ~ Big Horn County (441)
- Wyoming ~ Campbell County (442)
- Wyoming ~ Carbon County (443)
- Wyoming ~ Converse County (444)
- Wyoming ~ Crook County (445)
- Wyoming ~ Fremont County (446)
- Wyoming ~ Goshen County (447)
- Wyoming ~ Hot Springs County (448)
- Wyoming ~ Johnson County (449)
- Wyoming ~ Laramie County (450)
- Wyoming ~ Lincoln County (451)
- Wyoming ~ Natrona County (452)
- Wyoming ~ Niobrara County (453)
- Wyoming ~ Park County (454)
- Wyoming ~ Platte County (455)
- Wyoming ~ Sheridan County (456)
- Wyoming ~ Sublette County (457)
- Wyoming ~ Sweetwater County (458)
- Wyoming ~ Teton County (459)
- Wyoming ~ Uinta County (460)
- Wyoming ~ Washakie County (461)
- Wyoming ~ Weston County (462)

|  |
| --- |

Q1.14 Do you or anyone in your household have a pet now, or have you had a pet in the last 2 years?

- Yes, my household has at least one pet currently (1)
- Yes, my household has had a pet in the past 2 years but not currently (2)
- No, my household has not had a pet at any point in the last 2 years (3)

Q1.15 Are you currently employed, had any form of employment or worked for pay during the past 2 years?  


Please answer yes if you are full-time, part-time, or have been occasionally employed at any point in the past 2 years.

- Yes (1)
- No (2)

Q1.16 What best describes your primary living situation? (please select all that apply)

- Own a house (1)
- Own an apartment (2)
- Rent a house (3)
- Rent an apartment (4)
- Live with extended family members in the same house or apartment (5)
- Live with unrelated individuals (e.g. roommates or shared living arrangements) (6)
- None of the options provided apply to me (7)

|  |
| --- |

Q2.1 Please select the bracket that most closely represents your own personal income. 


Note that earlier you reported ${Q1.7/ChoiceGroup/SelectedChoices} in response to the request for total household income.  
You are now being asked only for your own annual income which may be identical to household income or may be a portion of the total household income reported.

- Less than $10,000 annually (1)
- $10,000 - $14,999 (2)
- $15,000 - $19,999 (3)
- $20,000 - $24,999 (4)
- $25,000 - $29,999 (5)
- $30,000 - $34,999 (6)
- $35,000 - $39,999 (7)
- $40,000 - $44,999 (8)
- $45,000 - $49,999 (9)
- $50,000 - $54,999 (10)
- $55,000 - $59,999 (11)
- $60,000 - $64,999 (12)
- $65,000 - $69,999 (13)
- $70,000 - $74,999 (14)
- $75,000 - $79,999 (15)
- $80,000 - $84,999 (16)
- $85,000 - $89,999 (17)
- $90,000 - $94,999 (18)
- $95,000 - $99,999 (19)
- $100,000 - $104,999 (20)
- $105,000 - $109,999 (21)
- $110,000 - $114,999 (22)
- $115,000 - $119,999 (23)
- $120,000 - $124,999 (24)
- $125,000 - $129,999 (25)
- $130,000 - $134,999 (26)
- $135,000 - $139,999 (27)
- $140,000 - $144,999 (28)
- $145,000 - $149,999 (29)
- More than $150,000 annually (30)

Q2.2 Please indicate your work locations for the time periods outlined:

|  | I worked from home | | | I worked in an office or other place of employment | | | I have a physical place of employment (i.e. office) but am able to work remotely | | |
| --- | --- | --- | --- | --- | --- | --- | --- | --- | --- |
|  | Never (1) | Sometimes (2) | Always (3) | Never (1) | Sometimes (2) | Always (3) | Never (1) | Sometimes (2) | Always (3) |
| Before March 2020 (1) |  |  |  |  |  |  |  |  |  |
| March 2020 - May 2020 (2) |  |  |  |  |  |  |  |  |  |
| June 2020 - August 2020 (4) |  |  |  |  |  |  |  |  |  |
| September 2020 - December 2020 (5) |  |  |  |  |  |  |  |  |  |
| January 2021 - May 2021 (6) |  |  |  |  |  |  |  |  |  |
| June 2021 - August 2021 (7) |  |  |  |  |  |  |  |  |  |
| September 2021 - Today (8) |  |  |  |  |  |  |  |  |  |

Q2.3 Is your job/employment capable of being done remotely?

- Yes (1)
- Sometimes/Partially (2)
- No (3)

Q2.4 My ideal work location would be:

- working exclusively in office or on physical work site (1)
- working 'hybrid' with both remote and on-site capabilities (2)
- working exclusively remotely (3)

Q2.5 Would you consider leaving your current position to obtain your preferred work environment/location?

- Yes (1)
- Maybe (2)
- No (3)
- Not applicable (4)

Q2.6 Would you consider taking a reduction in annual salary to obtain flexibility in work location, either at your current position/job or via obtaining a different position?

- Yes (1)
- No (2)

Display This Question:

If Would you consider taking a reduction in annual salary to obtain flexibility in work location, ei... = Yes

|  |
| --- |

Q2.7 What is the maximum reduction in annual salary that you would accept in exchange for having your ideal work location, in terms of on-site versus hybrid versus remote work?

- I would give up a maximum of 1% of my annual salary (1)
- I would give up a maximum of 2% of my annual salary (2)
- I would give up a maximum of 3% of my annual salary (3)
- I would give up a maximum of 4% of my annual salary (4)
- I would give up a maximum of 5% of my annual salary (5)
- I would give up a maximum of 6% of my annual salary (6)
- I would give up a maximum of 7% of my annual salary (7)
- I would give up a maximum of 8% of my annual salary (8)
- I would give up a maximum of 9% of my annual salary (9)
- I would give up a maximum of 10% of my annual salary (10)
- I would give up a maximum of 11% of my annual salary (11)
- I would give up a maximum of 12% of my annual salary (12)
- I would give up a maximum of 13% of my annual salary (13)
- I would give up a maximum of 14% of my annual salary (14)
- I would give up a maximum of 15% of my annual salary (15)
- I would give up a maximum of 16% of my annual salary (16)
- I would give up a maximum of 17% of my annual salary (17)
- I would give up a maximum of 18% of my annual salary (18)
- I would give up a maximum of 19% of my annual salary (19)
- I would give up a maximum of 20% of my annual salary (20)
- I would give up a maximum of 21% of my annual salary (21)
- I would give up a maximum of 22% of my annual salary (22)
- I would give up a maximum of 23% of my annual salary (23)
- I would give up a maximum of 24% of my annual salary (24)
- I would give up a maximum of 25% of my annual salary (25)
- I would give up a maximum of 50% of my annual salary (26)
- I would give up a maximum of 75% of my annual salary (27)
- I would give up more than 75% of my annual salary (28)
- 3 (29)

Q3.1 The following FOURTEEN questions present you with one of eight total reasons that one may consider important reasons for desiring to work remotely or from home. 


**The eight reasons include, in no particular order,**


- Saving money on lifestyle choices, such as eating lunches out
- Saving or reallocating commuting time and expenses
- Desire to reduce viral/disease exposure for self and/or household members 
- Facilitating balance of work with caregiving responsibilities for children, family members, and/or pets
- More productive at home (i.e. less distractions) 
- Prefer the home environment 
- Simplified or lessened grooming or dressing cost, in time and/or money, to work from home or remotely
- Prefer ability to intermingle work and home activities (i.e. waiting for plumber or electrician while conducting meetings or working from home simultaneously) 

For each of the FOURTEEN total question, please select the reason you believe is the most important and the reason you believe is the least important from the given options. Please note that you must select a most and a least important reason option from each of the FOURTEEN questions presented.

Q3.2 Choice 1 out of 14: From the following options, please select the most important and least important reasons for working remotely or from home.

| Most Important (1) |  | Least Important (2) |
| --- | --- | --- |
|  | Facilitating balance of work with caregiving responsibilities for children, family members, and/or pets (1) |  |
|  | Prefer ability to intermingle work and home activities (2) |  |
|  | Saving money on lifestyle choices, such as eating lunches out (3) |  |
|  | Prefer the home environment (4) |  |

Q3.3 Choice 2 out of 14: From the following options, please select the most important and least important reasons for working remotely or from home.

| Most Important (1) |  | Least Important (2) |
| --- | --- | --- |
|  | Simplified or lessened grooming or dressing cost, in time and/or money, to work from home or remotely (1) |  |
|  | Facilitating balance of work with caregiving responsibilities for children, family members, and/or pets (2) |  |
|  | More productive at home (3) |  |
|  | Prefer ability to intermingle work and home activities (4) |  |

Q3.4 Choice 3 out of 14: From the following options, please select the most important and least important reasons for working remotely or from home.

| Most Important (1) |  | Least Important (2) |
| --- | --- | --- |
|  | Prefer the home environment (1) |  |
|  | Desire to reduce viral/disease exposure for self and/or household members (2) |  |
|  | Saving money on lifestyle choices, such as eating lunches out (3) |  |
|  | Saving or reallocating commuting time and expense (4) |  |

Q3.5 Choice 4 out of 14: From the following options, please select the most important and least important reasons for working remotely or from home.

| Most Important (1) |  | Least Important (2) |
| --- | --- | --- |
|  | Saving or reallocating commuting time and expense (1) |  |
|  | Saving money on lifestyle choices, such as eating lunches out (2) |  |
|  | Prefer ability to intermingle work and home activities (3) |  |
|  | More productive at home (4) |  |

Q3.6 Choice 5 out of 14: From the following options, please select the most important and least important reasons for working remotely or from home.

| Most Important (1) |  | Least Important (2) |
| --- | --- | --- |
|  | Saving or reallocating commuting time and expense (1) |  |
|  | Facilitating balance of work with caregiving responsibilities for children, family members, and/or pets (2) |  |
|  | More productive at home (3) |  |
|  | Saving money on lifestyle choices, such as eating lunches out (4) |  |

Q3.7 Choice 6 out of 14: From the following options, please select the most important and least important reasons for working remotely or from home.

| Most Important (1) |  | Least Important (2) |
| --- | --- | --- |
|  | Facilitating balance of work with caregiving responsibilities for children, family members, and/or pets (1) |  |
|  | More productive at home (2) |  |
|  | Prefer the home environment (3) |  |
|  | Desire to reduce viral/disease exposure for self and/or household members (4) |  |

Q3.8 Choice 7 out of 14: From the following options, please select the most important and least important reasons for working remotely or from home.

| Most Important (1) |  | Least Important (2) |
| --- | --- | --- |
|  | Prefer the home environment (1) |  |
|  | Saving or reallocating commuting time and expense (2) |  |
|  | Facilitating balance of work with caregiving responsibilities for children, family members, and/or pets (3) |  |
|  | Simplified or lessened grooming or dressing cost, in time and/or money, to work from home or remotely (4) |  |

Q3.9 Choice 8 out of 14: From the following options, please select the most important and least important reasons for working remotely or from home.

| Most Important (1) |  | Least Important (2) |
| --- | --- | --- |
|  | Saving money on lifestyle choices, such as eating lunches out (1) |  |
|  | Simplified or lessened grooming or dressing cost, in time and/or money, to work from home or remotely (2) |  |
|  | Desire to reduce viral/disease exposure for self and/or household members (3) |  |
|  | Prefer ability to intermingle work and home activities (4) |  |

Q3.10 Choice 9 out of 14: From the following options, please select the most important and least important reasons for working remotely or from home.

| Most Important (1) |  | Least Important (2) |
| --- | --- | --- |
|  | Prefer ability to intermingle work and home activities (1) |  |
|  | Prefer the home environment (2) |  |
|  | Simplified or lessened grooming or dressing cost, in time and/or money, to work from home or remotely (3) |  |
|  | Saving or reallocating commuting time and expense (4) |  |

Q3.11 Choice 10 out of 14: From the following options, please select the most important and least important reasons for working remotely or from home.

| Most Important (1) |  | Least Important (2) |
| --- | --- | --- |
|  | More productive at home (1) |  |
|  | Saving money on lifestyle choices, such as eating lunches out (2) |  |
|  | Prefer the home environment (3) |  |
|  | Simplified or lessened grooming or dressing cost, in time and/or money, to work from home or remotely (4) |  |

Q3.12 Choice 11 out of 14: From the following options, please select the most important and least important reasons for working remotely or from home.

| Most Important (1) |  | Least Important (2) |
| --- | --- | --- |
|  | Simplified or lessened grooming or dressing cost, in time and/or money, to work from home or remotely (1) |  |
|  | More productive at home (2) |  |
|  | Saving or reallocating commuting time and expense (3) |  |
|  | Desire to reduce viral/disease exposure for self and/or household members (4) |  |

Q3.13 Choice 12 out of 14: From the following options, please select the most important and least important reasons for working remotely or from home.

| Most Important (1) |  | Least Important (2) |
| --- | --- | --- |
|  | Desire to reduce viral/disease exposure for self and/or household members (1) |  |
|  | Prefer the home environment (2) |  |
|  | Prefer ability to intermingle work and home activities (3) |  |
|  | More productive at home (4) |  |

Q3.14 Choice 13 out of 14: From the following options, please select the most important and least important reasons for working remotely or from home.

| Most Important (1) |  | Least Important (2) |
| --- | --- | --- |
|  | Desire to reduce viral/disease exposure for self and/or household members (1) |  |
|  | Simplified or lessened grooming or dressing cost, in time and/or money, to work from home or remotely (2) |  |
|  | Facilitating balance of work with caregiving responsibilities for children, family members, and/or pets (3) |  |
|  | Saving money on lifestyle choices, such as eating lunches out (4) |  |

Q3.15 Choice 14 out of 14: From the following options, please select the most important and least important reasons for working remotely or from home.

| Most Important (1) |  | Least Important (2) |
| --- | --- | --- |
|  | Prefer ability to intermingle work and home activities (1) |  |
|  | Desire to reduce viral/disease exposure for self and/or household members (2) |  |
|  | Saving or reallocating commuting time and expense (3) |  |
|  | Facilitating balance of work with caregiving responsibilities for children, family members, and/or pets (4) |  |

Q4.1 Please select the number of each animal you have had in your household in the past 2 years:

|  | 0 (1) | 1 (2) | 2 (3) | 3 (4) | 4 or more (5) |
| --- | --- | --- | --- | --- | --- |
| Dogs (1) |  |  |  |  |  |
| Cats (2) |  |  |  |  |  |
| Horses (3) |  |  |  |  |  |
| Fish (4) |  |  |  |  |  |
| Birds (5) |  |  |  |  |  |
| Reptiles (6) |  |  |  |  |  |
| Small mammals (Mice, Guinea Pigs, Rabbit, Hamster, Rat, Chinchilla, Ferrets, Gerbil, etc.) (7) |  |  |  |  |  |

Q4.2 Have you experienced any of the following during the COVID-19 pandemic time period regarding your pets?

|  | Yes (1) | No (2) | Does Not Apply (3) |
| --- | --- | --- | --- |
| Increased needs for training due to isolation or lack of socialization (1) |  |  |  |
| Increased separation anxiety when leaving pets at home (2) |  |  |  |
| Difficulty accessing basic veterinary care, including vaccinations and/or annual exams (3) |  |  |  |
| Difficulty accessing specialty veterinary care (4) |  |  |  |
| Extended waits for veterinary care appointments (5) |  |  |  |

Q4.3 Have you obtained a new pet during the COVID-19 pandemic time period (between March 2020 and present day)?

- Yes (1)
- No (2)

Q4.4 What type of pet(s) did you obtain? (Check all that apply)

- One dog (1)
- Two dogs (2)
- Three or more dogs (3)
- One cat (4)
- Two cats (5)
- Three or more cats (6)
- One or more fish (7)
- One or more small caged pets (ie. hamsters, turtles, etc.) (8)
- Other, please specify (9) ________________________________________________

Q4.5 How did you acquire your pet? (Check all that apply)

- Adoption (shelter or rescue organization) (1)
- Bred them myself (2)
- Purchased from a breeder (3)
- Purchased from pet store (4)
- Stray (5)
- Gift from family member/friend (6)
- Other, please specify (7) ________________________________________________

Q4.6 For what reasons did you acquire your pet in that manner? (Select all that apply)

- Impulse buy (1)
- Reputation of the breeder (2)
- Reputation of the rescue/shelter (3)
- Previous experience (4)
- Wanted a specific breed or type of pet (5)
- Cost (6)
- Guilt (7)
- Peer pressure (8)
- It was the right thing to do (9)
- Came with pet insurance (10)
- Came with training/education (11)
- Came with health guarantee (12)

Q4.7 Were you able to find and obtain the pet you wanted during the Covid-era? (Select all that apply)

- Yes, I was able to access the pet I wanted (1)
- I did not have a specific pet in mind (2)
- I would have made a different choice if there was greater availability/access (3)
- Other (4)

Q4.8 Have you kept and/or are you planning on keeping your new pet?

- I have already given away or otherwise rehomed my new pet (1)
- My new pet has passed away (2)
- Yes, I plan on keeping my new pet (3)
- No, I plan on rehoming or giving away my new pet in the future (4)
- Other (5)

Display This Question:

If Have you kept and/or are you planning on keeping your new pet? = I have already given away or otherwise rehomed my new pet

Or Have you kept and/or are you planning on keeping your new pet? = No, I plan on rehoming or giving away my new pet in the future

|  |
| --- |

Q4.9 Why have you relinquished your pet or are you considering relinquishing your pet?

- Behavioral difficulties (1)
- Health difficulties (2)
- Too costly (3)
- Cannot manage necessary pet care (4)
- Other (5)

Q5.1 How would you evaluate your everyday life in the Fall of 2021 relative to pre-pandemic (pre-March 2020 times) 'normal'?

- Everyday Life Has Returned to Normal (1)
- Everyday Life Has Not Returned to Normal (2)

Q5.2 Are you and your household members vaccinated for COVID-19?

|  | Fully Vaccinated (1) | Partially Vaccinated (2) | Some individuals are at least in-part vaccinated but some are not (3) | No, Not Vaccinated (4) | Choose Not to Answer (5) | Does Not Apply (6) |
| --- | --- | --- | --- | --- | --- | --- |
| You/yourself (1) |  |  |  |  |  |  |
| Other adult(s) in your household (2) |  |  |  |  |  |  |
| Children over 12 years of age (3) |  |  |  |  |  |  |
| Children 5 - 11 years of age (4) |  |  |  |  |  |  |
| Children under 5 years of age (5) |  |  |  |  |  |  |

Q5.3 Have you or your family experienced challenges in obtaining items due to supply chain issues or product shortages in the Fall of 2021? (Select all that apply.)

- Yes, we have had trouble getting durable household goods (i.e. washing machine, freezer, dryer, other appliances and durables) (1)
- Yes, we have had trouble getting household electronics (2)
- Yes, we have had trouble getting car/truck parts for personal vehicles (3)
- Yes, we have had trouble getting certain food items (4)
- Yes, we have had trouble getting household paper goods and supplies (i.e. toilet paper or paper towels) (5)
- Yes, we have had trouble getting other items, please specify (6) ________________________________________________
- No, we have not had any trouble getting items (7)

Q5.4 Are you or members of your household experiencing shipping/delivery delays or challenges in the August 2021 to Current Day time period and/or are you concerned about shipping/delivery delays in the upcoming holiday season? (Select all that apply.)

- Yes, I/we have experienced shipping delays or concerns (1)
- Yes, I/we are concerned about upcoming shipping delays or concerns (2)
- I/we do not have concerns but I have heard about or am aware of delays/challenges more generally (3)
- I/we have no concerns and I am not aware of delays/challenges more generally (4)

Q5.5 How have your spending habits changed comparing the time period of Fall 2021 and the pre-pandemic time period? 


Compared to pre-March 2020 (Pre-Covid), my spending in Fall 2021 is:

|  | Less (1) | About the Same (2) | More (3) | Not Applicable/Have Never Spent on This (4) |
| --- | --- | --- | --- | --- |
| Suits, dresses, or professional clothing (1) |  |  |  |  |
| Casual clothing (2) |  |  |  |  |
| Professional hair services (i.e. color, cuts, professional styling) (3) |  |  |  |  |
| Professional nail services (4) |  |  |  |  |
| To-Go or On-The-Go coffee or other beverages (5) |  |  |  |  |
| Makeup (6) |  |  |  |  |
| Hair care products (i.e. shampoo/conditioner) (7) |  |  |  |  |
| At-home personal care items (i.e. soaps or at-home treatments) (8) |  |  |  |  |
| Professional spa or spa-like services (9) |  |  |  |  |
| Personal Protective Equipment (i.e. face coverings, masks) (11) |  |  |  |  |
| Eating in restaurants (12) |  |  |  |  |
| Take out food items (13) |  |  |  |  |
| Travel (14) |  |  |  |  |
| Work-related commuting expenses (15) |  |  |  |  |

Display This Question:

If How have your spending habits changed comparing the time period of Fall 2021 and the pre-pandemic... = Suits, dresses, or professional clothing [ More ]

|  |  |
| --- | --- |

Q5.6 Why have you spent more on suits, dresses, or professional clothing in the Fall of 2021? (Select all that apply.)

- Existing wardrobe no longer fits because it was too tight/small (1)
- Existing wardrobe no longer fits because it was too loose/big (2)
- Wanted to update clothing options (3)
- Style/preferences have changed (4)
- Needed clothing that I did not previously need or have (5)
- Back to in-person work spending (6)
- Back to in-person school spending (7)
- No real reason (8)
- Other (9) ________________________________________________

Display This Question:

If How have your spending habits changed comparing the time period of Fall 2021 and the pre-pandemic... = Casual clothing [ More ]

|  |  |
| --- | --- |

Q5.7 Why have you spent more on casual clothing in the Fall of 2021? (Select all that apply.)

- Existing wardrobe no longer fit because it was too tight/small (1)
- Existing wardrobe no longer fit because it was too loose/big (2)
- Wanted to update clothing options (3)
- Style/preferences have changed (4)
- Needed clothing that I did not previously need or have (5)
- Back to in-person work spending (6)
- Back to in-person school spending (7)
- No real reason (8)
- Other (9) ________________________________________________

Display This Question:

If How have your spending habits changed comparing the time period of Fall 2021 and the pre-pandemic... = Professional hair services (i.e. color, cuts, professional styling) [ More ]

Or How have your spending habits changed comparing the time period of Fall 2021 and the pre-pandemic... = Professional nail services [ More ]

Or How have your spending habits changed comparing the time period of Fall 2021 and the pre-pandemic... = Makeup [ More ]

Or How have your spending habits changed comparing the time period of Fall 2021 and the pre-pandemic... = Hair care products (i.e. shampoo/conditioner) [ More ]

Or How have your spending habits changed comparing the time period of Fall 2021 and the pre-pandemic... = At-home personal care items (i.e. soaps or at-home treatments) [ More ]

|  |  |
| --- | --- |

Q5.8 Why have you spent more on hair, nail, and/or other personal care in the Fall of 2021? (Select all that apply.)

- I'm buying less but more expensive products/services (1)
- I am replacing items worn out or running low/out (2)
- I am now buying items/services that I had cut down on during the pandemic era (3)
- Changes in spending are entirely non-pandemic related (4)
- I am beginning to invest in personal care more now as I go out more often than I have in the past 16-18 months (5)
- Back to in-person work spending (6)
- Back to in-person school spending (7)
- Other (8) ________________________________________________

Q5.9 Have you experienced any of the following life experiences in the time period between March 2020 and today? (Select all that you have experienced.) 


I have experienced:

- Death in the family (1)
- COVID-19 related death in the family (2)
- Marriage (3)
- Divorce (4)
- Forced loss of job or employment (5)
- Forced reduction in hours of employment (6)
- Voluntarily quitting a job (7)
- Starting a new job (8)
- Moving personal residence/home (9)
- Serious illness of household or immediate family member (10)
- Serious COVID-19 related illness in household or immediate family member (11)
- Serious COVID-19 related illness in extended family (12)
- Hospitalization for any reason (13)
- Birth of baby (14)

Q5.10 Please indicate your level of agreement with the following changes in behavior since March 2020, or during the pandemic-era.

|  | Agree (1) | Somewhat or Sometimes Agree (2) | Do Not Agree (3) | Don't Know or Does Not Apply (4) |
| --- | --- | --- | --- | --- |
| I spend less time on personal care now than I did pre-March 2020 (1) |  |  |  |  |
| I spend less time on my appearance overall now than I did in pre-March 2020 (2) |  |  |  |  |
| I care less about what clothes I wear now than I did pre-March 2020 (3) |  |  |  |  |
| I wash my hair less often now than I did pre-March 2020 (4) |  |  |  |  |
| I shower less often now than I did pre-March 2020 (5) |  |  |  |  |

Q6.1 Approximately how far do you live from your nearest family member?

- Less than 15 miles (1)
- 16 miles to 250 miles (2)
- 251 miles to 500 miles (3)
- more than 500 miles (4)

Q78 Has your experience during the COVID-19 pandemic time period prompted you to consider or desire to move to an alternative location? (Select all that apply.)

- Yes, to a location more rural or less urban than my current living situation (1)
- Yes, to a location less rural or more urban than my current living situation (2)
- Yes, to a location nearer to family or friends (3)
- Yes, to a location nearer to mountains (4)
- Yes, to a location nearer to the ocean or other water (i.e. rivers, lakes, streams) (5)
- Yes, to gain more outdoor space (i.e. to gain a private or shared yard) (6)
- Yes, to a location where I can work remotely but could not commute to/from (8)
- No, I have no desire to move or relocate (7)

|  |
| --- |

Q6.2 From the options provided, which best describes your opinion of the best education delivery method for children?

- Public School (1)
- Private or Charter School (2)
- Homeschool or Co-Op (3)
- Other, please specify (4) ________________________________________________

Q6.3 On a scale/ranking from 1 being “should be totally privatized/free of government intervention” to 7 “should be totally under the purview and responsibility of the government”, please state your management preferences for the following amenities:

|  | 1; should be totally privatized/free of government intervention (1) | 2 (2) | 3 (3) | 4 (4) | 5 (5) | 6 (6) | 7; should be totally under the purview and responsibility of the government (7) |
| --- | --- | --- | --- | --- | --- | --- | --- |
| Public ground transportation (such as busses and trains) (1) |  |  |  |  |  |  |  |
| Air travel (2) |  |  |  |  |  |  |  |
| Parks and recreation (e.g. bike trails or museums) (3) |  |  |  |  |  |  |  |
| Health, pharmaceutical, and medical services (4) |  |  |  |  |  |  |  |
| Banking and financial services (5) |  |  |  |  |  |  |  |
| Grocery and food procurement services (6) |  |  |  |  |  |  |  |
| Natural resource management (7) |  |  |  |  |  |  |  |
| Education (8) |  |  |  |  |  |  |  |
| Domestic police protection (9) |  |  |  |  |  |  |  |
| Fire services (10) |  |  |  |  |  |  |  |
| Military services (11) |  |  |  |  |  |  |  |

Q6.4 Did you, or are you aware of any friends or family members, who participated in or formed a pod (small groups of people who agree to study or socialize together, or share child care/education or other responsibilities) during the COVID-19 pandemic? (Check all that apply.)

- Yes, I participated (1)
- Yes, a member of my immediate family participated (2)
- Yes, a distant family member participated (3)
- Yes, a friend(s) participated (4)
- Yes, an acquaintance participated (5)
- I do not know of anyone personally, but I have heard of this behavior (6)
- No, I do not know of anyone doing this or have never heard of this behavior (7)

Q6.5 What would best describe your level of interest in social pods presently?

- I am already part of a social pod (1)
- I would have interest in forming a more permanent pod, such as through changing my living situation to facilitate communal aspects (2)
- I have actively sought to purchase land or otherwise invested in other communal living environments (3)
- I have considered forming a social pod (4)
- I have no interest in the concept of a social pod (5)

Q6.6 Please select the answer that best reflects your own time commitment or investment in the following activities:

|  | I would like to spend MORE time than I currently do (1) | I spend the appropriate amount of time on this activity (2) | I would like to spend LESS time than I currently do (3) | I have never participated in this activity; Not Applicable (4) |
| --- | --- | --- | --- | --- |
| Reading for leisure (1) |  |  |  |  |
| Reading for education/school (2) |  |  |  |  |
| Reading for work (3) |  |  |  |  |
| Facebook (4) |  |  |  |  |
| Twitter (5) |  |  |  |  |
| Instagram (6) |  |  |  |  |
| Snapchat (7) |  |  |  |  |
| TikTok (8) |  |  |  |  |
| YouTube (9) |  |  |  |  |
| Pintrest (10) |  |  |  |  |
| Watching television or streaming videos (including Netflix, Hulu) (12) |  |  |  |  |

Q6.7 Please rank your level of experience with the following activities by selecting all that apply.

|  | No experience (1) | Minor or limited experience (2) | Proficient (3) | I do this activity every season or regularly (4) | I do this activity occassionally (5) |
| --- | --- | --- | --- | --- | --- |
| Growing vegetables (1) |  |  |  |  |  |
| Growing fruit (2) |  |  |  |  |  |
| Raising own meat (3) |  |  |  |  |  |
| Raising own eggs (4) |  |  |  |  |  |
| Raising own milk (5) |  |  |  |  |  |
| Making or mixing own home health remedies (6) |  |  |  |  |  |

Q6.8 On a scale of 1 = no confidence at all to 7 = total confidence, score your view of the stability and sustainability of the following institutions:

|  | 1=No confidence at all (1) | 2 (2) | 3 (3) | 4 (4) | 5 (5) | 6 (6) | 7=Total Confidence (7) |
| --- | --- | --- | --- | --- | --- | --- | --- |
| The US financial system (including banks and federal reserve) (1) |  |  |  |  |  |  |  |
| The US food system (2) |  |  |  |  |  |  |  |
| The US military (3) |  |  |  |  |  |  |  |
| The US Federal Government (4) |  |  |  |  |  |  |  |
| Higher education (5) |  |  |  |  |  |  |  |
| The US technology sector (including Silicon Valley and Big Tech) (6) |  |  |  |  |  |  |  |
| The US economy (7) |  |  |  |  |  |  |  |
| Your State Government (9) |  |  |  |  |  |  |  |
| US Healthcare system (10) |  |  |  |  |  |  |  |
